# Decolonizing infectious disease programs: A mixed methods analysis of a novel multi-country virtual training for Female Genital Schistosomiasis

**DOI:** 10.1371/journal.pgph.0004235

**Published:** 2025-12-08

**Authors:** Kari Eller, Kelechi Amy Nwoku, Reda Sadki, Nicole Vecchio, Caroline Pensotti, Charlotte Njua Mbuh, Julie Jacobson

**Affiliations:** 1 Department of Lifelong Education, Administration, and Policy, University of Georgia, Athens, Georgia, United States of America; 2 Bridges to Development, Vashon, Washington, United States of America; 3 The Geneva Learning Foundation, Geneva, Switzerland; Brigham and Women's Hospital, UNITED STATES OF AMERICA

## Abstract

Existing medical curricula and continuing professional development for infectious diseases have been largely driven by materials from higher-income countries and exclude teaching about FGS. In 2023, The Geneva Learning Foundation’s peer learning-to-action model was used to create a multi-country virtual training program to address this gap and empower local healthcare workers in endemic countries. During Phase 1, participants learned about FGS and developed an action plan to address it. In Phase 2, participants received support in implementing their action plans. To explore the learning approach and its ability to reach a diverse set of health care workers, we conducted a mixed methods study framed in the theoretical lens of connectivism. Quantitative and qualitative data were collected through online surveys, analyzed separately, and then integrated. Healthcare workers from 19 Francophone African countries, representing all levels of the health system, participated in Phase 1. Over two-thirds of Phase 1 participants reported an increase in FGS technical knowledge and trained 2,675 colleagues. About 85% of the Phase 1 participants found the peer review process beneficial, generating new ideas that strengthened their action planning. Phase 1 course completion was not significantly associated with profession or organization where a participant worked. Social/external connections led to personal growth and high-level professional impacts. The networks formed created valuable support systems for participants, and training certification led to opportunities for role expansion and promotion. 255 and 71 participants completed Phase 1 and 2, respectively. All participants reported engaging and teaching 49,088 community members about FGS. Peer connections and local knowledge exchange addressed immediate educational needs across health system levels and promoted local action. The virtual peer learning-to-action model successfully reached diverse learners, equipping them with knowledge they directly applied to challenging problems in their contexts, effectively decolonizing the approach to FGS.

## Introduction

First described in 1899, Female Genital Schistosomiasis (FGS) is a chronic complication resulting from infection by *Schistosoma haematobium*, a parasitic worm [1]. Schistosomiasis is transmitted through contact with freshwater sources contaminated with schistosome larvae and manifests as an intestinal or urogenital disease. If untreated, schistosomiasis can lead to chronic inflammation and progressive urinary and genital tract pathology in women and girls, which can result in severe women’s sexual and reproductive health complications, including ectopic pregnancies, infertility, and spontaneous abortion [2]. The disease is associated with an increased risk of human immunodeficiency virus (HIV) and human papillomavirus (HPV) infections, contributing to stigma and social isolation [3]. Importantly, FGS is preventable, and early treatment options are available at a low cost [4,5].

Despite affecting millions of women and girls in endemic regions, FGS remains largely absent from medical and nursing curricula. Where schistosomiasis is taught, the focus is typically on urinary and intestinal forms, with little emphasis on the gynecological manifestations [6]. This curricular gap reflects a broader trend of neglecting the gendered dimensions of neglected tropical diseases (NTDs) [7]. FGS is also not included in sexual and reproductive health or HIV services in most endemic countries. Without guidelines and in the absence of FGS integration, (frontline) healthcare workers lack needed tools, resources, and standardized approaches for diagnosis, treatment, and referral [8,9]. In-service and continuing professional development (CPD) events help healthcare workers address knowledge gaps on conditions such as FGS, but in many low- and middle-income countries they are not mandatory and participation is limited by funding, infrastructure, and resource constraints [10]. As a result, in endemic areas, there is a high rate of misdiagnosis and mistreatment among those affected [11]. Clinical presentations such as genital lesions, discharge, and infertility are often mistaken for STIs or HIV-related conditions, exposing women to stigma and unnecessary treatments [6].

It has been argued that one reason for these problems is that medical practice and texts are often based on the ‘global standard’ established by high-income countries (HICs) [12]. This reliance can sideline the knowledge and priorities of endemic regions, where the burden of diseases such as FGS is greatest. Recent calls to decolonize global health have urged a critical reassessment of the power dynamics, assumptions, and practices that shape global health partnerships [12,13]. For example, the context of FGS, Water, Sanitation and Hygiene (WASH) is a critical area where discussions on decolonization have emerged [14]. Many WASH interventions have historically been guided by Western models that overlook local realities, leading to limited sustainability [14,15]. A decolonial approach instead calls for community-led, context-specific solutions that draw on local expertise and actively involve endemic communities in decision-making to ensure long-term impact [14,16].

### FGS peer-to-peer virtual training and support

Recognizing FGS as an important social and gender justice issue that needs to be tackled, two non-profits, The Geneva Learning Foundation (TGLF) and Bridges to Development, collaborated to design and implement a two-phase peer-to-peer virtual training course on FGS. In 2023, the training was offered to healthcare workers in Francophone Africa (S1 Text). The training course (Phase 1) and Impact Accelerator (Phase 2) were created using TGLF’s peer learning-to-action model [17–20]. This model, developed by co-author RS, is based on the core principles of informal and incidental learning [21], and optimizes the interdisciplinary and interprofessional sharing of knowledge using digital technologies [22]. The content for the training course was based on the FGS Competency Framework, which was developed by Bridges to Development in collaboration with diverse experts and the World Health Organization. This framework outlines a comprehensive set of 27 essential skills or competencies required for training healthcare workers at all system levels on FGS, covering diagnosis, treatment, and prevention in clinical and non-clinical settings [23].

**Fig 1:**
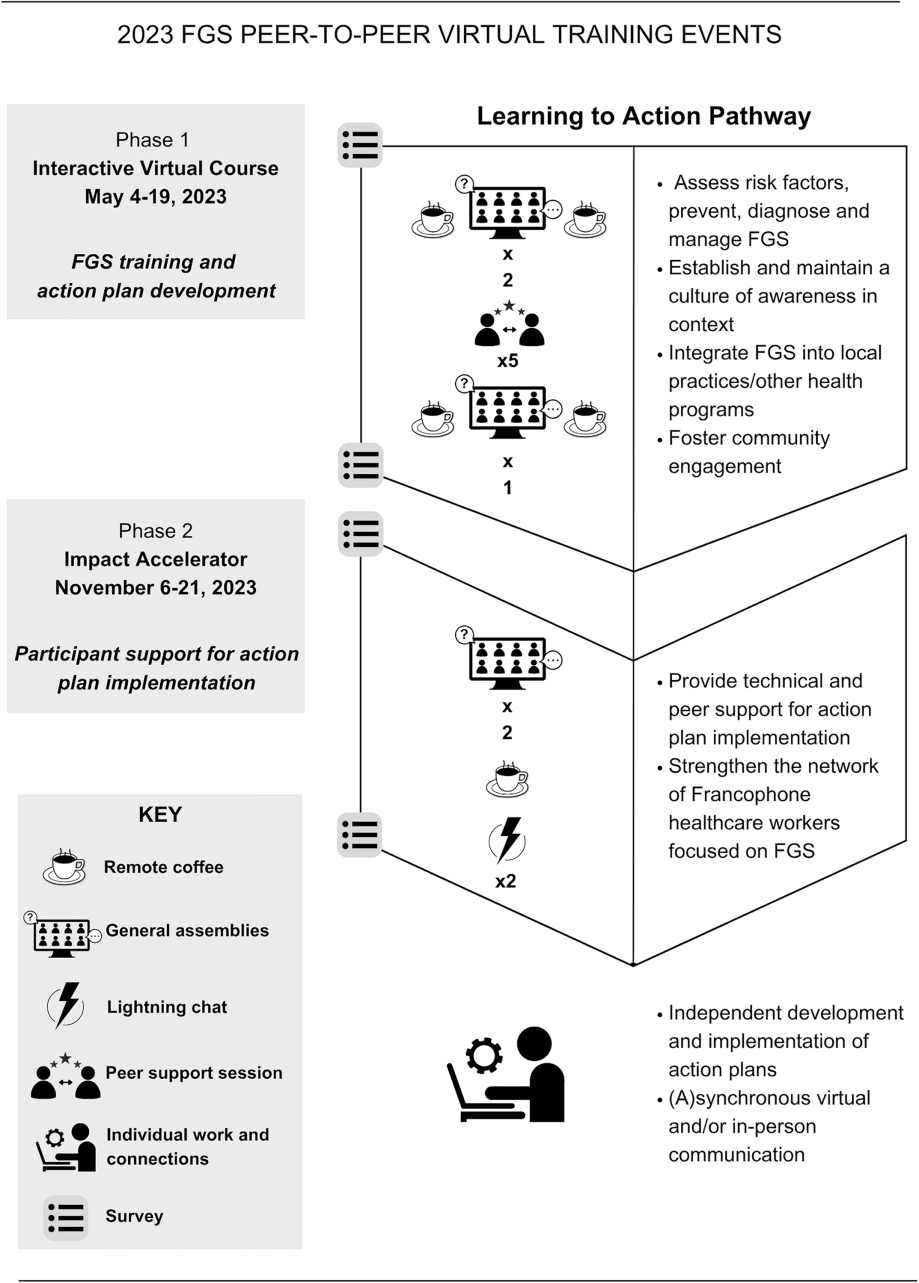
2023 Training event schedule and information.

Participation in the 2023 FGS peer-to-peer virtual training events was voluntary and free. Participants were selected based on their ability and availability to connect. Efforts were made to ensure diverse representation in terms of gender, health system tiers, and professional roles, with a focus on donor-prioritized areas, particularly in the Democratic Republic of the Congo. More males applied than females to the course; to ensure a more balanced gender representation, a higher proportion of female applicants were selected to participate. Events were carried out in French by TGLF with support from Bridges to Development staff and took place over a period of seven months. Subject matter experts presented the core FGS concepts and served as guides. Fig 1 depicts the overview of the events (S1 Text). During Phase 1, participants learned FGS core competencies and wrote an action plan to improve FGS outcomes in their communities. Action plans were developed and reviewed by participants and SMEs using a provided rubric. Guidance for developing and evaluating action plans focused on feasibility, adherence to national guidelines, integration with other health programs, and capacity for community engagement. During Phase 2, participants were provided additional support for implementing their action plans. Participants received and shared recommendations and resources from each other and SMEs. To increase our understanding of the model’s capacity to reach diverse learners and to fill critical gaps in FGS curricula and healthcare worker preparation, additional research is necessary.

### Theoretical framework

**Fig 2:**
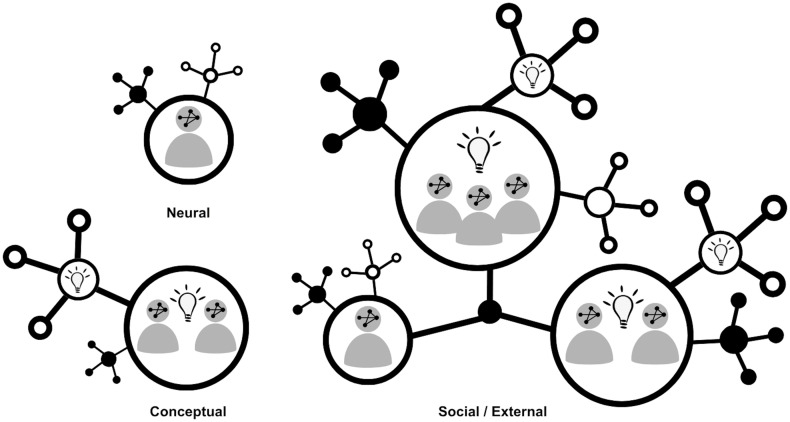
The three levels of connectivism.

Connectivism has been particularly influential in shaping online education [24] and thus offers a valuable lens for evaluating digital tools like TGLF’s peer learning-to-action model [17–20]. As visualized in Fig 2, connectivism understands learning to be a collaborative process linking individuals and information sources together. Connectivism’s focus on knowledge creation across individual, conceptual, and social/external levels [24,25] makes it an appropriate framework to examine how participants in the 2023 FGS peer-to-peer virtual training program leveraged digital technologies to access critical knowledge and collaborated to address complex health challenges related to FGS. On the neural level, biological networks allow us to form memories or concepts and attach meanings to them as we learn. On the conceptual level, we can generate new ideas by sharing and connecting information that resonates with others. On the social/external level, the networks we are a part of influence the resources we have access to and the connections we can make [26,27].

### Research purpose and questions

This study aims to explore the learning approach, document, and analyze the implementation and outcomes of the 2023 FGS peer-to-peer virtual training program for Francophone Africa. Specifically, it asks: through the lens of connectivism, how did the 2023 FGS virtual peer-to-peer training course (Phase 1) and Impact Accelerator (Phase 2) reach and create value for diverse participants and result in local action? The following sub-questions were developed to measure value and impact:

#### Quantitative Sub-questions.

1. What factors influenced the likelihood of participants completing either phase?
2. To what extent did the training phases achieve their intended outcomes, and how did TGLF’s peer learning-to-action model contribute to these achievements?

#### Qualitative Sub-questions.

1. What learning, connections, and resources created value for participants or subgroups of participants?
  1. What technical knowledge and skills did participants report learning?
  2. In what ways did participants share how peer learning and networking with others strengthened their action plans?
  3. How did participants’ accounts of diverse connections and resources improve their personal growth and high-level professional impacts?
  4. How did those who completed their action plans and those who did not describe their experiences differently?

## Methods

### Ethics statement

This study is based on secondary data collected by The Geneva Learning Foundation (TGLF) with oversight from its Commission on Research Ethics (CRE). TGLF’s CRE abides by the principles of the Cantonal Commission for Research Ethics (CCER), Federal Law on Research on Human Beings (RS 810.30), Swiss Human Research Act (HRA) and Ordinance on Organisational Aspects of the Human Research Act (HRA Organisation Ordinance, OrgO-HRA) for all data collection, management, and its protection, and authorizes proposed research. On June 18, 2024, co-author KE received approval from TGLF’s CRE to conduct a research project titled “Enstorying Global Health Landscapes of Learning” on the influence of TGLF’s peer-learning model. This study forms part of the TGLF CRE-approved research project. On July 12, 2024, the University of Georgia Institutional Review Board approved and designated the project as non-human subjects research (PROJECT00009825).

### Research design

**Fig 3:**
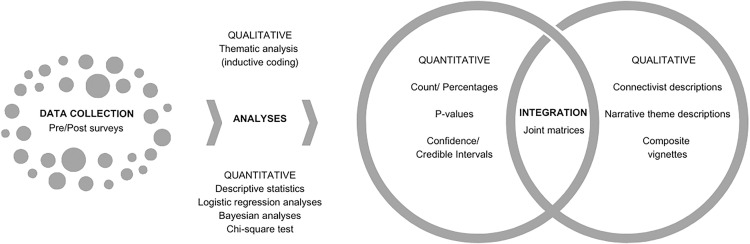
Procedural diagram of research.

This research employs a convergent, parallel mixed methods design [28,29], giving equal weight to both strands of data (QUAN+QUAL). Quantitative and qualitative secondary data from both phases of the 2023 FGS peer-to-peer virtual training program were collected simultaneously, analyzed separately, and then integrated [30]. To achieve the research aim, quantitative and qualitative research methods were triangulated to provide a more comprehensive interpretation of the data (Fig 3).

### Data collection

Data collection included pre- and post-course online surveys developed by TGLF for both phases of the 2023 FGS peer-to-peer virtual training program. Links to the surveys, which were built using TypeForm [31] were provided to all participants via email. Participants completing the surveys were French-speaking interprofessionals from all levels of the health system who participated in the 2023 training events. Before surveying participants, TGLF informed them in writing of the survey purpose and how their responses may be used (e.g., reports, presentations, research). Within the survey, participants provided written consent for their data to be used for research. Participants voluntarily completed surveys and were not compensated for doing so. They could skip any questions they did not wish to answer. Co-authors JJ, NV, RS, and CM had access to respondents’ identifying information during and after data collection. Linked videos and texts from participants, including those shared during the lightning chats, are publicly available. Pre-course survey data collected were compiled on June 27, 2023, and post-course survey data on December 12, 2023.

All surveys included both quantitative and qualitative elements and captured participants’ self-reported changes in knowledge about FGS, access to FGS tools/resources, and their FGS network connection(s). The Phase 1 pre-survey also requested participants’ sociodemographic information and healthcare provision details, as well as their program expectations and ability to participate. The Phase 2 pre-survey requested updated information on these areas from the Phase 1 pre-survey, participants’ collaboration with others in the program, and action plan developments, such as current needs and steps taken. Phase 1 and Phase 2 post-surveys additionally captured action plan developments and requested information from participants on their experience in the program, such as any difficulties they encountered and how they felt supported in their personal and professional development.

### Data analysis

Anonymized versions of the survey data sets were shared with all co-authors on January 12, 2024, for a report to the training funder. Secondary data analysis for research began on July 12, 2024. Quantitative and qualitative analyses occurred separately, followed by an integrative analysis.

#### Quantitative analysis.

Quantitative statistical analysis conducted included descriptive statistics, and multiple logistic and Bayesian regressions. Relevant columns were selected from the original dataset to include only variables pertinent to the study, and the regression models incorporated several covariates (gender, profession, organization type, and prior experience), which addressed confounding variables. Some variables, such as profession and organization, were transformed to address issues of sparsity in categories and to facilitate meaningful statistical comparisons. The numerical values of certain variables, such as the impact of participation on social relationships, were recoded into categorical variables to ensure interpretability and consistency.

Descriptive statistics were used to summarize pre- and post-training knowledge scores and self-reported competencies, which were recorded as individual responses graded on a scale from 0 to 5. Changes in these measures provided insight into learning gains attributable to the training. Multiple logistic regression enabled the identification of significant predictors of training completion and to model the probability of improved knowledge based on individual characteristics. A backward stepwise approach was employed using the stats package in R (version 4.4.2). Initially, all candidate variables were included in the model, and variables were systematically removed one at a time based on their p-values and the Akaike information criterion of the models. Variables with unreliable or undefined p-values were excluded during this process to ensure the stability and interpretability of the models. We also assessed multicollinearity using the variance inflation factor (VIF) and excluded variables with high VIF values (> 5) to prevent issues with coefficient instability. For the final models, the threshold of significance was a p-value below 0.005.

Given the smaller sample size for Phase 2, Bayesian logistic regression was applied to estimate the effects of peer learning activities on outcomes such as knowledge acquisition. It was performed using the rstanarm package, which employs Hamiltonian Monte Carlo for efficient posterior sampling. The results were summarized using the posterior mean of the coefficients and their 95% credible intervals (CIs). Significant predictors were those whose CIs did not include zero. Bayes factors were calculated to compare model evidence, identifying the best-fitting models. Trace plots showed smooth mixing and stable chains, confirming convergence and the reliability of posterior estimates.

#### Qualitative analysis.

Qualitative analysis followed the six-phase process of thematic analysis described by Braun and Clarke [32] and was conducted in Excel. Open-ended survey responses were translated from French to English using a neural machine translation service [33]. Selected responses were reviewed and corrected for accuracy and intent by TGLF and Bridges to Development staff, who are fluent in both French and English.

The first phase involved reading the data several times to clarify and organize responses. In the second phase, data were inductively coded. Preliminary codes were assigned and discussed with the team. In the third phase, codes were combined and categorized into three main groups: challenges, strategies, and results (Table A in S3 Text). For example, the codes ‘addressing reluctance’ and ‘site safety and stability’ were categorized as ‘implementation challenges’, the codes ‘advocacy with key health leaders’ and ‘phasing financing/activities’ were categorized as ‘implementation strategies,’ and the codes ‘outputs’ and ‘outcomes’ were categorized as ‘implementation results.’ The categories were then discussed by the team in relation to participants who completed their action plans and those who did not, and considered through the theoretical lens of connectivism. In the fourth phase, the team reviewed all the data to discuss possible themes. Two themes were created in the fifth phase, and responses connected to each theme were identified. In the sixth phase, verbatim quotes were selected to represent each level of connectivism, and composite vignettes of healthcare workers’ experiences were written to portray the complexity of themes in an accessible manner for diverse readers [34,35]. Initial drafts of the two composite vignettes were written with assistance from ChatGPT (25 February 2024). The decision to begin with AI-generated initial drafts was made to help populate quotations at random from the selections that may otherwise not have been chosen due to the number and length of participant responses. The initial drafts underwent extensive revisions to present key thematic elements in a focused and holistic manner, while also ensuring the sociodemographic diversity of the verbatim quotes. The final vignettes share synthesized narratives of each theme.

#### Integrative analysis.

Integrative analysis began by merging quantitative and qualitative data in joint matrices [30,36] related to the study’s aim. Merged data juxtaposed descriptive statistics alongside qualitative insight and quotes associated with connectivism levels. The data were then analyzed to assess how they confirmed, expanded, complemented, or diverged from each other and generate meta-inferences. An integrated narrative was then written to synthesize the meta-inferences [30,36–39].

### Positionality and reflexivity

As the research team responsible for this collaborative investigation between our organizations, Bridges to Development and TGLF, we are committed to decolonizing global health. Our organizations emphasize local knowledge, diverse representation, and the empowerment of healthcare professionals in their respective contexts. In keeping with this commitment, we acknowledged and reflected on the dimensions of our identities relevant to this research in a version of Khan’s transparency matrix [40] that we adapted to fit our work (S1 Data).

Collectively, we are a diverse group representing various ethnicities, professions, and levels of expertise in the field of public health. Our organizations have long been involved with the issue of FGS and have worked directly on the ground in some of the affected countries. We are united in our desire to empower and equip healthcare workers in the communities they serve (S2 Text). Our experiences in virtual training using TGLF’s digital peer-learning methodology have been positive, and we value the type of engagement and outcomes reported by participants. Throughout our own processes of sharing, we learned from and with one another, and viewed ourselves as having a keen understanding of the methodology and FGS contextual challenges and opportunities; a view we recognized could skew our analysis. Thus, during all stages of our research process, we met regularly to analyze the data, respectfully challenging each other’s understandings and holding each other accountable to ensuring that our analysis remained grounded in the data. To increase the trustworthiness of our results, we triangulated findings by cross-referencing multiple data sources and sharing verbatim quotes. We also conducted an audit of the results, ensuring that colleagues from the Global South, who are integral members of our team and program facilitators, reviewed both its content and presentation, and created an unofficial translation of the accepted manuscript (S4 Text) and supporting information (S5 Text) in French.

## Results

### Quantitative findings

#### Study participants and characteristics.

Table B in S3 Text shows the characteristics of the participants. A total of 1,686 health professionals applied for Phase 1. Of these, 786 participants were accepted, and 255 completed the Phase 1 course. There were 564 women applicants compared to 1,115 men. To promote gender equity, 386 women and 397 men were accepted. Most accepted participants were aged 35 years and above (63.6%). The largest proportion resided in Central/Middle Africa (48.7%), including Cameroon, the Central African Republic, the Congo, the Democratic Republic of the Congo, Equatorial Guinea, and Gabon. Participants represented diverse health professions, with the largest group being Doctors/Obstetrician-Gynecologists (43.4%), followed by Public Health Officers (21.8%) and Nurses/Midwives/Nurse Practitioners (21.2%). Participants also worked across a range of institutions, most commonly in public hospitals and health facilities (23.1%), followed by national-level Ministry of Health offices (20.3%) and private organizations (20.8%). For Phase 2, 145 professionals applied, and all were accepted for participation, with 71 completing the course. Again, more men applied, making up 64.6% of applicants compared to 35.4% women.

#### Factors influencing course completion.

Table C in S3 Text shows the factors that influenced the completion of both phases. Despite selecting more women for the training to ensure a more balanced cohort, women had less than half the odds of completing Phase 1 compared to men (OR: 0.42, 95% CI: [0.22, 0.79], p = 0.008). Participants who did not find the concepts or training technology difficult had more than three times the odds of completing Phase 1 compared to those who had difficulties with concepts or training materials (OR: 3.45, 95% CI: [1.15, 10.34], p = 0.027). Participants who paid out-of-pocket for their expenses (i.e., internet connectivity, action plan implementation) had more than twice the odds of completing Phase 1 compared to those who neither they nor their employers incurred any costs (OR = 2.45, CI: [1.26, 4.78], p = 0.008).

For Phase 2, participants who had already begun implementing their action plans had higher log-odds of completion compared to those who had not yet started (coef: -1.19, 95% CrI: 0.132.35). In contrast, community health workers showed lower log-odds of completing Phase 2 relative to doctors (coef: -1.75, 95% CrI: -3.58, -0.10).

#### Effectiveness of training: Knowledge and capacity development.

Table D in S3 Text shows the training outcomes of both Phase 1 and Phase 2 training courses. Among the accepted participants for Phase 1, 14% had never heard of FGS and 62% had no prior experience with it. Phase 1 focused on increasing knowledge and capacity. 66% of participants reported at least a two-level increase in knowledge and confidence on the 0–5 scale. The proportion of participants who reported comprehensive knowledge of FGS increased from 18.0% to 47.2%. The proportion of participants who felt able to diagnose FGS rose from 41% to 90%, those who felt able to treat from 37.1% to 86.4%, and those who felt able to prevent from 57.5% to 96.6%. The proportion of participants who reported full confidence in discussing FGS with patients also increased from 19.8% to 55.9%.

Table F in S3 Text presents the factors that influenced the likelihood of gaining more than one level in reported knowledge levels after Phase 1. Community health workers had 83% lower odds of achieving this compared to doctors/obstetrician-gynecologists (OR: 0.17, 95% CI: [0.04, 0.67], p = 0.012). Similarly, participants with prior experience managing female genital schistosomiasis had 83% lower odds of reporting such gains, compared to those who did not (OR: 0.17, 95% CI: [0.04, 0.71], p = 0.015).

Table E in S3 Text presents the broader impact of the training beyond the participants. After Phase 2, 39% of participants diagnosed or managed patients with FGS, amounting to approximately 638 cases. 91% of participants reported training a total of 2,675 colleagues. Additionally, 82% of participants engaged with their communities, reaching over 49,000 individuals with information about FGS.

#### Effectiveness of training: Action plan development and implementation.

Of the 232 action plans developed during Phase 1, 82% included strategies for integration with other programs, such as HPV/cervical cancer, WASH, infertility, STIs, HIV/AIDS, schools, perinatal care, NTD programs, immunization, and migration/immigration (Fig A in S3 Text).

Phase 2 focused on providing support for their action plans. As shown in Table D in S3 Text, at the end of Phase 1, 39% of participants had begun implementing their action plans, but only 1% had completed them by the time of the post-course survey. Before Phase 2, there was a marginal increase in reporting action plans completed among those who participated in Phase 2. However, after Phase 2, 23% had completed their action plans, and 71% had begun their implementation by the time of the post-course survey.

Fig B in S3 Text presents the three main categories of action plans developed by participants in Phase 2. The Awareness and Prevention group focused on raising awareness about FGS and promoting preventive measures. The Diagnosis and Case Management group addressed improving diagnostic capacity, treating FGS cases, managing referrals, and registering cases for monitoring. The Comprehensive Management group combined multiple objectives, including awareness, prevention, diagnosis, treatment, referral, and registration. To identify who developed each type of action plan, we examined participants’ professions and found clear alignments (Fig C in S3 Text). Doctors, representing nearly 25%, accounted for most of the comprehensive management plans. Community health workers all focused on awareness and prevention, while laboratory technicians exclusively developed plans on diagnosis and case management. This alignment was also evident when considering whether participants’ roles included pelvic exams. Among those with objectives in diagnosis and case management, 60% reported conducting pelvic exams as part of their duties. The proportion increased to 68.4% for participants with comprehensive management objectives. By contrast, only 35.9% of participants with awareness and prevention objectives reported pelvic exams in their professional activities, reflecting the lesser clinical emphasis of this group.

#### Impact of TGLF’s peer learning-to-action model.

Table G in S3 Text presents the results of Bayesian regression models examining the influence of TGLF’s peer learning-to-action model [17–20]. Knowledge acquisition was positively influenced by peer support. Those who reported peer support as useful had higher log-odds of knowledge acquisition compared to those who did not report peer support as useful (coef: 1.97, 95% CrI 0.80, 3.30).

Among Phase 1 participants, 61% reported in the post-training survey that they learned more than they had anticipated from the peer reviews. In addition, 86% stated that the peer reviews significantly improved their own action plans, while 85% found reviewing their colleagues’ action plans to be beneficial. 85% indicated that they experienced significant changes in their professional practice because of participation.

A substantial proportion of participants (77.2%) contacted their peers after Phase 1 for collaborative purposes. 61% of these participants also credited maintaining contact with their peers after the program to the peer support model. These collaborations included developing joint projects, providing implementation assistance, supporting ongoing initiatives, and working together on new projects (Fig D in S3 Text).

### Qualitative findings

#### Neural connections: FGS technical knowledge and skills learned.

Participants entered Phase 1 with varying levels of knowledge regarding FGS diagnosis, treatment, and prevention. Some participants, despite being at the frontlines or involved in policymaking, had no prior knowledge of FGS. The information they received strengthened their overall knowledge of the disease and diagnosis capacities, better preparing them to discuss and treat cases beyond the mass distribution of drugs. Participants also explained that the training helped correct misinformation and misdiagnoses. They recognized the positive impact of this newfound knowledge on their clinical practices and in the lives of the patients who benefitted from their improved expertise. Therefore, they extended the reach of the training and enabled even more people to gain knowledge and skills related to FGS.

“*More than 500 people (were) made aware of the disease, 103 healthcare providers trained on the definition and rapid detection of cases in five health facilities including two private (ones).*” - Doctor working with a nonprofit

#### Conceptual connections: Peer learning and networking to strengthen action planning.

Participants overwhelmingly found peer support to be valuable throughout the training. Some valued the peer interaction as more beneficial than the provided materials. Participants also reported that the development of their action plans was improved both by the formal peer review process and through informal networking with peers outside the official channels. This peer interaction helped participants clarify expectations and allowed them to learn from each other’s successes and challenges. Participants also credited the review process for providing the support and feedback necessary to improve the presentation of their ideas.

“*The peer review allowed me to improve my action plan. For example, a peer reminded me that the title of my plan had to start with an action verb. Another suggested that I find a better map of the health districts…because the one I had included in the initial action plan was not too expressive. These two examples, to name just a few, demonstrate the extent to which peer review remains essential.*” –Public health officer at a non-profit

Many participants reported that continuing to engage with their peers after the end of Phase 1 played a crucial role in implementing their action plans. They described learning from the testimonials of others through various platforms, gaining insights into how to effectively implement their plans. Those who successfully completed their action plans emphasized that integration with existing programs was key to their success, providing them with the necessary materials and data to coordinate responses across different entities (Fig A in S3 Text). Participants who faced challenges sought advice from their peers.

“*She helped me not to get discouraged but rather to follow through with my plan despite the complications by adapting it according to the situation found.*” - Doctor in a private hospital/health facility

Common obstacles included the unavailability of medication, lack of funding, and the need to secure buy-in from health authorities. They addressed these barriers by advocating with health officials and community leaders, exchanging advice and solutions with their peers, and consulting with experts and mentors. The peer-driven support networks developed in each country were instrumental in overcoming implementation challenges and achieving their goals.

“*When I was unable to deploy my action plan due to lack of financial means, I seemed disoriented, until by following live testimonies and reading via Telegram, (I learned) the methods used by other scholars who did not have the financial means like me, to reach populations at risk. This knowledge made me change my approach. It is clear that when we participate in such a launchpad (the Impact Accelerator)...we (are) influenced by new ideas, (and) other scholars from diverse backgrounds*.” –Community health worker working at the district level

#### Social/external connections: Diverse connections and resources for growth and impacts.

Peer interactions broadened the viewpoints of participants in several ways. They fostered recognition of how local realities connect to broader regional patterns, including shared structural challenges such as limited water access and its health consequences. They also encouraged a new awareness of approaching diseases like FGS through a collaborative, ecosystemic approach. The participants credited the peer interactions with increasing their confidence and enabling them to believe in their capacity to effect change within their communities and spheres of influence. These interactions not only increased their desire to expand their knowledge of FGS but also motivated them to share this knowledge with their communities. Additionally, participants reported strengthened communication capacities, including active listening, tolerance, and the ability to synthesize ideas concisely under time constraints. They described personal growth in critical thinking, particularly in recognizing the value of cross-border collaboration for addressing health challenges at scale.

“*This training allowed me to understand that I am now a citizen of the world and that my single advice to a colleague can be useful to them in addressing a public health problem or saving a life on the other side of the world.*” –Public health officer working at a non-profit

Participants further explained how certification opened opportunities for role expansion and promotion. Some were given added responsibilities such as district-level FGS case monitoring, while others experienced increased patient demand and greater community trust. They also described becoming more proactive in their professional roles, which improved their work performance. The training expanded their professional networks within and across countries, creating support systems that enabled consultation on FGS cases and quicker access to information. These networks facilitated collaboration on initiatives, strengthening their ability to advocate with health authorities and influence policy and practice by promoting access to testing, treatment, and support services for affected women

“*People listen when I talk about FGS, and women want to be ‘screened’ for FGS.*” - Doctor working in a public hospital/health facility

#### Thematic findings.

Analysis of the code categories between participants who completed their action plans and those who did not revealed two themes. The first theme, “accelerating connections and integration,” emerged as a common thread among individuals who successfully completed their action plans. In contrast, the second theme, “connectivistic connections despite complexities,” was found to be the prevailing experience among those who did not complete their plans.

**Theme 1: Accelerating Connections and Integration:** Among participants who completed action plans, the most common strategy employed was integration of activities. Integration provided the needed materials and data to coordinate an accelerated response among different entities. To accomplish integration, participants credited new and enhanced connections to information, peers, and subject matter experts through the FGS virtual training events. These connections strengthened their advocacy to healthcare leaders with authoritative power and enabled them to carry out action plan activities, whether a budget existed or not. FGS integration spanned awareness raising, diagnosis, and treatment from local to national levels and was facilitated by collaboration among community agents. The integration took place within both private and public routine healthcare services, including maternal health services in specific areas, and was also incorporated into programming for neglected tropical diseases. By integrating activities, participants reported that implementing their action plans led to improvements in healthcare outreach and local actions.

***Composite Vignette (Theme 1)*.** Participating in the training events, I learned that FGS is “*a neglected disease.*” Conversations I had with other scholars in “*FGS endemic and non-endemic areas*” allowed me to “*have a global look at the epidemiology of FGS in the world*” to discover how it has been “previously ignored and yet (is) common in our community.” I felt compelled to share it with my colleagues and developed “*PowerPoint presentations on FGS.*” After listening to what I had learned, they were “*convinced like me that a few things can be done to further protect women and girls against certain diseases.*” We set to work “*sensitizing the zonal medical officer, hospital director, and data manager of the health zone…Despite the challenge of “not hav(ing) enough resources,*” they suggested leading “*advocacy with the partner who supports the fight against NTDs and certainly capitalize on the resources given for other NTDs.*” Working together, we began training during regular clinical meetings using the “*World Health Organization Atlas*” as a guide. We also set up a “*WhatsApp alert group*” for real-time reporting of suspected FGS cases. We organized “*communications through radio*” and collaborated with community relays, non-governmental organizations “*agents of the youth centers, and the medical school*”. With so many activities going on, support and guidance from experts during the learning cycle were invaluable in “*choosing action*s” and fellow scholars’ advice “*strengthened our capacity…to work effectively and efficiently*”. Through our efforts, we have seen a “c*hange in behavior of the population*,” and we have now broadened our “*differential diagnosis hypotheses*”. “*Bilharzia (FGS) is an important parasitic (infection) endemic in the world,*” and “*the accelerator was like a watchdog, or a rooster who came to encourage us to run ahead of time,*” helping us to accomplish our plan.

**Theme 2: Connectivistic Connections Despite Complexities:** Participants who did not complete their action plans reported making progress and needing more time to integrate activities into local and national plans. Reasons for the additional time varied, including the desire to base projects on data not yet collected or shared, waiting for funding decisions, obtaining permission from health authorities to proceed, and needing to adjust the implementation timeframe due to personal reasons or other community activities. In several cases, action plans were developed to span multiple regions, resulting in a broader impact that extended beyond the allotted timeframe for implementation. Additionally, in some areas, the unavailability of medication to treat FGS hindered the completion of action plans. In these circumstances, participants found it useful to request assistance from health authorities and community leaders, share advice and ideas with their peers, and consult with experts/mentors. By adopting a stepped approach to action planning, participants pushed forward while waiting and found alternative ways to improve healthcare outreach and local action.

***Composite Vignette (Theme 2)*.** I began “*as quickly as possible to roll out*” my plan during the Impact Accelerator. However, at every turn, it felt like I hit a wall. I found some “*data or usable documents*” related to FGS in our community, but they were not enough. My meetings with health and community leaders generated interest, but the proposal was “*blocked at the level of authorizations*”. “*This accelerator has helped me a lot, especially in planning activities.*” By connecting with peers, I had “*many other ideas*” and strategies. For example, “*I carried out surveys to see the proportion of women affected*”. Then, I made a written “*request to the leadership to obtain authorization to implement our plan*”. The reply asked me “*to train staff in the use of this medication and on FGS*”. I joined “*forces with other health professionals*,” and we presented “*on FGS during morning work meetings in health structures*”. We used these opportunities to help clarify “*the confusion between FGS and sexually transmitted infections*,” and we discussed “*FGS widely in the form of a debate, where everyone gives their point of view based on experience and scientific data*”. “*To learn more from their professional experiences*,” we made plans for future online training to continue conversations. Health leaders are now beginning “*to encourage its popularization and…take ownership of it*”. We “*have requests that are being processed*,” but for now, we are making “*do with what little we could raise*”. So far, “*we have 10 cases of FGS treated and 21 health workers trained, and 24 community workers oriented*”. “*Thanks to this participation, I knew nothing about FGS, but today, I have become a great teacher. Thanks to this participation, I am connected to a world of experts who can help me solve a problem on FGS*”. “*We will not finish within the deadline as planned*,” but “*the plan is underway*”.

### Integrated findings

As described in previous sections, Phases 1 and 2 of the 2023 FGS virtual peer-to-peer training created value for participants across gender, profession, health system role, and other demographic backgrounds. Participants benefited by gaining knowledge and awareness, with most taking action and making progress even when action plans were not fully completed. Synthesizing these meta-inferences offers a deeper and more comprehensive understanding of the program’s connectivistic impact. Table H in S3 Text illustrates how the training increased participants’ knowledge and confidence, while extending its impact through the participants’ actions on patient care, peer training, and community engagement. Table I in S3 Text shows how action plans were developed, integrated, and implemented, highlighting their alignment with professional roles, the central role of integration, and the peer-driven processes that enabled implementation despite barriers. Table J in S3 Text shows how peer support and interactions strengthened knowledge, professional practice, and action plan development, while fostering continued engagement through networks that sustained collaboration, learning, and advocacy beyond the training.

## Discussion

This mixed-methods study shares the approach and methods of FGS training using TGLF’s peer learning-to-action model [17–20]. The findings of this study demonstrate how the training approach successfully reached a diverse group of healthcare workers who found value in the training and took action that created wider influence based on the knowledge gained.

Peer-assisted or peer-to-peer learning has been widely utilized in teaching health professionals and has been argued to have numerous benefits, including social, behavioral, and cognitive improvements [41,42]. TGLF’s model draws upon peer-assisted learning and also incorporates elements of action learning. McGill and Brockbank [43] describe action learning as a continuous process of learning and reflection to achieve practical outcomes. The TGLF model incorporates the reflective and practical elements of action learning, while placing greater emphasis on peer-assisted learning rather than relying solely on a facilitator or expert for instruction [17,18,44,45]. The findings of this study show that the training, using the model and the adapted FGS content developed by Bridges, helped participants feel more confident in discussing, diagnosing, and managing FGS. In line with the findings of Stone and colleagues [46], they also described becoming better at listening, thinking critically, communicating, acting proactively, performing in their roles, and advocating effectively.

This peer-learning-to-action approach aligns with the theory of connectivism, which emphasizes that diverse connections are central to continuous learning and the generation of new ideas [24–26]. In this study, authentic connections were cultivated during training, creating an inclusive and interactive environment that supported ongoing learning, reflection, feedback, and collaboration [47–49]. National and cross-border collaborations provided participants with support for action plan implementation, rapid access to information, and collective platforms for advocacy both during and after the training program. This reflects the connectivistic view that knowledge does not reside in a single individual but emerges from the strength of networks and shared practice.

McGill and Brockbank [43] note that action learning benefits both individuals and organizations. Our study shows how training through the TGLF model led to local actions that influenced community health: patients were reached, colleagues trained, and communities engaged. Participants also built credibility and professional trust. Hence, the program achieved its aim of promoting equitable access to information and resources, while enabling the development of action plans that positioned participants to lead health transformation initiatives meaningful to their own contexts.

Makau-Barasa et al. [50] argue that NTD interventions are weakened by limited contextual understanding and poor community involvement. This training shifted agency to local professionals, valuing their knowledge and supporting actions emerging from within affected communities. Kwete et al. [51] highlight the assumption that healthcare workers in low- and middle-income countries (LMICs) cannot address their own health challenges. Our findings counter this assumption and align with other scholarship [52–55] showing that LMIC professionals can develop culturally appropriate, sustainable solutions. Yet most global health campaigns, including those for malaria, HIV/AIDS, and tuberculosis, remain led by institutions in the Global North. Some scholars argue against health organizations prioritizing technology-based solutions to health challenges in the Global South, as these often deliver short-term rather than lasting improvements [56]. Greater emphasis is instead placed on systems building, local capacity strengthening, and sustainable alternatives, which this training supports. While we facilitated the platform for this training, the process was not characterized by a ‘white saviour’ dynamic; rather, participants taught and empowered one another to design and implement locally driven actions.

Previous studies have underscored the importance of embedding disease- or sector-specific interventions into broader health services to increase health coverage and efficiency, such as the integration of FGS initiatives into HIV, WASH, and cervical cancer services [4,23,57,58]. Therefore, integration with existing services was prioritized by many participants during Phase 1, as a culturally appropriate method to achieve their goals. Participants identified more than 10 opportunities for program integration from their diverse perspectives, particularly HPV/cervical cancer, WASH, infertility, STIs, and HIV/AIDS programs. Interestingly, the NTD program integration ranked eighth on the list, highlighting the importance of providing training across various professions, institutions, levels of the health system, and organizations to identify new opportunities for integration.

Our findings add to the evidence on challenges affecting the implementation of NTD interventions in LMICs. Participants encountered barriers such as resource gaps, delays in authorization, and shortages of essential medications. Limited access to medicines and healthcare remains a major obstacle to NTD elimination [59]. The WHO’s NTD 2021–2030 Roadmap [60] calls for a multisectoral, systems-thinking approach to overcome these barriers. This study contributes to that agenda by addressing one critical component—strengthening the knowledge and capacity of healthcare workers. By brainstorming ways to address these challenging factors with diverse peers and learning new strategies from their network connections, participants built individual and collective capacity to respond, and, in some instances, improved local actions beyond original plans.

This training successfully reached diverse learners across multiple countries. However, despite our efforts to promote diversity, women and community health workers were less likely to apply for and complete the program. One possible reason could be time constraints due to added responsibilities [53,61–63]. Historically imbalanced trends in training completion explain how women are less likely to complete programs due to additional responsibilities [63]. Community health workers were also consistently less likely to complete either course than doctors, and were associated with lower odds of having learning gains after Phase 1. Community health workers are crucial frontline workers who frequently engage with grassroots communities [52]. Their underrepresentation is a limiting factor in the diversity of knowledge and experience shared about ways to raise awareness, diagnose, refer, and treat FGS, as well as to perform grassroots routine disease surveillance in communities. Furthering our understanding of their experiences could improve the training model and adaptations to support and strengthen their participation.

## Limitations

We acknowledge several limitations to our study. Our research used self-reported measures, which could introduce a response bias. While a response rate of 31% was sufficient for these analyses, and we achieved response rates of 42% for Phase 1 and 49% for Phase 2, higher response rates may have provided a more comprehensive picture. With our methodology, we were not able to gain further insights into the reasons women and community health workers were less likely to complete the course. We also could not follow the impact of the action plans on communities, the health system, or patient outcomes. Additionally, while the context of this study is representative of diverse global health landscapes in Francophone Africa, findings may not be generalizable to other areas. However, we expect the insights gained from this research to have transferable value in other contexts and programs.

## Conclusion

Peer learning programs, like the 2023 FGS virtual training, strengthen medical education partnerships by connecting health professionals across geographic and professional boundaries. By decentering expert knowledge, fostering peer-to-peer connections, and prioritizing the sharing of localized knowledge, this training program addressed immediate educational needs while contributing to a broader, more equitable framework for global health education. For Bridges to Development and TGLF, this is a significant step toward decolonizing global health. The goal is to empower local practitioners, value their contributions, and promote sustainable, context-specific solutions that can improve health and healthcare outcomes. This study demonstrates that peer learning programs utilizing digital technologies foster community collaboration and establish global support networks and partnerships, enabling professionals to exchange advice, share experiences, and collectively address common challenges. These connections promote innovation and capacity building through interdisciplinary and interprofessional exchange in healthcare. As aptly put by a participant in this study, “*(When we) bring together more than twenty nationalities in an interactive way on a virtual platform…we can influence the world*”.

## Recommendations

Based on insights gained from this research, specific recommendations for infectious disease training programs include: (1) harness the potential of participatory methodologies and digital technologies to dismantle historical power imbalances in global health and foster truly sustainable changes within local communities, (2) evaluate how action plans benefit communities, the health system, and patients, (3) support participants in creating effective strategies and communications to advocate for the integration of their action plans into current existing services and programs, and (4) increase collaborative research with participants to identify learning barriers to full engagement.

Contextual factors and rapidly evolving threats to global health, like climate change, challenge the ability of global health practitioners and systems to prevent, treat, and mitigate infectious diseases [61]. Therefore, we further recommend adopting TGLF’s peer learning-to-action approach using digital technologies [17–20] to address additional health challenges. Doing so will bring new perspectives, knowledge, and lived experiences into global dialogue and accelerate problem-solving processes [44,64]. Such participatory processes also encourage needed mutuality and accountability to restore relationships and improve outreach and outcomes [65–67]. For neglected diseases like FGS, our research suggests that this approach fosters more inclusive and equitable partnerships, scales community-level action, and enhances the capacity of global systems to respond.

## Supporting information

S1 Text Additional training information. (PDF)

S1 Data The FGS transparency matrix. (XLSX)

S2 Text Partner media connections. (PDF)

S3 Text Additional figures and tables. (PDF)

S4 Text Manuscrit en français. (PDF)

S5 Text Informations complémentaires. (PDF)
