# Supplementary material for: Decolonizing infectious disease programs: A mixed methods analysis of a novel multi-country virtual training for Female Genital Schistosomiasis
Source: PLOS Glob Public Health. 2025 Dec 8;5(12):e0004235. doi: 10.1371/journal.pgph.0004235 (PMC12685162; doi:10.1371/journal.pgph.0004235)
Supplement: S1 Text — (PDF) [file pgph.0004235.s001.pdf]

S1 Text

Additional Training Information
2023 FGS Peer-to-Peer Virtual Training Events

In 2021, the Virtual Training Course (Phase 1) for healthcare professionals on integrating FGS into their practice was piloted for Anglophone and Francophone Africa[1]. There were 1,527 applications, 484 health professionals were accepted, and over 300 participants completed the course. The selection process aimed to ensure an equal distribution of genders and achieve a balance between participants from health facilities (e.g., clinicians) and those from non-health facilities (e.g., community health workers). Participants who met these criteria were chosen on a first-come, first-served basis. Those who were unavailable during the scheduled program time or unable to connect were not selected. Individuals who were not selected were encouraged to engage with the program by watching recorded sessions or participating in future programming. The Impact Accelerator (Phase 2) emerged from the need for real-time feedback and support, allowing the team to better understand participant challenges and provide timely assistance. Participants of the 2021 course self-reported training an additional 2,052 healthcare professionals, treating 3,892 girls and women, and sharing basic information about FGS (e.g., prevention methods, symptoms, treatment options)among 120,666 community members and leaders who had little or no prior knowledge of the disease.. These successes demonstrated the potential of the training approach to address this neglected disease. In 2023, the END Fund sponsored another round of the training program (Phases 1 and 2) for participants from Francophone Africa.

All 2023 FGS Peer-to-Peer Virtual Training events in Phases 1 and 2 were facilitated by TGLF with support from Bridges to Development staff. Subject matter experts (SMEs) presented the core FGS concepts and served as guides. Events maximized opportunities for participants to learn from and with one another. Participants' offline work on their action plans and direct peer-to-peer communications complemented the events. Phases 1 and 2 are described below, followed by links to additional information on the training and learner resources.

Phase 1: Virtual Training Course

During Phase 1, participants learned FGS core competencies and wrote an action plan to improve FGS outcomes in their communities. Phase 1 began with two general assemblies, during which participants had an opportunity to (1) learn about FGS, (2) discuss FGS scenarios and real-life cases, and (3) receive information on and resources for developing action plans. An optional 30-minute remote coffee was provided to all participants using the same link to socialize, ask questions, and network before and after the assemblies. Subsequently, five peer support sessions were held to strengthen action plan development and for peer review. Action plans were developed and reviewed by participants and SMEs on an online platform using a provided rubric. Guidance for developing and evaluating action plans focused on feasibility, adherence to national guidelines, integration with other health programs, and capacity for community engagement. The course concluded with a third general assembly to (1) review group progress and action plan development, (2) discuss learning related to the peer-review process, (3) hear SME feedback, and (4) learn about ways to stay connected. Details on each event follow.

[1]Francophone countries included: Benin, Burkina Faso, Cameroon, Congo, Ivory Coast, Gabon, Guinea, Madagascar, Mali, Morocco, Niger, Democratic Republic of the Congo, Senegal, Chad, and Togo. Anglophone countries included: Cameroon, Ghana, Kenya, Liberia, Madagascar, Malawi, Nigeria, and United Arab Emirates, Zambia.

S1 Text

General Assembly One <u>(Required)</u> | May 4, 2023 (~3 hours)
(*Remote coffee*)→Welcome and introduction→Remote coffee [1:1]→FGS presentation and Q&A→Peer-learning exercise: Scenario-based group discussions→Reflections and Announcements→(*Remote coffee)*

The first general assembly began with a ~10-minute welcome and an introduction by event facilitators, during which SMEs shared how they first learned about FGS and why it is of concern to them. The assembly then continued with a ~5-minute "remote coffee" for participants to meet with one other participant in breakout rooms, following which SMEs presented on FGS and answered participants' questions posed aloud or in the chat for ~30 minutes. A brief overview of the scenario-based group discussion was then provided to all participants by the event facilitators, and over the next two hours, small-group discussions were held to review the two scenarios. Each small-group discussion began with participants reviewing the scenario details together and discussing how best to address them in breakout rooms for ~30 minutes. They then returned to discuss their thoughts as a whole group for another ~30 minutes. During whole group discussions, SMEs contributed by making FGS core competency connections to small-group work reports and posing additional questions or ideas for consideration. The first assembly concluded with ~5 minutes of reflection and announcements by event facilitators and SMEs.

General Assembly Two <u>(Required)</u> | May 9, 2023 (~3 hours)
(*Remote coffee*)→Welcome and Review→FGS action plan information→Peer-learning exercise: Group discussions on real-life FGS challenges→Reflections and Announcements→(*Remote coffee)*

The second general assembly began with a ~15-minute welcome and review of the schedule by event facilitators and SMEs, after which they dedicated ~20 minutes to sharing about the FGS action plan development process and criteria. Following the first general assembly format for collaborative brainstorming, participants then held small and whole-group discussions for two hours on the real-life FGS challenges submitted by participants who agreed to have their cases reviewed for additional support. The assembly concluded with ~15 minutes of reflection and announcements by event facilitators and SMEs.

Peer-Support Sessions <u>(Optional)</u> | 5 sessions, May 10-16, 2023 (~1 hour)
Welcome→Review of information→Discussion and Q&A

Peer support sessions began with a brief welcome and a review of the selected topic by event facilitators. Selected topics for the first three sessions were about action planning (how to draw up, complete, submit, and get started), and the last two sessions were about the peer review process (how to get started and submit to journals). After reviewing the information presented on the selected topic, an open group discussion was held, allowing participants to discuss their connections to the topic and ask questions. The sessions then focused on listening to one participant share their FGS challenge and the actions they would take to solve it, with feedback and comments from peers and SMEs. During some sessions, participants also discussed what they were learning from the peer review process and how they would apply that knowledge to

improve their action plans. During all sessions, technical support was provided to participants who had difficulties using the peer review platform.

General Assembly Three <u>(Required)</u> | May 17, 2023 (~2 hours)
(*Remote coffee*)→Welcome, group progress review, & recap→Participant presentations and feedback from SMEs→Closing ceremony and Announcements→(*Remote coffee)*

The third general assembly began with a ~20-minute welcome, a review of the whole group's action plan development, and a recap of important points from the second general assembly by SMEs and event facilitators. Then, over the next ~80 minutes, participants presented their action plans and discussed what they learned from the peer-review process with the whole group. SMEs provided feedback to participants to further strengthen action plans. The assembly concluded with a ~20-minute closing ceremony and final announcements. During the closing ceremony, participants, SMEs, and event facilitators celebrated the group's progress over Phase 1. Final announcements included an invitation to join Phase 2, the Impact Accelerator, and other FGS, Bridges to Development, and TGLF groups and programs like the Genital Schisto Community of Practice, 24 country-based communities of TGLF scholars, and Teach to Reach: Connect events.

**Peergrade, an online platform, was used to submit and review action plans. On May 11, 2023, participants submitted their draft action plans for peer review. On May 16, 2023, participants submitted their reviews of three peers' action plans. On May 19, 2023, participants submitted their own revised and improved action plans.*

Phase 2: Impact Accelerator

During Phase 2, participants received additional support for implementing their action plans through general assemblies, a remote coffee, and lightning chats. Participants also worked individually to implement their plans and communicated with their peers outside of events. The first two weeks of Phase 2 were considered the Launchpad of the Impact Accelerator. During the two general assemblies of the Launchpad, participants (1) reported on their action plan implementation progress, (2) set a specific goal to accomplish by the end of the two weeks, shared stories of successful and challenging experiences, and (4) reflected on lessons learned. Participants received and shared recommendations and resources from each other and SMEs. Following the Launchpad, participants were encouraged to participate in a remote coffee during their own time. Two lightning chats followed, featuring specific participants sharing their experiences of implementing their action plans. The successes and challenges they presented facilitated discussion and future planning among all participants. Details on each event follow.

Launchpad: General Assembly One <u>(Required)</u> | November 6, 2023 (~1 hour)
Welcome & overview→Peer-sharing & Goal setting→SME feedback→Closing announcements

The first general assembly began with a ~15-minute welcome to participants and an overview of Phase 2 by event facilitators. The overview included information on the Impact Accelerator schedule, group demographics, and concluded by asking participants for their commitment. Participants pledged to form an active part of the human knowledge-sharing network, working with others, sharing information, and regularly reporting on progress to transform knowledge

into action and improve global health outcomes, particularly those related to FGS. Then, facilitators briefly shared statistics of the group's progress made to date. For the following ~15 minutes, participants shared their stories of successful implementation, lessons learned through challenges presented, and existing barriers to implementation. Over the next ~30 minutes, participants set goals they could accomplish within two weeks and discussed them. Afterward, the event facilitators provided instructions/tips on how to report their goals online. Continuing, SMEs provided feedback on what was shared for ~15 minutes and advice on goal setting. The assembly concluded with facilitators reminding participants of the next steps.

Launchpad: General Assembly Two (Required) | November 13, 2023 (~1 hour)
Welcome & review→Peer-sharing→SME feedback→Goal setting→SME feedback→Closing announcements

The second general assembly began with ~10 minutes of a welcome, a reintroduction of the pledge made by all participants, a brief overview of the Phase 2 schedule, and a review of the first week's progress by event facilitators. For the next ~30 minutes, participants spoke about their first week–how it went for them, what they learned, what hindered their progress, what surprised them, and what their next steps would be. Over the next ~10 minutes, SMEs were asked to follow up directly to participants' questions and then provide more generalized feedback to the whole group on how to advance despite the challenging situations raised. Next, event facilitators invited participants to consider what they would like to achieve by the end of the week, as well as by December 15th. Participants shared for ~10 minutes what their upcoming goals were and the actions they would take to achieve them. The general assembly concluded with ~10 minutes of final SME feedback and reminders of upcoming events.

Independent Remote Coffee (Encouraged) | Two conversations (~15 to 30 minutes each)
For this remote coffee, participants were randomly matched with another participant they were to contact within a week via their preferred communication methods (e.g., Zoom, WhatsApp, Messenger). This meant that participants had the opportunity to speak with two scholars, one who contacted them and one whom they contacted. Although participants' conversations were not restricted during the remote coffee, they were encouraged to keep conversations social and limited to ~15 to 30 minutes.

Lightning Sessions (Optional) | November 15, 2023 & November 22, 2023 (~1 hour each)
Welcome & review→Speaker testimony

Both lightning sessions followed a similar format. They began with a ~5-minute welcome, review (i.e., of the reality of FGS, information on training event sponsorship and structure), and an introduction to the selected participant speaker by an event facilitator. Then, the speakers spoke for ~5 minutes about FGS in their communities and described how they had worked during the Launchpad to accomplish the goals they set. For the next ~35 minutes, the speakers mainly answered participants' questions, with some feedback and questions posed by SMEs. The lightning chats concluded with a ~5-minute wrap-up, thank you to everyone involved in the fight against FGS, and announcements about future events.

S1 Text

2023 FGS Training Event Resources and Recordings	
2023 FGS training event webpage	https://www.learning.foundation/fgs-fr
FGS Telegram group	https://t.me/+Y43li8lyAdNlN2Q0
Session recordings	https://youtube.com/playlist?list=PLti7k0eaN3gRvrPdYVA1gEoyyX10Xkn6c&feature=shared
FGS core competencies	https://reproductive-health-journal.biomedcentral.com/articles/10.1186/s12978-021-01252-2
Learner resources	https://www.dropbox.com/sh/0oze7fv4u6vifpo/AAAPKr5ihUJaM4Bi_0oo-peea?e=1&dl=0
PeerGrade (online platform used for peer and SME review)	https://www.peergrade.io/
FGS FAQ document	https://zenodo.org/records/8415126
Minimum Service Package (MSP) for FGS Integration<br><br>*During the training, participants informally discussed FGS-related stigma in their contexts. It was not an official program topic but led to additional work. After the training, Bridges to Development partnered with other organizations to develop and pilot a MSP for FGS integration into SRHR services in Kenya. The MSP has service delivery points across all levels and incorporates training on stigma, especially at the community level, as part of its health literacy, social inclusion, and equity components.	https://www.eliminateschisto.org/sites/gsa/files/content/attachments/2024-07-08/2023-09-05%20FINAL%20MSP%20-%20Appendix%20A%20ENG%20-clean%2026.6.pdf
