# Supplementary material for: Decolonizing infectious disease programs: A mixed methods analysis of a novel multi-country virtual training for Female Genital Schistosomiasis
Source: PLOS Glob Public Health. 2025 Dec 8;5(12):e0004235. doi: 10.1371/journal.pgph.0004235 (PMC12685162; doi:10.1371/journal.pgph.0004235)
Supplement: S2 Text — (PDF) [file pgph.0004235.s003.pdf]

S2 Text
Partner Media Connections

Bridges to Development	Partner Media Connections	TGLF Connections
https://bridgestodevelopment.org/	Website	https://www.learning.foundation/
https://x.com/bridges2develop	Twitter / X	https://x.com/DigitalScholarX
https://www.instagram.com/bridgestodevelopment/	Instagram	https://www.instagram.com/thegenevalearningfoundation/
https://www.linkedin.com/company/bridgestodevelopment/	LinkedIn	https://www.linkedin.com/company/geneva-learning-foundation/mycompany/
	Facebook	https://www.facebook.com/DigitalScholar
	Telegram	https://t.me/GenevaLearning
	Podcast	https://www.learning.foundation/podcast
	YouTube	https://www.youtube.com/@TheGenevaLearningFoundation
https://zenodo.org/communities/bridgestodevelopment/	Zenodo	https://zenodo.org/communities/tglf/records?q=&l=list&p=1&s=10&sort=newest
