# Supplementary material for: Decolonizing infectious disease programs: A mixed methods analysis of a novel multi-country virtual training for Female Genital Schistosomiasis
Source: PLOS Glob Public Health. 2025 Dec 8;5(12):e0004235. doi: 10.1371/journal.pgph.0004235 (PMC12685162; doi:10.1371/journal.pgph.0004235)
Supplement: S3 Text — (PDF) [file pgph.0004235.s004.pdf]

Fig A: Phase 1 Action Plan Program Integration

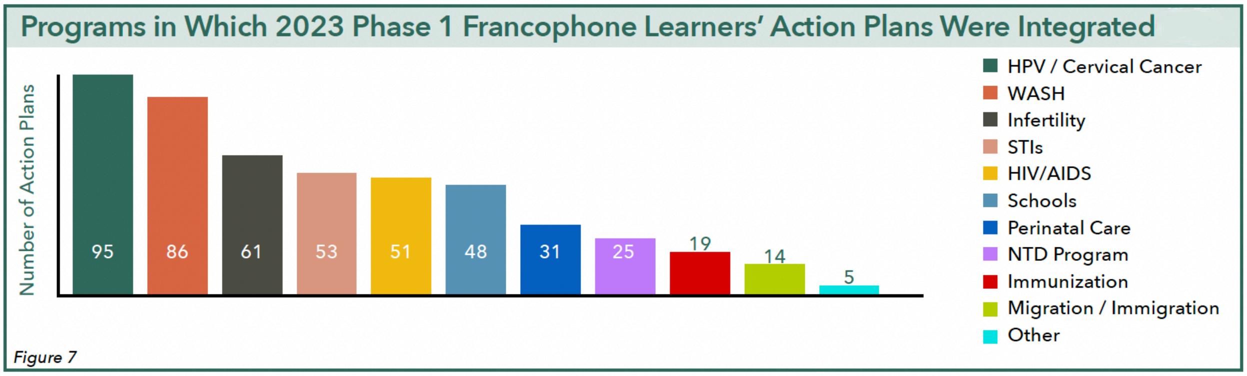

Note: The term "other" encompasses programs that are not specifically listed in Figure 3 but into which participants integrated their action plans (e.g., the cholera program).

Fig B: Phase 2 Action Plan Objectives

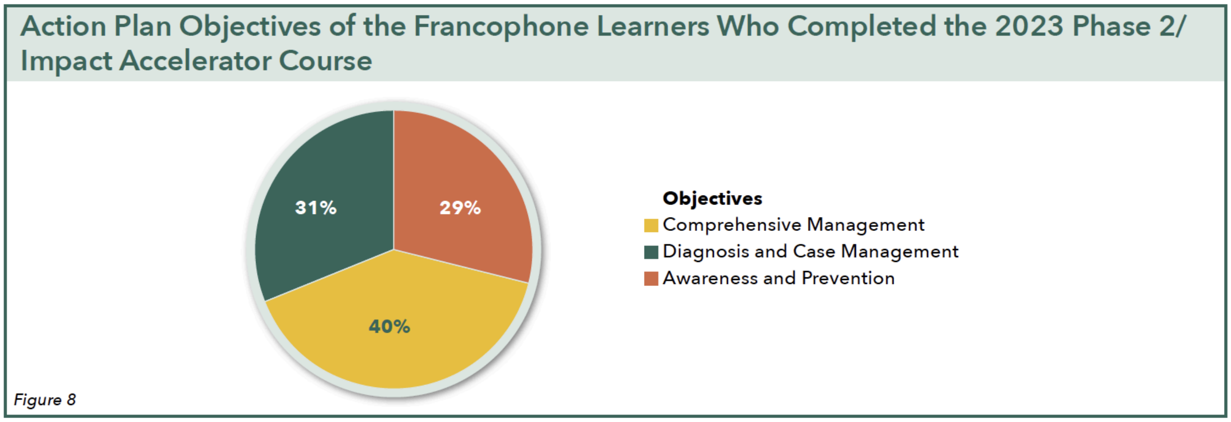

Fig C: Phase 2 Distribution of Action Plan Objectives

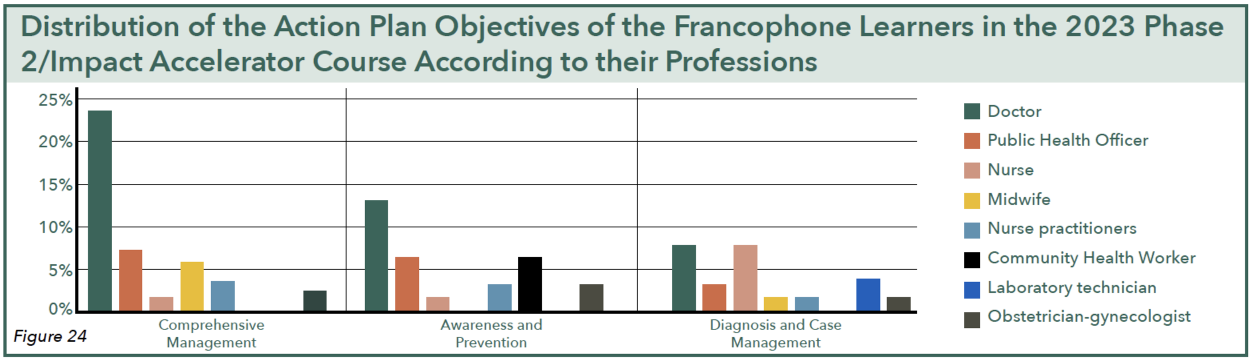

Fig D: Phase 2 Interactions Between Course Applicants

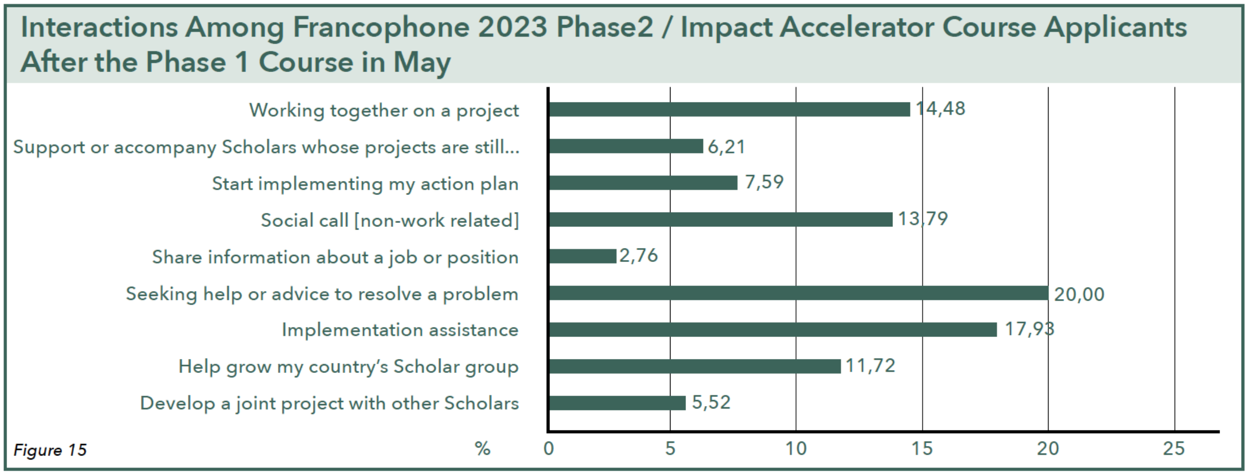

Table A: Qualitative Codes and Categories

Codes	Categories
Unavailability of personnel	Implementation Challenges
Insufficient time	
Missing materials, medicine, and data	
Waiting on or coordinating a response	
Securing funding	
Site safety and stability	
Addressing reluctance	
Relocation challenges, travel disruption, and distance to the work site	
Action plan integration into local and district activities	Implementation Strategies
Action plan integration into national activities	
Advocacy with key health leaders	
Collaboration with health workers and community leaders	
Petitioning for financial, material, data, and medical support	
Utilization of audio and digital communication strategies	
Learning new information and techniques	
Utilization of global guidance	
Sharing advice and ideas with peers	
Receiving advice and support from experts and mentors	
Phasing financing and activities	
Outputs	Implementation Results
Outcomes	

S3 Text

Table B: Socio-demographic Characteristics of Accepted Participants

Category	Demographic	Phase 1 (N=786)	Phase 2 (N= 145)
**Gender**	Women	386 (49.3%)	51 (35.4%)
	Men	397 (50.7%)	93 (64.6%)
**Age Range (in Years)**	18–35	286 (36.4%)	41 (51.9%)
	35 and above	500 (63.6%)	103 (48.1%)
**Region**	Central/Middle Africa<br>Cameroon, Central African Republic, Congo, Democratic Republic of the Congo, Equatorial Guinea, Gabon	383 (48.7%)	68 (46.9%)
	Northern Africa<br>Chad, Mauritania, Morocco, Sudan, Tunisia	32 (4.1%)	0 (0.0%)
	Western Africa<br>Benin, Burkina Faso, Guinea, Ivory Coast, Mali, Niger, Nigeria, Senegal, Togo	316 (40.2%)	46.2%
	Southern Africa<br>Angola, Namibia, South Africa, Swaziland (Eswatini)	5 (0.6%)	1 (0.7%)
	Eastern Africa<br>Burundi, Comoros, Djibouti, Ethiopia, Madagascar	43 (5.5%)	9 (6.2%)
	Others	8 (1.0%)	0 (0.0%)
**Profession**	Doctor/Obstetrician-gynaecologist	340 (43.4%)	69 (47.9%)
	Public Health Officer*	171 (21.8%)	23 (16.0%)
	Nurse/Midwife/Nurse practitioner	167 (21.2%)	28 (19.4%)
	Community health worker	63 (8.0%)	14 (9.7%)
	Laboratory technician	44 (5.6%)	10 (6.9%)
**Organization**	Public hospital/health facility	181 (23.1%)	30 (21.4%)
	Ministry of Health staff at national level	159 (20.3%)	32 (22.9%)
	Private organization**	162 (20.8%)	25 (17.9%)
	Ministry of Health staff at subnational level	122 (15.5%)	20 (14.3%)
	University/academic institution	94 (12.0%)	20 (14.3%)
	Private hospital/health facility	66 (8.4%)	13 (9.3%)

Table C: Regression Analysis of Factors Influencing Course Completion

	Phase 1		Phase 2	
**Parameters**	**Odds Ratio (Confidence Interval)**	**P-value**	**Coefficient**	**Credible Interval (2.5%, 97.5%)**
**Gender**				
Male	1		1	
Female	0.42 (0.22, 0.79)	0.008*	-0.11	-0.99, 0.75
**Profession**				
Doctor/Obstetrician-gynaecologist	1		1	
Nurse/Midwife/Nurse practitioner	2.86 (0.69, 11.91)	0.149	0.80	-0.27, 1.92
Laboratory technician	1.84 (0.35, 9.77)	0.472	-0.94	-2.76, 0.62
Public health officer	1.32 (0.35, 4.96)	0.679	-0.29	-1.52, 0.97
Community health worker	0.57 (0.16, 2.06)	0.390	-1.75	-3.58, -0.10*
**Organization**				
Public hospital/health facility	1		1	
Private hospital/health facility	1.58 (0.52, 4.79)	0.422	0.93	-0.65, 2.62
University/academic institution	2.03 (0.62, 6.62)	0.243	-1.02	-2.59, 0.42
Ministry of Health staff at national level	1.03 (0.43, 2.47)	0.955	-0.48	-1.71, 0.74
Ministry of Health staff at subnational level	0.96 (0.34, 2.69)	0.938	-0.23	-1.69, 1.24
Private organization	1.37 (0.49, 3.84)	0.552	0.17	-1.31, 1.72
**Perform pelvic exams as part of routine work responsibilities**				
No	1		1	
Yes	0.63 (0.3, 1.33)	0.226	-0.63	-1.63, 0.37
**Coverage of costs incurred**				
Neither I nor my employer covered any cost incurred.	1		-	-
I paid out of pocket for my expenses.	2.45 (1.26, 4.78)	0.008*	-	-
My employer covered all my expenses.	1.08 (0.07, 16.3)	0.956	-	-
My employer and I covered part of the costs.	3.99 (0.44, 36.51)	0.220	-	-
**Had prior experience with the management of female genital schistosomiasis**				
No	1		-	-
Yes	0.78 (0.42, 1.46)	0.439	-	-
**Difficulties encountered during the workshop**				
Had difficulties with concepts or training materials.	1		-	-

S3 Text

	Phase 1		Phase 2	
**Parameters**	**Odds Ratio (Confidence Interval)**	**P-value**	**Coefficient**	**Credible Interval (2.5%, 97.5%)**
I didn't find anything difficult for me.	3.45 (1.15, 10.34)	0.027 *	-	-
Training technology was difficult.	2.35 (0.72, 7.72)	0.158	-	-
**Workshop certificate will be recognized by my employer**				
No	1		-	-
Yes	1.61 (0.58, 4.43)	0.359	-	-
**Prior online learning experience**				
False	1		-	-
True	1.96 (0.69, 5.54)	0.204	-	-
**First peer-to-peer learning experience**				
False	1		-	-
True	1.56 (0.8, 3.05)	0.196	-	-
**Action plan implementation status before Impact Accelerator**				
I have not started the implementation of my action plan.	-	-	1	-
The implementation of my action plan is in progress.	-	-	1.19	0.13, 2.35*
The implementation of my action plan is already complete.	-	-	0.64	-1.29, 2.66
**The objectives of my action plan are part of my job responsibilities**				
False	-	-	-	-
True	-	-	-0.15	-1.17, 0.85
**The objectives of my action plan have been integrated into an existing program or initiative**				
False	-	-	-	-
True	-	-	0.03	-0.96, 1.03
**Confidence level for implementing action plan before Impact Accelerator**				
Confident	-	-	-	-
Not confident	-	-	0.65	-0.34, 1.71
Very confident	-	-	0.80	-0.33, 1.97

S3 Text

Table D: Phase 1 and Phase 2 Outcomes on Knowledge, Confidence, and Action Plan Implementation (N=294*)

A. Knowledge and Confidence		
	**Pre-Training**	**Post-Training**
**Awareness of FGS**		
No awareness/knowledge	13 (4.4%)	3 (1.0%)
Minimal awareness	46 (15.6%)	8 (2.7%)
Basic knowledge	69 (23.5%)	13 (4.4%)
Moderate knowledge	75 (25.5%)	25 (8.5%)
Substantial knowledge	38 (12.9%)	106 (36.2%)
Comprehensive knowledge	53 (18.0%)	138 (47.2%)
**Knowledge to diagnose FGS**		
No	176 (59.9%)	28 (9.5%)
Yes	118 (40.1%)	266 (90.5%)
**Knowledge to treat FGS**		
No	185 (62.9%)	40 (13.61%)
Yes	109 (37.1%)	254 (86.39%)
**Knowledge to prevent FGS**		
No	125 (42.52%)	10 (3.40%)
Yes	169 (57.48%)	284 (96.60%)
**Confidence to discuss FGS with patients**		
No confidence	13 (4.4%)	4 (1.4%)
Very low confidence	51 (17.3%)	5 (1.7%)
Low confidence	50 (17.0%)	14 (4.8%)
Moderate confidence	67 (22.8%)	21 (7.2%)
High confidence	55 (18.7%)	85 (29.0%)
Full confidence	58 (19.8%)	164 (55.9%)
**Individual Increases of ≥2 Levels in Knowledge and Confidence**		
Knowledge/diagnosis	-	196 (66%)
Confidence in discussing FGS	-	197 (66%)
**B. Action Plan**		
**Begun Implementation**		
After Phase 1	89 (39.0%)	
Before Phase 2	89 (62.0%)	
After Phase 2	52 (71.0%)	
**Completed Implementation**		

After Phase 1	4 (1.0%)	
Before Phase 2	9 (6%)	
After Phase 2	16 (23%)	

Note: Number of people who turned in complete responses in the post-training survey.

Table E: Reach Achieved After Phase 2

**Activity**	**Number Reached**	**% of Participants Involved**
Healthcare workers trained by participants	2,675	91%
Patients diagnosed/managed	638	39%
Community members taught about FGS	49,088	82%

Table F: Logistics Regression of Factors Influencing Learning Gains

Parameter	Odds ratio (Confidence Interval)	P-value
**Gender**		
Male	1	
Female	1.18 (0.6, 2.34)	0.628
**Profession**		
Doctor/Obstetrician-gynaecologist	1	
Nurse/Midwife/Nurse practitioner	1.11 (0.45, 2.77)	0.819
Laboratory technician	0.64 (0.19, 2.22)	0.486
Public health officer	0.54 (0.22, 1.36)	0.193
Community health worker	0.17 (0.04, 0.67)	0.012*
**Perform pelvic exams as part of routine work responsibilities**		
False	1	
True	0.81 (0.37, 1.77)	0.598
**Organization**		
Public hospital/health facility	1	
Private hospital/health facility	1.18 (0.29, 4.8)	0.816
University/academic institution	0.32 (0.1, 1.06)	0.061
Ministry of Health staff at national level	0.35 (0.12, 1.02)	0.055
Ministry of Health staff at subnational level	0.39 (0.12, 1.25)	0.114
Private organization	0.67 (0.2, 2.21)	0.506
**Had prior experience with the management of female genital schistosomiasis**		
False	1	

Parameter	Odds ratio (Confidence Interval)	P-value
True	0.17 (0.04, 0.71)	0.015*
**Difficulties encountered during the workshop**		
Had difficulties with concepts or training materials.	1	
I didn't find anything difficult for me.	0.6 (0.14, 2.6)	0.494
Training technology was difficult.	0.77 (0.15, 3.83)	0.748
**Workshop certificate will be recognized by my employer**		
False	1	
True	0.16 (0.02, 1.38)	0.097
**Work Environment**		
Rural	1	
Urban	1.2 (0.63, 2.29)	0.576

Table G: Peer-to-peer Learning Model Regression (Influence of model on knowledge acquisition/change in knowledge/diagnosis levels)

**Parameters**	**Coefficient**	**Credible Interval (2.5%, 97.5%)**
**First peer-to-peer learning experience**		
False		
True	-0.10	-0.65, 0.43
**Impact of peer review on knowledge acquisition**		
I did not learn.		
I learned more than I expected	-0.45	-1.83, 0.83
I learned less than I expected	-0.48	-2.09, 0.98
I learned what I expected	-0.77	-2.20, 0.61
**Peer support was useful**		
False		
True	1.97	0.80, 3.30*
**Impact of peer reviewing the work of colleagues**		
I did not do it		
Not useful	1.65	-1.12, 5.32
Useful	0.03	-1.04, 1.08
Very useful	0.16	-0.86, 1.14

Table H: Knowledge Gains and Expanded Reach

Quantitative Results	Qualitative Results	Mixed methods meta-inferences
**Increased Knowledge and Confidence:** 66% of Phase 1 participants reported an increase in their FGS knowledge and diagnosis capacities and confidence in discussing FGS.<br><br>However, community health workers had 83% lower odds of achieving increased knowledge levels compared to doctors/obstetrician-gynecologists. Similarly, participants with prior experience managing female genital schistosomiasis had 83% lower odds of reporting such gains, compared to those who did not.	**Neural Connections–FGS Technical Knowledge and Skills Learned:** Phase 1 provided new knowledge, reinforced existing understanding and confidence, and addressed misinformation.<br><br>"*I have almost ten years of professional experience in the fight against NTDs, and it was only during this training that I learned the concept of FGS.*"<br>– Public Health Officer working in a Ministry of Health<br><br>**Social/External Connections–Diverse Connections and Resources for Growth and Impacts:** Participants gained higher responsibilities, more patients, and greater community trust.<br><br>*"I am confident in what I am doing, and my colleagues have confidence in me when it comes to bilharzia compared to before."* – Public Health Officer working in a private hospital/health facility	◊ ***Complementarity*****:** Increases in knowledge and confidence, alongside disparities by role and experience, are complemented by accounts showing how learning corrected misinformation, built credibility, and strengthened professional trust.
Broad Impact and Amplified Reach: After Phase 2, 39% of participants diagnosed or managed patients with FGS, amounting to approximately 638 cases. 91% of participants reported training a total of 2,675 colleagues. Additionally, 82% of participants engaged with their communities, reaching over 49,000 individuals with information about FGS.	**Neural Connections–FGS Technical Knowledge and Skills Learned:** The knowledge and confidence gained enabled participants to share information with colleagues, while the skills acquired supported the identification and diagnosis of FGS.<br><br>"*I have raised awareness on social networks. I even started to stimulate the (reflections) of my colleagues as well as the midwives and obstetrician nurses on the questions of FGS in the different communication channels like WhatsApp, Telegram, and on Facebook.*" – Doctor working in a	◊ ***Confirmation*****:** Patients reached, colleagues trained, and communities engaged are confirmed by accounts describing knowledge sharing and professional credibility.<br><br>▲ ***Expansion:*** The broad reach of Phase 2 is expanded by qualitative accounts showing how confidence and skills were transformed into advocacy, knowledge sharing, and wider influence.

Quantitative Results	Qualitative Results	Mixed methods meta-inferences
	private hospital/health facility<br><br>**Social/External Connections–Diverse Connections and Resources for Growth and Impacts:** Fostered confidence, motivating participants to effect change and share knowledge through community engagement and case documentation.<br><br>"*This training allowed me to understand that I am now a citizen of the world and that my single advice to a colleague can be useful to them in addressing a public health problem or saving a life on the other side of the world.*" – Public health officer working at a non-profit	

Table I: Action Plan Development and Implementation

Quantitative Results	Qualitative Results	Mixed methods meta-inferences
**Diverse Action Plans Aligned with Professional Responsibilities:** Three main categories of action plans emerged in Phase 2: awareness and prevention, diagnosis and case management, and comprehensive management. Doctors accounted for most comprehensive plans, community health workers focused on awareness, and laboratory technicians on diagnosis. Pelvic exams were reported by 60% with diagnosis objectives, 68.4% with comprehensive objectives, and 35.9% with awareness objectives.	**Theme 2–Connectivistic Connections Despite Complexities:** Reasons for not completing action plans included awaiting data, funding, or authorization, adjusting timelines for personal or community activities, planning multi-region projects that required more time, and a lack of available FGS medication.	◊ ***Complementarity*:** The distribution of action plan types and their alignment with professional responsibilities are complemented by accounts highlighting the practical barriers that shaped implementation, including resource gaps, authorization delays, and medication shortages.

S3 Text

Quantitative Results	Qualitative Results	Mixed methods meta-inferences
**Integrated Action Plans:** 82% of action plans developed during Phase 1 included strategies for integration with other programs, such as HPV/cervical cancer, WASH, infertility, STIs, HIV/AIDS, schools, perinatal care, NTD programs, immunization, and migration/immigration.	**Theme 1–Accelerating Connections and Integration:** Integration increased the likelihood of action plan implementation by enabling advocacy, fostering collaboration, and leveraging existing services and programs.	◊ ***Confirmation***: Integration was the predominant strategy across action plans and was consistently emphasized as central to implementation.<br><br>▲ ***Complementarity:*** Integration was further explained through accounts showing how it enabled advocacy, fostered collaboration, and leveraged existing services and programs.
**Increased Capacity for Action Plan Implementation:** By the end of Phase 1, 39% had begun and 1% had completed their action plans. After Phase 2, 71% had begun and 23% had completed implementation.	**Conceptual Connections–Peer Learning and Networking to Strengthen Action Planning:** Development and implementation of action plans were strengthened by peer review, networking, and continued engagement after Phase 1. Barriers such as lack of funding, medicines, and health authority buy-in were addressed through advocacy, peer support, and expert consultation.<br><br>"*When I was unable to deploy my action plan due to lack of financial means, I seemed disoriented, until by following live testimonies and reading via Telegram, (I learned) the methods used by other scholars who did not have the financial means like me, to reach populations at risk. This knowledge made me change my approach. It is clear that when we participate in such a launchpad (Phase 2)...we (are) influenced by new ideas, (and) other scholars from diverse backgrounds*." – Community health worker working at the district level	◊ ***Complementarity***: Quantitative findings demonstrated increased capacity for action plan implementation after Phase 2, while qualitative findings complemented this by explaining the processes that enabled implementation, including peer learning, networking, and advocacy to overcome barriers.

Table J: Impact of Peer Learning-to-Action Model

Quantitative Results	Qualitative Results	Mixed methods meta-inferences
**Peer Support Influenced Knowledge Gains:** *Those who reported peer support as useful had higher log-odds of having an increase in knowledge levels compared to those who did not report peer support as useful.*	**Social/External Connections–Diverse Connections and Resources for Growth and Impacts:** Peer interactions fostered confidence, deepened interest in FGS, improved listening skills, and supported critical thinking on cross-border collaboration.<br><br>"*Since I am not a medical professional…talk(ing) about the profession…with colleagues from other countries added more to my knowledge (and)...I strengthened my abilities in health.*"– Community Health Worker of a non-governmental organization	◊ ***Confirmation*****:** Peer support was identified as a driver of knowledge gains and was confirmed by accounts describing confidence, deeper interest, and strengthened abilities through interaction with colleagues.<br><br>▲ ***Complementarity*****:** Findings on peer support were complemented by accounts showing additional benefits, including improved listening skills, critical thinking, and cross-border collaboration.
**Impact of Peer Interactions:** Among Phase 1 participants, 61% reported in the post-training survey that they learned more than they had anticipated from the peer reviews. 85% indicated that they experienced significant changes in their professional practice as a result of participation.	**Social/External Connections–Diverse Connections and Resources for Growth and Impacts:** The program made participants more proactive, improved work performance, and expanded networks that provided support, quick access to information, collaboration, and stronger advocacy to health authorities.<br><br>"*My participation changed me as a professional. Indeed, I realized that several neglected tropical pathologies, notably FGS, are, in reality, public health problems, and not thinking about them worsens the state of health. However, if we should take it into account from the start this would avoid patients to spend a lot of money on their health conditions.*" – Doctor working in a private hospital/health facility	◊ ***Confirmation*****:** Peer reviews enhanced learning and professional practice, confirmed by accounts of greater proactivity, improved performance, and stronger advocacy.<br><br>▲ ***Complementarity*****:** Reported changes in practice were complemented by accounts showing how peer interactions expanded networks, fostered collaboration, and reshaped views of FGS as a pressing public health issue.
**Influence of Model on Action Plan Development and Implementation:** 86% stated that the peer reviews significantly improved their own action plans, while 85% found reviewing their colleagues' action plans to be	**Conceptual Connections–Peer Learning and Networking to Strengthen Action Planning:** Peer support was widely valued, with many finding it more beneficial than materials. Formal and informal peer interactions improved action	◊ ***Confirmation*****:** Peer reviews were shown to improve action plans, confirmed by accounts describing how feedback directly strengthened plan content and clarity.<br><br>▲ ***Complementarity*****:** The value of

Quantitative Results	Qualitative Results	Mixed methods meta-inferences
beneficial.	plan development, clarified expectations, and supported implementation through ongoing engagement and shared experiences.<br><br>"*The peer review allowed me to improve my action plan. For example, a peer reminded me that the title of my plan had to start with an action verb. Another suggested that I find a better map of the health districts…because the one I had included in the initial action plan was not too expressive. These two examples, to name just a few, demonstrate the extent to which peer review remains essential*." – Public health officer at a non-profit	peer support was complemented by accounts illustrating how both formal and informal interactions provided practical guidance, fostered learning, and sustained implementation.
**Continued Engagement Post-Training:** A substantial proportion of participants (77.2%) contacted their peers after Phase 1 for collaborative purposes. 61% of these participants also credited maintaining contact with their peers after the program to the peer support model.	**Social/External Connections–Diverse Connections and Resources for Growth and Impacts:** Peer-driven networks within and across countries provided support, quick access to information, collaboration, and collective advocacy that helped overcome implementation challenges and achieve goals.<br><br>"*There is a whole network of global practitioners around me, although virtual, but I know that I can be helped at any time*." – Doctor in the Ministry of Health	◊ ***Confirmation*:** Post-training engagement was confirmed by accounts describing ongoing peer networks that provided collaboration and support.<br><br>▲ ***Complementarity*:** These findings were complemented by accounts showing how networks offered rapid access to information, cross-country connections, and collective advocacy.
