# Supplementary material for: Decolonizing infectious disease programs: A mixed methods analysis of a novel multi-country virtual training for Female Genital Schistosomiasis
Source: PLOS Glob Public Health. 2025 Dec 8;5(12):e0004235. doi: 10.1371/journal.pgph.0004235 (PMC12685162; doi:10.1371/journal.pgph.0004235)
Supplement: S4 Text — (PDF) [file pgph.0004235.s005.pdf]

Texte S4
Manuscrit en français

Décolonisation des programmes de lutte contre les maladies infectieuses : Une analyse de méthodes mixtes d'un nouveau formation virtuelle multi-pays sur la bilharziose génitale féminine

Note de l'auteur

Kari Eller[1,3*], Kelechi Amy Nwoku[2], Reda Sadki[3], Nicole Vecchio[2], Caroline Pensotti[2], Charlotte Njua Mbuh[3], Julie Jacobson[2]

[1]Département de l'éducation permanente, de l'administration et des politiques, Université de Géorgie, Athéna, États-Unis d'Amérique

[2]Bridges to Development, Vashon, États-Unis d'Amérique

[3]La Fondation Apprendre Genève, Genève, Suisse

[*]Auteur correspondant
Courriel : kari.eller@uga.edu

Texte S4
Manuscrit en français

Résumé

Les programmes d'enseignement médical existants et le développement professionnel continu en matière de maladies infectieuses sont largement basés sur des supports provenant de pays à revenu élevé et excluent l'enseignement sur la bilharziose génitale féminine (BGF).
En 2023, le modèle d'apprentissage par les pairs de la Fondation Apprendre Genève a été utilisé pour créer un programme de formation virtuel inclusif et multinational afin de combler cette lacune et de renforcer les capacités des professionnels de santé locaux dans les pays endémiques. Au cours de la phase 1 du programme, les participants ont appris ce qu'est la BGF et ont élaboré un plan d'action local pour y remédier. Au cours de la phase 2, les participants ont reçu un soutien pour la mise en œuvre de leurs plans d'action. Afin d'explorer cette approche pédagogique et sa capacité à toucher un public diversifié de professionnels de la santé, nous avons mené une étude à méthodes mixtes s'inscrivant dans le cadre théorique du connectivisme. Des données quantitatives et qualitatives ont été recueillies au moyen de questionnaires en ligne, analysées séparément, puis intégrées. Des professionnels de santé de 19 pays de l'Afrique francophones représentant tous les niveaux du système de santé ont participé à la phase 1. Plus des deux tiers des participants à la phase 1 ont déclaré avoir amélioré leurs connaissances techniques sur la BGF et formé 2 675 collègues. Environ 85 % des participants à la phase 1 ont trouvé le processus de revue par les pairs bénéfique, générant de nouvelles idées qui ont renforcé leur plan d'action. Même ceux qui ne l'ont pas trouvé utile avaient plus de chances de terminer la phase 1. Les relations sociales/externes ont conduit à un épanouissement personnel et à des impacts professionnels de haut niveau. Les réseaux formés ont créé des systèmes de soutien précieux pour les participants, et la certification de la formation a conduit à des opportunités d'élargissement des rôles, de promotion et d'augmentation des responsabilités. 255 participants ont terminé la phase 1 et 71 ont terminé la phase 2. Tous les participants ont déclaré avoir sensibilisé et formé 49 088 membres de la communauté sur la BGF. Les échanges entre pairs et le partage de connaissances locales ont permis de répondre aux besoins éducatifs immédiats à tous les niveaux du système de santé et ont favorisé l'action locale. Le modèle d'apprentissage virtuel entre pairs a permis d'atteindre avec succès des apprenants de divers horizons, en leur fournissant des connaissances qu'ils ont directement appliquées à des problèmes complexes dans leurs contextes, décolonisant ainsi efficacement l'approche de prise en charge de la BGF.

Texte S4
Manuscrit en français

Introduction

Décrite pour la première fois en 1899, la bilharziose génitale féminine (BGF) est une complication chronique résultant d'une infection par *Schistosoma haematobium*, un ver parasite [1]. La schistosomiase se transmet par contact avec des sources d'eau douce contaminées par des larves de schistosomes et se manifeste par une maladie intestinale ou urogénitale [3]. En l'absence de traitement, la schistosomiase peut entraîner une inflammation chronique et des pathologies progressives des voies urinaires et génitales chez les femmes et les filles, pouvant engendrer de graves complications de leur santé sexuelle et reproductive, notamment des grossesses extra-utérines, l'infertilité et des fausses couches [2]. Cette maladie est associée à un risque accru d'infection par le virus de l'immunodéficience humaine (VIH) et le Virus du Papillome Humain (VPH), contribuant ainsi à la stigmatisation et à l'isolement social [3]. Il est important de noter que la BGF est évitable et que des options de traitement précoce sont disponibles à faible coût [4,5].

Bien qu'elle touche des millions de femmes et de filles dans les régions endémiques, la BGF reste largement absente des cursus de formation médicale et infirmière. Lorsque la schistosomiase est enseignée, l'accent est généralement mis sur les formes urinaires et intestinales, les manifestations gynécologiques étant peu abordées [6]. Cette lacune curriculaire reflète une tendance plus générale à négliger les dimensions sexospécifiques des maladies tropicales négligées (MTN) [7]. La BGF n'est pas non plus intégrée aux services de santé sexuelle et reproductive ni aux services de lutte contre le VIH dans la plupart des pays endémiques. En l'absence de directives et de prise en charge de la BGF, les professionnels de santé (de première ligne) manquent d'outils, de ressources et de méthodes standardisées pour le diagnostic, le traitement et la référence [8,9]. Les formations continues et le développement professionnel continu (DPC) permettent aux professionnels de santé de combler leurs lacunes en matière de connaissances sur des affections telles que la BGF, mais dans de nombreux pays à revenu faible ou intermédiaire, ces formations ne sont pas obligatoires et la participation est limitée par des contraintes de financement, d'infrastructure et de ressources [10]. Par conséquent, dans les zones endémiques, le taux d'erreurs de diagnostic et de mauvais traitements est élevé chez les personnes touchées [11]. Les manifestations cliniques telles que les lésions génitales, les écoulements et l'infertilité sont souvent confondues avec des IST ou des affections liées au VIH, exposant ainsi les femmes à la stigmatisation et à des traitements inutiles [6].

Il a été avancé que l'une des raisons de ces problèmes réside dans le fait que la pratique et les textes médicaux sont souvent fondés sur la « norme mondiale » établie par les pays à revenu élevé [12]. Cette dépendance peut marginaliser les connaissances et les priorités des régions endémiques, où le fardeau de maladies telles que la BGF est le plus lourd. Les appels récents à la décolonisation de la santé mondiale ont incité à une réévaluation critique des dynamiques de pouvoir, des hypothèses et des pratiques qui façonnent les partenariats mondiaux en santé [12,13]. Par exemple, dans le contexte de la BGF, l'accès à l'eau, à l'assainissement et à l'hygiène (WASH) est un domaine crucial où les discussions sur la décolonisation ont émergé [14]. Historiquement, de nombreuses interventions WASH ont été guidées par des modèles occidentaux qui négligent les réalités locales, ce qui limite leur durabilité [14,15]. Une approche décolonisée préconise plutôt des solutions communautaires et adaptées au contexte qui s'appuient sur l'expertise locale et impliquent activement les communautés endémiques dans la prise de décision afin de garantir un impact à long terme [14,16].

Texte S4
Manuscrit en français

Formation et apprentissage par les pairs dans le cadre du programme BGF

Reconnaissant la BGF comme un enjeu majeur de justice sociale et de genre nécessitant une action urgente, deux organisations à but non lucratif, la Fondation Apprendre Genève (TGLF) et Bridges to Development, ont collaboré à la conception et à la mise en œuvre d'une formation virtuelle en deux phases, axée sur l'échange entre pairs et portant sur la BGF. En 2023, cette formation a été proposée aux professionnels de santé d'Afrique francophone (voir texte S1). La formation (phase 1) et l'Accélérateur d'Impact (phase 2) ont été élaborés selon le modèle d'apprentissage par les pairs de la TGLF [17-20]. Ce modèle, développé par le co-auteur RS, repose sur les principes fondamentaux de l'apprentissage informel et incidentel [21] et optimise le partage interdisciplinaire et interprofessionnel des connaissances grâce aux technologies numériques [22]. Le contenu de la formation s'appuie sur le Cadre de compétences BGF, élaboré par Bridges to Development en collaboration avec divers experts et l'Organisation mondiale de la Santé. Ce cadre décrit un ensemble complet de 27 compétences essentielles ou aptitudes requises pour la formation des professionnels de la santé à tous les niveaux du système de santé sur la BGF, couvrant le diagnostic, le traitement et la prévention dans les contextes cliniques et non cliniques [23].

La participation aux formations virtuelles basé sur l'apprentissage par les pairs sur la BGF en 2023 était volontaire et gratuite. Les participants ont été sélectionnés en fonction de leur capacité et de leur disponibilité à se connecter. Des efforts ont été déployés pour garantir une représentation diversifiée en termes de genre, de niveaux du système de santé et de rôles professionnels, en particulier dans les zones prioritaires des bailleurs de fonds, notamment en République démocratique du Congo. Le nombre de candidatures masculines étant supérieur à celui des candidatures féminines, une proportion plus élevée de femmes a été sélectionnée afin d'assurer une représentation plus équilibrée des genres. Les événements ont été organisés en français par TGLF avec le soutien du personnel de Bridges to Development et se sont déroulés sur une période de sept mois. Des experts du domaine ont présenté les concepts fondamentaux de la BGF et ont servi de guides. La figure 1 illustre le déroulement des événements (texte S1). Lors de la phase 1, les participants ont acquis les compétences de base sur la BGF et élaboré un plan d'action visant à améliorer la prise en charge de la BGF dans leurs communautés. Ces plans d'action ont été élaborés et revues par les participants et les experts à l'aide d'une grille d'évaluation fournie. Les conseils pour l'élaboration et la revue des plans d'action portaient sur la faisabilité, le respect des directives nationales, l'intégration avec d'autres programmes de santé et la capacité d'engagement communautaire. Lors de la phase 2, les participants ont bénéficié d'un soutien supplémentaire pour la mise en œuvre de leurs plans d'action. Ils ont reçu et partagé des recommandations et des ressources de leurs pairs et des experts. Afin d'approfondir notre compréhension de la capacité du modèle à atteindre divers apprenants et à combler les lacunes importantes des programmes de la BGF et de la formation des professionnels de santé, des recherches supplémentaires sont nécessaires.

Texte S4
Manuscrit en français

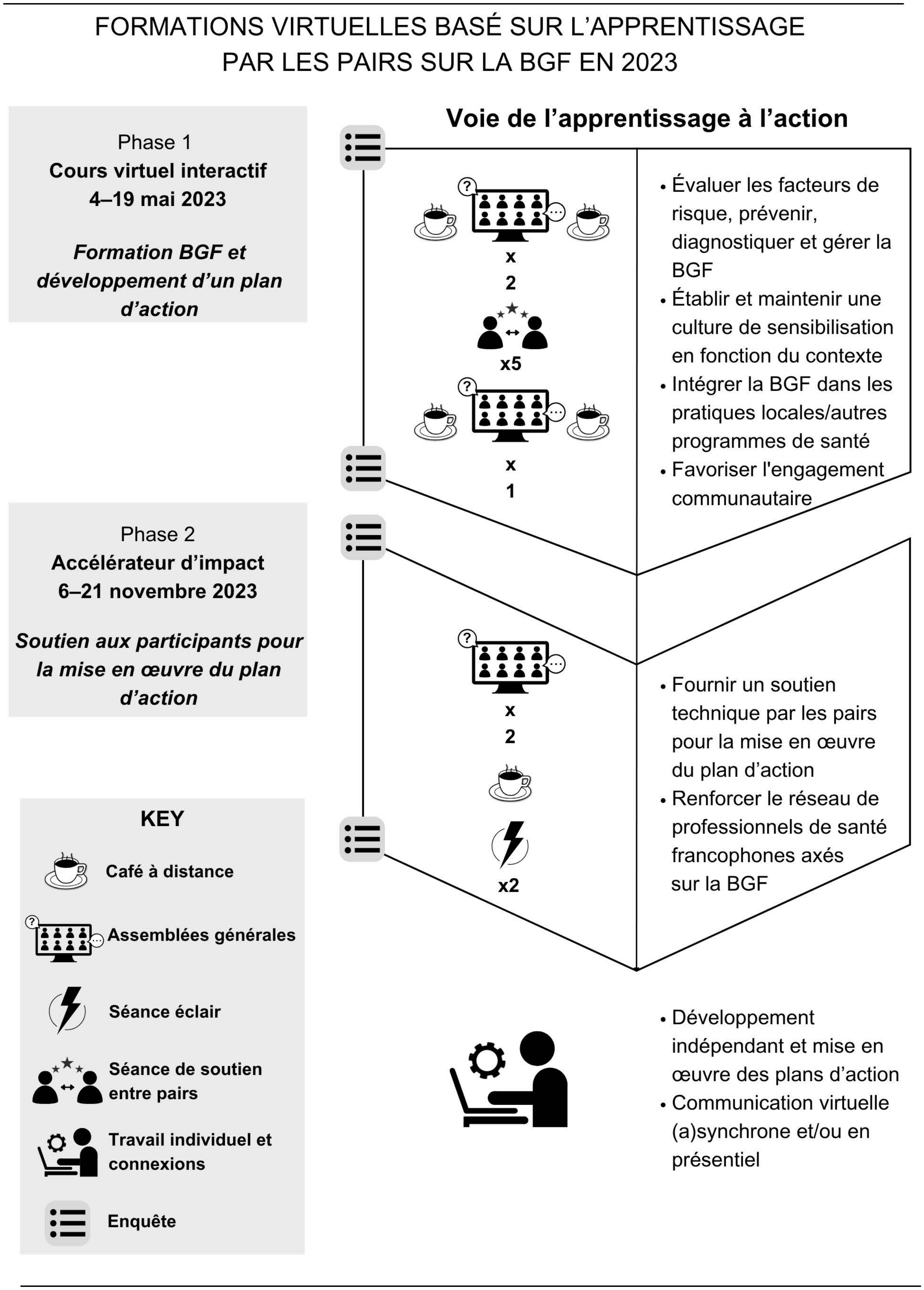

Fig 1 Calendrier et informations sur la formation 2023

Texte S4
Manuscrit en français

Cadre théorique

Le connectivisme a joué un rôle déterminant dans le développement de la formation en ligne [24] et offre ainsi une perspective précieuse pour évaluer des outils numériques tels que le modèle d'apprentissage par les pairs de la TGLF [17-20]. Comme illustré dans la figure 2, le connectivisme conçoit l'apprentissage comme un processus collaboratif reliant les individus et les sources d'information. L'accent mis par le connectivisme sur la création de connaissances aux niveaux individuel, conceptuel et social/externe [24,25] en fait un cadre pertinent pour examiner comment les participants au programme de formation virtuelle entre pairs sur la BGF de 2023 ont utilisé les technologies numériques pour accéder à des connaissances essentielles et collaborer afin de relever des défis sanitaires complexes liés à la BGF. Au niveau neuronal, les réseaux biologiques nous permettent de former des souvenirs ou des concepts et d'y attacher une signification au fur et à mesure que nous apprenons. Au niveau conceptuel, nous pouvons générer de nouvelles idées avec d'autres en partageant et en connectant des informations qui résonnent. Au niveau social/externe, les réseaux dont nous faisons partie influencent les ressources auxquelles nous avons accès et les connexions que nous pouvons établir [26,27].

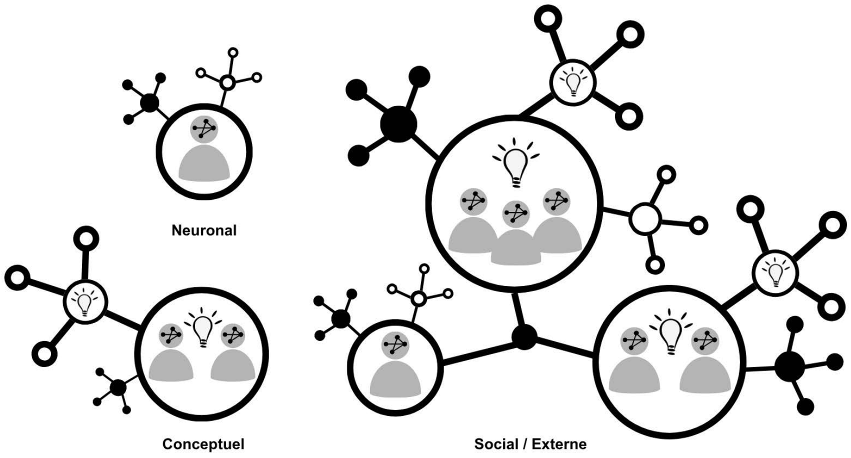

Fig 2 Les trois niveaux du connectivisme

Objectif et questions de la recherche

Cette recherche vise à explorer l'approche d'apprentissage, à documenter et à analyser la mise en œuvre et les résultats du programme 2023 de la formation virtuelle par l'apprentissage par les pairs sur la BGF pour l'Afrique francophone. Plus précisément, elle pose la question suivante : à travers la lentille du connectivisme, comment la formation virtuelle par les pairs sur la BGF en 2023 (phase 1) et l'Accélérateur d'impact (phase 2) ont-ils créé de la valeur pour des participants de divers horizons et ont-ils débouché sur des actions locales ? Les sous-questions suivantes ont été élaborées pour mesurer cette valeur et cet impact :

Sous-questions quantitatives :

1. Quels sont les facteurs qui ont influencé la probabilité que les participants terminent l'une ou l'autre phase ?
2. Dans quelle mesure les phases de formation ont-elles atteint les résultats escomptés, et comment le modèle d'apprentissage par les pairs de la TGLF a-t-il contribué à ces réussites ?

Sous-questions qualitatives :

3. Quels sont les apprentissages, les liens et les ressources qui ont créé de la valeur pour les participants ou les sous-groupes de participants ?
    a. Quelles connaissances et compétences techniques les participants ont-ils déclaré avoir acquises ?
    b. De quelle manière les participants ont-ils expliqué comment l'apprentissage par les pairs et la mise en réseau avec d'autres personnes ont renforcé leurs plans d'action ?
    c. Comment les récits des participants sur les diverses connexions et ressources ont-ils amélioré leur développement personnel et leur impact professionnel de haut niveau ?
    d. En quoi ceux qui ont mis en œuvre leur plan d'action et ceux qui ne l'ont pas fait ont-ils décrit leur expérience différemment ?

Méthodes

Déclaration d'éthique

Cette étude repose sur des données secondaires recueillies par la Fondation Apprendre Genève (TGLF), sous la supervision de sa Commission d'éthique de la recherche (CER). La CER de la TGLF respecte les principes de la Commission cantonale d'éthique de la recherche (CCER), de la Loi fédérale sur la recherche sur l'être humain (RS 810.30), de la Loi fédérale suisse sur la recherche sur l'être humain (LRH) et de l'Ordonnance sur les aspects organisationnels de la LRH (Ordonnance sur l'organisation de la LRH, Org LRH) pour la collecte, la gestion et la protection des données, et autorise les recherches proposées. Le 18 juin 2024, le co-auteur KE a reçu l'approbation de la CER de la TGLF pour mener un projet de recherche intitulé « Reconstituer les paysages mondiaux de l'apprentissage en santé » portant sur l'influence du modèle d'apprentissage par les pairs de la TGLF. Cette étude s'inscrit dans le cadre du projet de recherche approuvé par la CER de la TGLF. Le 12 juillet 2024, le comité d'éthique de l'Université de Géorgie a approuvé le projet et l'a classé comme recherche n'impliquant pas de sujets humains (PROJECT00009825).

Conception de la recherche

Cette recherche utilise une méthode mixte convergente et parallèle [19,20], accordant une importance égale aux deux volets de données (QUAN+QUAL). Les données secondaires quantitatives et qualitatives des deux phases du programme de formation virtuelle d'apprentissage par les pairs sur la BGF 2023 ont été collectées simultanément, analysées séparément, puis intégrées [30]. Pour atteindre l'objectif de la recherche, les méthodes de recherche quantitatives et qualitatives ont été triangulées afin de fournir une interprétation plus complète des données (Fig. 3).

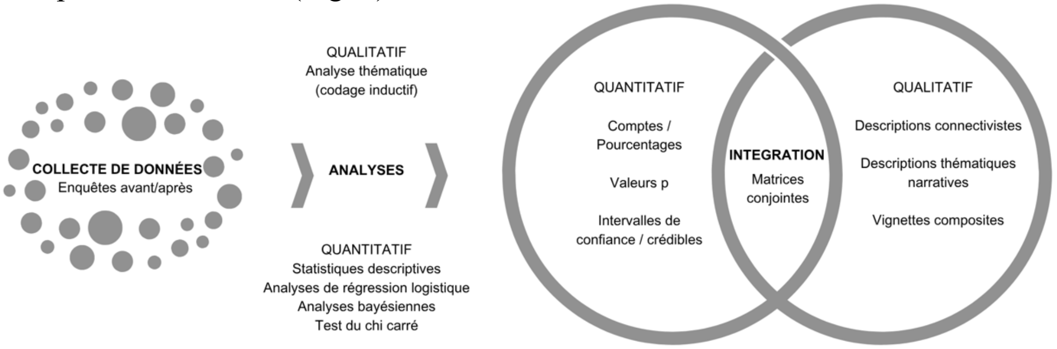

Fig 3 Diagramme de procédure de la recherche

Texte S4
Manuscrit en français

Collecte de données

La collecte de données comprenait des enquêtes en ligne avant et après la formation, élaborées par TGLF pour les deux phases du programme de formation virtuelle par des pairs sur la BGF 2023. Les liens vers les enquêtes, qui ont été créées à l'aide de TypeForm [31], ont été fournis à tous les participants par courriel. Les participants qui ont répondu aux enquêtes étaient des professionnels de santé francophones de tous les niveaux du système de santé qui ont participé à la formation de 2023. Avant d'interroger les participants, la TGLF les a informés par écrit de l'objectif de l'enquête et de l'utilisation qui pourrait être faite de leurs réponses (rapports, présentations, recherches, etc.). Dans le cadre de l'enquête, les participants ont consenti par écrit à ce que leurs données soient utilisées à des fins de recherche. Les participants ont répondu volontairement à l'enquête et n'ont pas été rémunérés pour cela. Ils pouvaient sauter toutes les questions auxquelles ils ne souhaitaient pas répondre. Les coauteurs JJ, NV, RS et CM ont eu accès aux informations d'identification des répondants pendant et après la collecte des données. Les vidéos et les textes liés aux participants, y compris ceux qui ont été partagés pendant les séances éclair, sont accessibles au public. Les données des enquêtes préalables à la formation ont été compilées le 27 juin 2023 et celles des enquêtes postérieures à la formation, le 12 décembre 2023.

Toutes les enquêtes comportaient des éléments quantitatifs et qualitatifs et permettaient de recueillir les changements déclarés par les participants concernant leurs connaissances sur les services de la BGF leur accès aux outils et ressources BGF, ainsi que leurs contacts au sein du réseau BGF. L'enquête préalable de la phase 1 recueillait également des informations sociodémographiques et des détails sur l'accès aux soins de santé des participants, ainsi que leurs attentes vis-à-vis du programme et leur capacité à y participer. L'enquête préalable de la phase 2 recueillait des informations actualisées sur ces points, recueillies lors de l'enquête préalable de la phase 1, ainsi que des données sur la collaboration des participants avec les autres membres du programme et sur l'évolution de leur plan d'action, notamment leurs besoins actuels et les mesures prises. Les enquêtes postérieures des phases 1 et 2 permettaient en outre de recueillir des informations sur l'évolution du plan d'action et de recueillir des informations auprès des participants sur leur expérience du programme, notamment sur les difficultés rencontrées et sur la manière dont ils se sont sentis soutenus dans leur développement personnel et professionnel.

Analyse des données

Des versions anonymisées des ensembles de données d'enquête ont été partagées avec tous les coauteurs le 12 janvier 2024, en vue d'un rapport au bailleur de fonds de la formation. L'analyse des données secondaires aux fins de la recherche a commencé le 12 juillet 2024. L'analyse quantitative et l'analyse qualitative ont été réalisées simultanément, puis séparément, suivies d'une analyse intégrative.

Analyse quantitative

L'analyse statistique quantitative effectuée comprenait des statistiques descriptives et des régressions logistiques et bayésiennes multiples. Les colonnes pertinentes ont été sélectionnées à partir de l'ensemble de données original afin d'inclure uniquement les variables pertinentes pour l'étude, et les modèles de régression ont intégré plusieurs covariables (sexe, profession, type d'organisation et expérience antérieure), ce qui a permis de traiter les variables confondantes. Certaines variables, telles que la profession et l'organisation, ont été transformées pour résoudre les problèmes de rareté des catégories et pour faciliter des comparaisons statistiques significatives. Les valeurs numériques de certaines variables, telles que l'impact de la

participation sur les relations sociales, ont été recodées en variables catégorielles afin d'en assurer l'interprétation et la cohérence.

Des statistiques descriptives ont été utilisées pour résumer les scores de connaissances avant et après la formation et les compétences autodéclarées, qui ont été enregistrées sous forme de réponses individuelles notées sur une échelle de 0 à 5. Les changements dans ces mesures ont donné un aperçu des gains d'apprentissage attribuables à la formation. La régression logistique multiple a permis d'identifier les prédicteurs significatifs de l'achèvement de la formation et de modéliser la probabilité d'une amélioration des connaissances en fonction des caractéristiques individuelles. Une approche progressive à rebours a été employée en utilisant le paquetage stats de R (version 4.4.2). Dans un premier temps, toutes les variables candidates ont été incluses dans le modèle, puis les variables ont été systématiquement supprimées une à une en fonction de leur valeur p et du critère d'information d'Akaike des modèles. Les variables dont les valeurs p n'étaient pas fiables ou indéfinies ont été exclues au cours de ce processus afin de garantir la stabilité et l'interprétabilité des modèles. Nous avons également évalué la multicolinéarité à l'aide du facteur d'inflation de la variance (VIF) et exclu les variables présentant des valeurs VIF élevées (> 5) afin d'éviter les problèmes d'instabilité des coefficients. Pour les modèles finaux, le seuil de signification était une valeur p inférieure à 0,005.

Étant donné la taille réduite de l'échantillon pour la phase 2, une régression logistique bayésienne a été appliquée pour estimer les effets des activités d'apprentissage par les pairs sur des résultats tels que l'acquisition de connaissances. Elle a été réalisée à l'aide du progiciel rstanarm, qui utilise la méthode de Monte Carlo hamiltonienne pour un échantillonnage a posteriori efficace. Les résultats ont été résumés à l'aide de la moyenne postérieure des coefficients et de leurs intervalles crédibles (IC) à 95 %. Les prédicteurs significatifs étaient ceux dont les IC n'incluaient pas zéro. Les facteurs de Bayes ont été calculés pour comparer les preuves des modèles et identifier les modèles les mieux adaptés. Les tracés ont montré un mélange régulier et des chaînes stables, confirmant la convergence et la fiabilité des estimations a posteriori.

Analyse qualitative

L'analyse qualitative a suivi le processus d'analyse thématique en six phases décrit par Braun et Clarke [32] et a été réalisée sous Excel. Les réponses ouvertes à l'enquête ont été traduites du français à l'anglais à l'aide d'un service de traduction automatique neuronale [33]. Les réponses sélectionnées ont été revues et corrigées en termes d'exactitude et d'intention par le personnel de la TGLF et de Bridges to Development, qui parlent couramment le français et l'anglais.

La première phase a consisté à lire les données plusieurs fois afin de clarifier et d'organiser les réponses. Au cours de la deuxième phase, les données ont été codées de manière inductive. Des codes préliminaires ont été attribués et discutés avec l'équipe. Au cours de la troisième phase, les codes ont été combinés, décrits et catégorisés à travers la lentille théorique du connectivisme. Dans la troisième phase, les codes ont été combinés et regroupés en trois catégories principales : défis, stratégies et résultats (tableau A de la figure S1 et du tableau). Par exemple, les codes « gérer les réticences » et « sécurité et stabilité du site » ont été classés dans la catégorie « défis de mise en œuvre, » les codes « plaidoyer auprès des principaux responsables de la santé » et « échelonnement du financement et des activités » dans la catégorie « stratégies de mise en œuvre, » et les codes « extrants » et « résultats » dans la catégorie « résultats de la mise en œuvre. » L'équipe a ensuite analysé ces catégories en fonction des participants ayant finalisé leur plan d'action et de ceux qui ne l'avaient pas fait, à travers le prisme théorique du

connectivisme. Dans la quatrième phase, l'équipe a examiné l'ensemble des données afin d'identifier les thèmes potentiels. Deux thèmes ont été créés au cours de la cinquième phase et les réponses liées à chaque thème ont été identifiées. Au cours de la sixième phase, des citations textuelles représentant chaque niveau de connectivisme ont été sélectionnées et des vignettes composites des expériences des travailleurs de la santé ont été rédigées pour décrire la complexité des thèmes d'une manière accessible à divers lecteurs [34,35]. Les premières ébauches des deux vignettes composites ont été rédigées avec l'aide de ChatGPT (25 février 2024). La décision de commencer par des ébauches initiales générées par l'IA a été prise pour aider à alimenter les citations au hasard des sélections qui, autrement, n'auraient peut-être pas été choisies en raison du nombre et de la longueur des réponses des participants. Les premières versions ont fait l'objet de nombreuses révisions afin de présenter les principaux éléments thématiques de manière ciblée et globale, tout en préservant la diversité sociodémographique des citations textuelles. Les vignettes finales proposent une synthèse narrative de chaque thème.

Analyse intégrative

L'analyse intégrative a débuté par la fusion de données quantitatives et qualitatives dans des matrices conjointes [30, 36] en lien avec l'objectif de l'étude. Les données fusionnées juxtaposaient des statistiques descriptives à des observations qualitatives et à des citations associées aux niveaux de connectivisme. Les données ont ensuite été analysées afin d'évaluer comment elles se confirmaient, s'enrichissaient, se complétaient ou divergeaient les unes des autres et de générer des méta-inférences. Un récit intégré a ensuite été rédigé pour synthétiser ces méta-inférences [30, 36-39].

Positionnement et réflexivité

En tant qu'équipe de recherche responsable de cette étude collaborative entre nos organisations, Bridges to Development et TGLF, nous sommes engagées dans la décolonisation de la santé mondiale. Nos organisations mettent l'accent sur les savoirs locaux, la diversité des représentations et l'autonomisation des professionnels de santé dans leurs contextes respectifs. Conformément à cet engagement, nous avons pris en compte et analysé les dimensions de nos identités pertinentes pour cette recherche dans une version de la matrice de transparence de Khan [40] que nous avons adaptée à notre travail (Texte S1).

Collectivement, nous formons un groupe diversifié, représentant différentes ethnies, professions et niveaux d'expertise en santé publique. Nos organisations sont impliquées depuis longtemps dans la problématique de la BGF et ont travaillé directement sur le terrain dans certains des pays touchés. Nous sommes unis par notre volonté de renforcer les capacités des professionnels de santé au sein des communautés qu'ils servent (Texte S2). Nos expériences de formation virtuelle utilisant la méthodologie d'apprentissage par les pairs de la TGLF ont été positives, et nous apprécions le type d'engagement et les résultats rapportés par les participants. Tout au long de nos échanges, nous avons appris les uns des autres et ensemble, et nous considérions avoir une bonne compréhension de la méthodologie ainsi que des défis et opportunités contextuels de la BGF ; une perspective dont nous étions conscients qu'elle pouvait biaiser notre analyse. Ainsi, à toutes les étapes de notre processus de recherche, nous nous sommes réunis régulièrement pour analyser les données, en remettant en question nos interprétations respectives avec respect et en veillant à ce que notre analyse reste ancrée dans les données. Afin d'accroître la fiabilité de nos résultats, nous avons triangulé les données en recoupant plusieurs sources et en partageant des citations textuelles. Nous avons également procédé à un audit des résultats, en nous assurant que des collègues des pays du Sud, membres à part entière de notre équipe et animateurs de programme, en aient examiné le contenu et la

présentation, et en réalisant une traduction non officielle du manuscrit accepté (Texte S3) et des informations complémentaires en français (Texte S4).

Résultats

Résultats quantitatifs

Participants à l'étude et leurs caractéristiques

Le tableau B (Fig. et du tableau S1) présente les caractéristiques des participants. Sur les 1686 professionnels de santé ayant postulé à la phase 1786 ont été retenus. Parmi ces derniers, 255 ont suivi la formation jusqu'au bout. On comptait 564 femmes et 1115 hommes parmi les candidats. Afin de promouvoir l'égalité des sexes, 386 femmes et 397 hommes ont été sélectionnés. La plupart des participants retenus étaient âgés de 35 ans et plus (63,6 %). La majorité résidait en Afrique centrale et du Moyen-Orient (48,7 %), notamment au Cameroun, en République centrafricaine, au Congo, en République démocratique du Congo, en Guinée équatoriale et au Gabon. Les participants représentaient diverses professions de santé, le groupe le plus important étant celui des médecins/gynécologues-obstétriciens (43,4 %), suivis des responsables de la santé publique (21,8 %) et des infirmiers/sage-femmes/infirmiers praticiens (21,2 %). Ils travaillaient également dans différents types d'établissements, principalement dans les hôpitaux et centres de santé publics (23,1 %), puis dans les services nationaux du ministère de la Santé (20,3 %) et les organisations privées (20,8 %). Pour la phase 2, 145 professionnels ont postulé et ont tous été acceptés. Parmi eux, 71 ont suivi la formation jusqu'au bout. Là encore, les hommes étaient plus nombreux à postuler (64,6 % des candidats) que les femmes (35,4 %).

Facteurs influençant la réussite de la formation

Le tableau C (figure et tableau S1) présente les facteurs ayant influencé la réussite des deux phases. Malgré une sélection plus importante de femmes pour la formation afin d'obtenir une cohorte plus équilibrée, les femmes avaient moins de la moitié des chances de réussir la phase 1 que les hommes (OR : 0,42 ; IC à 95 % : [0,22 ; 0,79] ; p = 0,008). Les participants n'ayant pas rencontré de difficultés avec les concepts ou la technologie de formation avaient plus de trois fois plus de chances de réussir la phase 1 que ceux ayant éprouvé des difficultés avec ces concepts ou le matériel pédagogique (OR : 3,45 ; IC à 95 % : [1,15 ; 10,34] ; p = 0,027). Les participants ayant financé eux-mêmes leurs dépenses (connexion internet, mise en œuvre du plan d'action, etc.) avaient plus de deux fois plus de chances de terminer la phase 1 que ceux pour lesquels ni eux ni leurs employeurs n'ont engagé de frais (OR = 2,45, IC : [1,26 ; 4,78], p = 0,008).

Pour la phase 2, les participants ayant déjà commencé la mise en œuvre de leur plan d'action présentaient une probabilité de réussite plus élevée que ceux n'ayant pas encore débuté (coef. : -1,19, IC à 95 % : 0,13-2,35). En revanche, les agents de santé communautaires présentaient une probabilité de réussite plus faible que les médecins (coef. : -1,75, IC à 95 % : -3,58-0,10).

Efficacité de la formation : Développement des connaissances et des capacités

Le tableau D (figure et tableau S1) présente les résultats des formations des phases 1 et 2. Parmi les participants retenus pour la phase 1, 14% n'avaient jamais entendu parler de la BGF et 62% n'avaient aucune expérience préalable avec cette maladie. La phase 1 visait à renforcer les connaissances et les compétences. 66% des participants ont rapporté une amélioration d'au moins deux niveaux de leurs connaissances et de leur confiance sur une échelle de 0 à 5. La proportion de participants ayant une connaissance approfondie de la BGF est passée de 18,0% à 47,2%. La proportion de participants se sentant capables de diagnostiquer la BGF est passée de 41% à 90%, celle des participants se sentant capables de le traiter de 37,1% à 86,4%, et celle des participants

se sentant capables de le prévenir de 57,5% à 96,6%. La proportion de participants se disant pleinement à l'aise pour aborder le sujet de la BGF avec leurs patients a également augmenté, passant de 19,8% à 55,9%. Le tableau F (figure et tableau S1) présente les facteurs influençant la probabilité d'acquérir plus d'un niveau de connaissance après la phase 1. Les agents de santé communautaires avaient 83 % moins de chances d'y parvenir que les médecins/gynécologues-obstétriciens (OR : 0,17 ; IC à 95 % : [0,04 ; 0,67] ; p = 0,012). De même, les participants ayant déjà une expérience de la prise en charge de la bilharziose génitale féminine avaient 83 % moins de chances de déclarer de tels progrès que ceux qui n'en avaient pas (OR : 0,17 ; IC à 95 % : [0,04 ; 0,71] ; p = 0,015).

Le tableau E (figure et tableau S1) présente l'impact plus large de la formation, au-delà des participants. Après la phase 2, 39% des participants ont diagnostiqué ou pris en charge des patientes atteintes de bilharziose génitale féminine, soit environ 638 cas. 91% des participants ont déclaré avoir formé un total de 2675 collègues. De plus, 8 % des participants ont mobilisé leurs communautés, informant plus de 49000 personnes sur la BGF.

Efficacité de la formation : Élaboration et mise en œuvre des plans d'action

Parmi les 232 plans d'action élaborés lors de la phase 1, 82% intégraient des stratégies d'intégration à d'autres programmes, tels que la lutte contre le VPH et le cancer du col de l'utérus, l'accès à l'eau, à l'assainissement et à l'hygiène (WASH), la prévention de l'infertilité, les infections sexuellement transmissibles (IST), le VIH/SIDA, les programmes scolaires, les soins périnatals, les programmes de lutte contre les maladies tropicales négligées (MTN), la vaccination et les questions de migration et d'immigration (figure A et tableau S1).

La phase 2 visait à apporter un soutien à la mise en œuvre de ces plans d'action. Comme le montre le tableau D (Fig. et du tableau S1), à la fin de la phase 1, 39 % des participants avaient commencé à mettre en œuvre leurs plans d'action, mais seulement 1% les avaient finalisés au moment de l'enquête post-formation. Avant la phase 2, on observait une légère augmentation du nombre de plans d'action finalisés parmi les participants à cette phase. Cependant, après la phase 2, 23 % des participants avaient finalisé leur plan d'action et 71 % avaient entamé sa mise en œuvre au moment de l'enquête post-formation.

La figure B de la Figure et tableau S1présente les trois principales catégories de plans d'action élaborés par les participants à la phase 2. Le groupe Sensibilisation et Prévention visait à sensibiliser la population sur la BGF et à promouvoir les mesures préventives. Le groupe Diagnostic et Prise en charge des cas s'est concentré sur l'amélioration des capacités de diagnostic, le traitement des cas de BGF, la gestion des orientations et l'enregistrement des cas pour le suivi. Le groupe Prise en charge globale combinait plusieurs objectifs, notamment la sensibilisation, la prévention, le diagnostic, le traitement, la référence et l'enregistrement. Afin d'identifier les personnes ayant élaboré chaque type de plan d'action, nous avons examiné les professions des participants et constaté des corrélations claires (figure C de la figure et du tableau S1). Les médecins, représentant près de 25 % des participants, étaient à l'origine de la plupart des plans de prise en charge globale. Les agents de santé communautaires se sont concentrés sur la sensibilisation et la prévention, tandis que les techniciens de laboratoire ont exclusivement élaboré des plans axés sur le diagnostic et la prise en charge des cas. Cette convergence était également manifeste lorsqu'on examinait si les rôles des participants incluaient la réalisation d'examens pelviens. Parmi ceux dont les objectifs étaient liés au diagnostic et à la prise en charge des cas, 60% ont déclaré effectuer des examens pelviens dans le cadre de leurs fonctions. La proportion a atteint 68,4% chez les participants ayant des objectifs de prise en charge globale. En revanche, seulement 35,9% des participants ayant des objectifs de

sensibilisation et de prévention ont déclaré pratiquer des examens pelviens dans le cadre de leurs activités professionnelles, ce qui reflète la moindre importance accordée à la pratique clinique dans ce groupe.

Impact du modèle d'apprentissage par les pairs de TGLF

Le tableau G de la Figure et tableau S1présente les résultats des modèles de régression bayésienne examinant l'influence du modèle d'apprentissage par les pairs de la TGLF [17-20]. L'acquisition des connaissances a été positivement influencée par le soutien des pairs. Les participants ayant jugé ce soutien utile présentaient une probabilité d'acquisition des connaissances plus élevée que ceux ne l'ayant pas jugé utile (coefficient : 1,97 ; IC à 95% : [0,80 ; 3,30]).

Parmi les participants de la phase 1, 61% ont indiqué dans le questionnaire post-formation avoir appris davantage que prévu grâce à la revue par les pairs. De plus, 86% des participants ont déclaré que la revue par les pairs avaient considérablement amélioré leurs propres plans d'action, tandis que 85% ont jugé bénéfique la revue des plans d'action de leurs collègues. 85% ont indiqué avoir constaté des changements significatifs dans leur pratique professionnelle grâce à leur participation.

Une proportion importante de participants (77,2%) a contacté les pairs après la phase 1 à des fins de collaboration. Parmi eux, 61% ont également attribué le maintien du contact avec leurs pairs après le programme au modèle de soutien par les pairs. Ces collaborations ont notamment consisté à élaborer des projets communs, à fournir une assistance à la mise en œuvre, à soutenir les initiatives en cours et à travailler ensemble sur de nouveaux projets (Fig. D dans la Figure et du tableau S1).

Résultats qualitatifs

Connexions neuronales : Connaissances techniques et compétences acquises en matière d'BGF

Les participants ont entamé la phase 1 avec des niveaux de connaissance variables concernant le diagnostic, le traitement et la prévention de la BGF. Certains participants, bien qu'ils soient en première ligne ou impliqués dans l'élaboration de politiques, n'avaient aucune connaissance préalable de la BGF. Les informations reçues ont renforcé leurs connaissances générales sur la maladie et leurs capacités de diagnostic, les préparant ainsi à mieux discuter et traiter les cas au-delà de la simple distribution de médicaments. Les participants ont également expliqué que la formation avait permis de corriger des informations erronées et des erreurs de diagnostic. Ils ont constaté l'impact positif de ces nouvelles connaissances sur leurs pratiques cliniques et sur la vie des patients qui ont bénéficié de leur expertise accrue. Par conséquent, ils ont étendu la portée de la formation et permis à un plus grand nombre de personnes d'acquérir des connaissances et des compétences relatives à la BGF.

> *« Plus de 500 personnes ont été sensibilisées à la maladie, 103 prestataires de soins de santé ont été formés à la définition et à la détection rapide des cas dans cinq établissements de santé, dont deux privés.* » - Médecin travaillant avec une association à but non lucratif

Connexions conceptuelles : Apprentissage par les pairs et mise en réseau pour renforcer le plan d'action

La grande majorité des participants ont estimé que le soutien des pairs était précieux tout au long de la formation. Certains ont estimé que l'interaction avec les pairs était plus bénéfique que les documents fournis. Les participants ont également signalé que l'élaboration de leurs plans d'action avait été améliorée à la fois par le processus formel d'évaluation par les pairs et par la

mise en réseau informelle avec des pairs en dehors des canaux officiels. Cette interaction entre pairs a aidé les participants à clarifier les attentes et leur a permis d'apprendre des succès et des défis de chacun. Les participants ont également reconnu que le processus d'évaluation leur avait apporté le soutien et le retour d'information nécessaires pour améliorer la présentation de leurs idées.

> *« La revue par les pairs m'a permis d'améliorer mon plan d'action. Par exemple, un pair m'a rappelé que le titre de mon plan devait commencer par un verbe d'action. Un autre m'a suggéré de trouver une meilleure carte des districts sanitaires... car celle que j'avais incluse dans le plan d'action initial n'était pas très expressive. Ces deux exemples, pour n'en citer que quelques-uns, montrent à quel point la revue par les pairs reste essentielle.* » - Responsable de la santé publique d'une organisation à but non lucratif

De nombreux participants ont indiqué que le maintien des échanges avec leurs pairs après la fin de la phase 1 avait joué un rôle crucial dans la mise en œuvre de leurs plans d'action. Ils ont expliqué avoir tiré des enseignements des témoignages d'autres participants sur différentes plateformes, ce qui leur a permis de mieux comprendre comment mettre en œuvre efficacement leurs plans. Ceux qui ont mené à bien leurs plans d'action ont souligné que l'intégration aux programmes existants était essentielle à leur réussite, leur fournissant les ressources et les données nécessaires pour coordonner les interventions des différentes entités (Fig. A de la Fig. et du tableau S1). Les participants ayant rencontré des difficultés ont sollicité l'avis de leurs pairs.

> *« Elle m'a aidée à ne pas me décourager, mais plutôt à persévérer dans mon projet malgré les complications, en l'adaptant à la situation. »* – Médecin dans un hôpital/établissement de santé privé

Les obstacles communs comprenaient l'indisponibilité des médicaments, le manque de financement et la nécessité d'obtenir l'adhésion des autorités sanitaires. Ils ont surmonté ces obstacles en plaidant auprès des responsables de la santé et des leaders communautaires, en échangeant des conseils et des solutions avec leurs pairs et en consultant des experts et des mentors. Les réseaux de soutien entre pairs développés dans chaque pays ont joué un rôle déterminant pour surmonter les difficultés de mise en œuvre et atteindre leurs objectifs.

> *« Lorsque je n'ai pas pu déployer mon plan d'action faute de moyens financiers, j'étais complètement désorientée. C'est alors qu'en suivant des témoignages en direct et en lisant des articles sur Telegram, j'ai découvert les méthodes utilisées par d'autres Scholars qui, comme moi, n'avaient pas les mêmes ressources financières, pour atteindre les populations à risque. Ces connaissances m'ont permis de revoir mon approche. Il est clair que lorsque nous participons à un tel tremplin (l'Accélérateur d'Impact), nous sommes influencés par de nouvelles idées et par d'autres Scholars issus de divers horizons. »* – Agent de santé communautaire travaillant au niveau du district

Connexions sociales/externes : Diverses connexions et ressources pour la croissance et l'impact

Les interactions entre pairs ont élargi les points de vue des participants de plusieurs façons. Elles ont favorisé la prise de conscience du lien entre les réalités locales et les tendances régionales plus générales, notamment les défis structurels communs tels que l'accès limité à l'eau et ses conséquences sur la santé. Elles ont également encouragé une nouvelle prise de conscience de la nécessité d'aborder les maladies telles que la BGF par une approche collaborative et écosystémique. Les participants ont reconnu que les interactions entre pairs avaient renforcé leur confiance et leur avaient permis de croire en leur capacité à apporter des changements au sein de leurs communautés et de leurs sphères d'influence. Ces interactions ont non seulement renforcé

leur désir d'approfondir leurs connaissances sur la BGF, mais les ont également motivés à partager ces connaissances avec leurs communautés. En outre, les participants ont fait état d'un renforcement de leurs capacités de communication, notamment en matière d'écoute active, de tolérance et de synthèse concise des idées dans des délais impartis. Ils ont décrit leur évolution personnelle en matière de pensée critique, notamment dans la reconnaissance de la valeur de la collaboration transfrontalière pour relever les défis sanitaires à grande échelle.

« Cette formation m'a permis de comprendre que je suis désormais citoyen du monde et que mon simple conseil à un collègue peut lui être utile pour résoudre un problème de santé publique ou sauver une vie à l'autre bout du monde. » – Agent de santé publique travaillant dans une organisation à but non lucratif

Les participantes ont expliqué comment la certification leur avait ouvert des perspectives d'évolution et de promotion. Certaines se sont vu confier des responsabilités supplémentaires, comme le suivi des cas de BGF au niveau du district, tandis que d'autres ont constaté une augmentation de la demande des patientes et une confiance accrue de la communauté. Elles ont également décrit une plus grande proactivité dans leurs fonctions professionnelles, ce qui a amélioré leur performance au travail. La formation a élargi leurs réseaux professionnels, tant au niveau national qu'international, créant des systèmes de soutien facilitant la consultation sur les cas de BGF et un accès plus rapide à l'information. Ces réseaux ont facilité la collaboration sur des initiatives, renforçant leur capacité à plaider auprès des autorités sanitaires et à influencer les politiques et les pratiques en promouvant l'accès au dépistage, au traitement et aux services de soutien pour les femmes touchées.

« Les gens m'écoutent quand je parle de la BGF, et les femmes veulent se faire dépister pour la BGF. » - Médecin travaillant dans un hôpital public/établissement de santé

Résultats thématiques

L'analyse des catégories de codes entre les participants ayant mené à bien leur plan d'action et ceux ne l'ayant pas fait a révélé deux thèmes. Le premier, « Accélérer les liens et l'intégration, » est apparu comme un point commun chez les personnes ayant mené à bien leur plan d'action. À l'inverse, le second, « connexions connectivistes malgré les complexités, » a été l'expérience prédominante chez ceux qui n'ont pas mené à bien leur plan.

Thème 1 : Accélérer les connexions et l'intégration. Parmi les participants ayant mené à bien leur plan d'action, la stratégie la plus courante a été l'intégration des activités. L'intégration a fourni les ressources et les données nécessaires pour coordonner une réponse accélérée entre les différentes entités. Pour parvenir à cette intégration, les participants ont mis en avant les connexions nouvelles et améliorées avec les informations, les pairs et les experts en la matière grâce aux événements de la formation virtuelle BGF. Ces connexions ont renforcé leur capacité de plaidoyer auprès des décideurs du secteur de la santé et leur ont permis de mener à bien les activités de leur plan d'action, qu'un budget soit disponible ou non. L'intégration BGF a couvert la sensibilisation, le diagnostic et le traitement, du niveau local au niveau national, et a été facilitée par la collaboration entre les agents communautaires. L'intégration s'est déroulée dans le cadre des services de santé privés et publics de routine, y compris les services de santé maternelle dans des domaines spécifiques, et a également été intégrée dans les programmes de lutte contre les maladies tropicales négligées. Grâce à l'intégration des activités, les participants ont indiqué que la mise en œuvre de leurs plans d'action avait conduit à des améliorations dans la couverture sanitaire et les actions locales.

Texte S4
Manuscrit en français

Vignette composite (Thème 1). En participant aux événements de formation, j'ai appris que le BGF est *« une maladie négligée. »* Les conversations que j'ai eues avec d'autres chercheurs dans des *« zones endémiques et non endémiques de la BGF »* m'ont permis de *« jeter un regard global sur l'épidémiologie de la BGF dans le monde »* et de découvrir comment elle a été *« précédemment ignorée et pourtant commune dans notre communauté. »* Je me suis sentie obligée de partager ces informations avec mes collègues et j'ai élaboré des *« présentations PowerPoint sur le BGF. »* Après avoir écouté ce que j'avais appris, ils étaient *« convaincus, comme moi, que certaines choses peuvent être faites pour mieux protéger les femmes et les filles contre certaines maladies. »* Nous nous sommes mis au travail pour « sensibiliser le médecin de zone, le directeur de l'hôpital et le gestionnaire de données de la zone de santé... Malgré le défi que représente le manque de ressources, *»* ils ont suggéré de mener *« un plaidoyer avec le partenaire qui soutient la lutte contre les MTN et certainement de capitaliser sur les ressources allouées à d'autres MTN... »* En travaillant ensemble, nous avons commencé à nous former lors de réunions cliniques régulières en utilisant *« l'Atlas de l'Organisation mondiale de la santé »* comme guide. Nous avons également créé un *« groupe d'alerte WhatsApp »* pour signaler en temps réel les cas suspects de BGF. Nous avons organisé des *« communications par radio »* et collaboré avec les relais communautaires, les organisations non gouvernementales, les agents des centres de jeunes et l'école de médecine. Avec autant d'activités en cours, le soutien et les conseils des experts pendant le cycle d'apprentissage ont été d'une valeur inestimable pour *« choisir les actions »* et les conseils des autres apprenants ont *« renforcé notre capacité... à travailler de manière efficace et efficiente. »* Grâce à nos efforts, nous avons constaté un *« changement de comportement de la population »* et nous avons maintenant élargi nos *« hypothèses de diagnostic différentiel. » « La bilharziose (BGF) est une importante infection parasitaire endémique dans le monde »* et *« l'accélérateur était comme un chien de garde ou un coq qui venait nous encourager à courir à l'avance, »* ce qui nous a aidés à réaliser nos projets.

Thème 2 : Connexions connectivistes malgré les complexités. Les participants n’ayant pas finalisé leur plan d’action ont indiqué progresser et avoir besoin de plus de temps pour intégrer leurs activités aux plans locaux et nationaux. Les raisons de ce délai supplémentaire étaient diverses : volonté de fonder les projets sur des données non encore collectées ou partagées, attente de décisions de financement, obtention des autorisations des autorités sanitaires, et nécessité d’ajuster le calendrier de mise en œuvre pour des raisons personnelles ou d’autres activités communautaires. Dans plusieurs cas, les plans d’action ont été élaborés pour couvrir plusieurs régions, ce qui a permis d’étendre leur impact au-delà du délai imparti. Par ailleurs, dans certaines zones, l’indisponibilité de médicaments pour traiter la BGF a entravé la finalisation des plans d’action. Dans ces circonstances, les participants ont jugé utile de solliciter l’aide des autorités sanitaires et des responsables communautaires, d’échanger des conseils et des idées avec leurs pairs et de consulter des experts ou des mentors. En adoptant une approche progressive de la planification des actions, les participants ont persévéré malgré l’attente et ont trouvé des solutions alternatives pour améliorer l’accès aux soins et l’action locale.

Vignette composite (Thème 2). J'ai commencé *« aussi vite que possible à mettre en œuvre »* mon plan pendant l'Accélérateur d'impact. Cependant, j'ai eu l'impression de me heurter à un mur à chaque fois. J'ai trouvé quelques *« données ou documents utilisables »* relatifs à la BGF dans notre communauté, mais ils n'étaient pas suffisants. Mes réunions avec les responsables de la santé et de la communauté ont suscité de l'intérêt, mais la proposition a été *« bloquée au niveau des autorisations. » « Cet accélérateur m'a beaucoup aidé, en particulier dans la planification des activités. »* En entrant en contact avec des pairs, j'ai eu *« beaucoup*

d'autres idées » et stratégies. Par exemple, *« j'ai mené des enquêtes pour connaître la proportion de femmes touchées. »* Ensuite, j'ai fait une *« demande écrite à la direction pour obtenir l'autorisation de mettre en œuvre notre plan. »* La réponse m'a demandé de *« former le personnel à l'utilisation de ce médicament et à la BGF. »* J'ai joint *« mes forces à celles d'autres professionnels de la santé »* et nous avons fait des présentations *« sur la BGF lors des réunions de travail du matin dans les structures de santé. »* Nous avons profité de ces occasions pour aider à clarifier *« la confusion entre la BGF et les infections sexuellement transmissibles, »* et nous avons discuté *« largement de la BGF sous forme d'un débat, où chacun donne son point de vue basé sur l'expérience et les données scientifiques. » « Afin d'apprendre davantage de leurs expériences professionnelles, »* nous avons planifié de futures formations en ligne pour poursuivre les conversations. Les responsables de la santé commencent maintenant *« à encourager sa vulgarisation et... à se l'approprier. »* Nous *« avons des demandes qui sont en cours de traitement, »* mais pour l'instant, nous faisons *« avec le peu que nous avons pu collecter. »* Jusqu'à présent, *« nous avons traité 10 cas de BGF, formé 21 agents de santé et orienté 24 agents communautaires. » « Grâce à cette participation, je ne savais rien sur le BGF, mais aujourd'hui, je suis devenue une grande enseignante. Grâce à cette participation, je suis connecté à un monde d'experts qui peuvent m'aider à résoudre un problème sur la BGF. » « Nous ne terminerons pas dans les délais comme prévu, mais « le plan est en cours. »*

Résultats intégrés

Comme décrit dans les sections précédentes, les phases 1 et 2 de la formation virtuelle entre pairs sur la BGF en 2023 ont été bénéfiques aux participants, indépendamment de leur sexe, de leur profession, de leur rôle au sein du système de santé et de leur origine démographique. Les participants ont acquis des connaissances et une meilleure sensibilisation, et la plupart ont entrepris des actions et progressé, même lorsque leurs plans d'action n'étaient pas entièrement finalisés. La synthèse de ces méta-inférences offre une compréhension plus approfondie et plus complète de l'impact connectiviste du programme. Le tableau H (Fig. et du tableau S1) illustre comment la formation a accru les connaissances et la confiance des participants, tout en étendant son impact grâce à leurs actions en matière de soins aux patients, de formation entre pairs et d'engagement communautaire. Le tableau I (Figure et tableau S1) montre comment les plans d'action ont été élaborés, intégrés et mis en œuvre, soulignant leur adéquation aux rôles professionnels, le rôle central de l'intégration et les processus pilotés par les pairs qui ont permis la mise en œuvre malgré les obstacles. Le tableau J (figure et tableau S1) montre comment le soutien et les interactions entre pairs ont renforcé les connaissances, la pratique professionnelle et l'élaboration des plans d'action, tout en favorisant un engagement continu grâce à des réseaux qui ont soutenu la collaboration, l'apprentissage et le plaidoyer au-delà de la formation.

Discussion

Cette étude à méthodologie mixte présente l'approche et les méthodes de la formation sur la BGF utilisant le modèle d'apprentissage par les pairs de la TGLF [17-20]. Les résultats montrent comment cette approche de formation a permis d'atteindre un groupe diversifié de professionnels de santé qui ont perçu la valeur de la formation et ont entrepris des actions ayant un impact plus large grâce aux connaissances acquises.

L'apprentissage par les pairs est largement utilisé dans la formation des professionnels de santé et présente de nombreux avantages, notamment des améliorations sociales, comportementales et cognitives [41, 42]. Le modèle de la TGLF s'appuie sur l'apprentissage par les pairs et intègre également des éléments d'apprentissage par l'action. McGill et Brockbank

[43] décrivent l'apprentissage par l'action comme un processus continu d'apprentissage et de réflexion visant à atteindre des résultats concrets. Le modèle de la TGLF intègre les dimensions réflexives et pratiques de l'apprentissage par l'action, tout en privilégiant l'apprentissage entre pairs plutôt que de s'appuyer uniquement sur un facilitateur ou un expert pour la formation [17, 18, 44, 45]. Les résultats de cette étude montrent que la formation, utilisant le modèle et le contenu adapté de la BGF développé par Bridges, a permis aux participants de gagner en confiance pour discuter, diagnostiquer et prendre en charge les cas de BGF. Conformément aux résultats de Stone et al. [46], ils ont également constaté une amélioration de leurs capacités d'écoute, de leur réflexion critique, de leur communication, de leur proactivité, de leur performance dans leurs fonctions et de leur capacité à défendre efficacement leurs intérêts.

Cette approche d'apprentissage par les pairs, axée sur la mise en œuvre, s'inscrit dans la théorie du connectivisme, qui souligne l'importance des liens diversifiés pour l'apprentissage continu et la génération de nouvelles idées [24-26]. Dans cette étude, des liens authentiques ont été tissés durant la formation, créant un environnement inclusif et interactif favorisant l'apprentissage continu, la réflexion, le retour d'information et la collaboration [47-49]. Les collaborations nationales et transfrontalières ont permis aux participants de bénéficier d'un soutien pour la mise en œuvre de leurs plans d'action, d'un accès rapide à l'information et de plateformes collectives de plaidoyer, tant pendant qu'après la formation. Ceci reflète la vision connectiviste selon laquelle le savoir ne réside pas dans un seul individu, mais émerge de la force des réseaux et des pratiques partagées.

McGill et Brockbank [43] notent que l'apprentissage par l'action est bénéfique tant pour les individus que pour les organisations. Notre étude montre comment la formation dispensée selon le modèle TGLF a mené à des actions locales ayant influencé la santé communautaire : des patients ont été atteints, des collègues formés et des communautés mobilisées. Les participants ont également renforcé leur crédibilité et la confiance professionnelle qui leur est accordée. Ainsi, le programme a atteint son objectif de promouvoir un accès équitable à l'information et aux ressources, tout en permettant l'élaboration de plans d'action qui ont positionné les participants en mesure de mener des initiatives de transformation du système de santé pertinentes pour leur contexte.

Makau-Barasa et al. [50] affirment que les interventions contre les maladies tropicales négligées (MTN) sont affaiblies par une compréhension contextuelle limitée et une faible implication communautaire. Cette formation a permis de redonner du pouvoir aux professionnels locaux, en valorisant leurs connaissances et en soutenant les actions émanant des communautés touchées. Kwete et al. [51] soulignent l'idée reçue selon laquelle les agents de santé des pays à revenu faible et intermédiaire (PRFI) ne seraient pas capables de résoudre leurs propres problèmes de santé. Nos résultats contredisent cette idée et rejoignent d'autres travaux de recherche [52-55] montrant que les professionnels des PRFI peuvent élaborer des solutions durables et adaptées au contexte culturel. Pourtant, la plupart des campagnes mondiales de santé, notamment celles contre le paludisme, le VIH/sida et la tuberculose, restent menées par des institutions des pays du Nord. Certains chercheurs s'opposent à ce que les organisations de santé privilégient les solutions technologiques aux problèmes de santé des pays du Sud, car celles-ci n'apportent souvent que des améliorations à court terme plutôt que durables [56]. L'accent est plutôt mis sur le renforcement des systèmes, le développement des capacités locales et les alternatives durables, ce que cette formation soutient. Bien que nous ayons facilité la mise en place de cette formation, le processus n'a pas été marqué par une dynamique de « sauveur blanc.

Texte S4
Manuscrit en français

» Les participants se sont plutôt mutuellement formés et soutenus pour concevoir et mettre en œuvre des actions adaptées aux contextes locaux.

Des études antérieures ont souligné l'importance d'intégrer des interventions spécifiques à une maladie ou à un secteur dans des services de santé plus vastes afin d'améliorer la couverture et l'efficacité des soins, comme l'intégration des initiatives de services de prise en charge de la BGF aux services de lutte contre le VIH, l'eau, le WASH et le cancer du col de l'utérus [4, 23, 57, 58]. Par conséquent, l'intégration aux services existants a été une priorité pour de nombreux participants lors de la phase 1, car elle constituait une méthode culturellement appropriée pour atteindre leurs objectifs. Les participants ont identifié plus de 10 opportunités d'intégration de programmes, selon leurs diverses perspectives, notamment dans les domaines du VPH/cancer du col de l'utérus, du WASH, de l'infertilité, des IST et du VIH/sida. Fait intéressant, l'intégration du programme de lutte contre les maladies tropicales négligées (MTN) figurait au huitième rang, ce qui souligne l'importance de proposer des formations à différents corps de métiers, institutions, niveaux du système de santé et organisations afin d'identifier de nouvelles opportunités d'intégration.

Nos résultats viennent étayer les données probantes sur les difficultés rencontrées dans la mise en œuvre des interventions contre les MTN dans les PRFI. Les participants ont rencontré des obstacles tels que l’insuffisance de ressources, des retards d’autorisation et des pénuries de médicaments essentiels. L’accès limité aux médicaments et aux soins de santé demeure un obstacle majeur à l’élimination des MTN [59]. La Feuille de route 2021-2030 de l’OMS pour les MTN [60] préconise une approche multisectorielle et systémique pour surmonter ces obstacles. Cette étude contribue à cet objectif en abordant un élément crucial : le renforcement des connaissances et des capacités des professionnels de santé. En réfléchissant collectivement à des solutions pour surmonter ces difficultés avec leurs pairs et en s’appuyant sur leurs réseaux, les participants ont développé des capacités individuelles et collectives d’intervention et, dans certains cas, ont amélioré les actions locales au-delà des plans initiaux.

Cette formation a touché avec succès un public diversifié dans plusieurs pays. Cependant, malgré nos efforts pour promouvoir la diversité, les femmes et les agents de santé communautaires étaient moins susceptibles de s’inscrire au programme et de le terminer. Une explication possible pourrait être le manque de temps dû à leurs responsabilités accrues [53, 61-63]. Les disparités historiques observées dans les taux d'achèvement des formations expliquent pourquoi les femmes sont moins susceptibles de terminer les programmes en raison de leurs responsabilités accrues [63]. Les agents de santé communautaires étaient également systématiquement moins susceptibles de terminer les deux formations que les médecins et présentaient une probabilité plus faible d'acquérir de nouvelles compétences après la phase 1. Ces agents de santé communautaires sont des intervenants de première ligne essentiels, en contact fréquent avec les communautés locales [52]. Leur sous-représentation limite la diversité des connaissances et des expériences partagées concernant la sensibilisation, le diagnostic, l'orientation et le traitement de la BGF, ainsi que la surveillance épidémiologique de routine au sein des communautés. Approfondir notre compréhension de leurs expériences pourrait améliorer le modèle de formation et l'adapter afin de soutenir et de renforcer leur participation.

Limites

Nous reconnaissons plusieurs limites à notre étude. Notre recherche a utilisé des mesures autodéclarées, ce qui pourrait introduire un biais de réponse. Bien qu'un taux de réponse de 31 % été suffisant pour ces analyses, et que nous ayons obtenu des taux de réponse de 42 % pour la phase 1 et de 49 % pour la phase 2, des taux de réponse plus élevés auraient pu fournir une

image plus complète. Notre méthodologie ne nous a pas permis d'approfondir les raisons pour lesquelles les femmes et les agents de santé communautaires étaient moins susceptibles de terminer la formation. Nous n'avons pas non plus pu évaluer l'impact des plans d'action sur les communautés, le système de santé ou l'état de santé des patients. En outre, bien que le contexte de cette étude soit représentatif des divers paysages de la santé mondiale en Afrique francophone, les résultats peuvent ne pas être généralisables à d'autres régions. Cependant, nous nous attendons à ce que les enseignements tirés de cette recherche aient une valeur transférable dans d'autres contextes et dans d'autres programmes d'apprentissage par les pairs.

Conclusion

Les programmes d'apprentissage par les pairs, comme la formation virtuelle sur la BGF de 2023, renforcent les partenariats en matière d'éducation médicale en reliant les professionnels de la santé au-delà des frontières géographiques et professionnelles. En décentrant les connaissances des experts, en favorisant les connexions entre pairs et en donnant la priorité au partage des connaissances locales, ce programme de formation a répondu à des besoins éducatifs immédiats tout en contribuant à un cadre plus large et plus équitable pour l'éducation à la santé mondiale. Pour Bridges to Development et TGLF, il s'agit d'une étape importante vers la décolonisation de la santé mondiale. L'objectif est d'autonomiser les praticiens locaux, de valoriser leurs contributions et de promouvoir des solutions durables et adaptées au contexte, susceptibles d'améliorer les résultats en matière de santé et de soins de santé. Cette étude démontre que les programmes d'apprentissage par les pairs utilisant les technologies numériques favorisent la collaboration au sein de la communauté et établissent des réseaux de soutien et des partenariats mondiaux où les professionnels peuvent échanger des conseils, partager leurs expériences et relever collectivement des défis communs. Ces connexions favorisent l'innovation et le renforcement des capacités grâce à des échanges interdisciplinaires et interprofessionnels dans le domaine des soins de santé. Comme l'a très bien dit un participant à cette étude, « *(lorsque nous) réunissons plus de vingt nationalités de manière interactive sur une plateforme virtuelle... nous pouvons influencer le monde.* »

Recommandations

Sur la base des enseignements tirés de cette recherche, les recommandations spécifiques pour les programmes de formation en maladies infectieuses sont les suivantes : (1) exploiter le potentiel des méthodologies participatives et des technologies numériques pour démanteler les déséquilibres historiques de pouvoir dans la santé mondiale et favoriser des changements véritablement durables au sein des communautés locales, (2) évaluer comment les plans d'action profitent aux communautés, au système de santé et aux patients, (3) soutenir les participants dans la création de stratégies et de communications efficaces pour plaider en faveur de l'intégration de leurs plans d'action dans les services et programmes existants, et (4) accroître la recherche collaborative avec les participants de manière à ce qu'ils soient intéressés et capables d'identifier les obstacles à l'apprentissage et à la pleine participation.

Les facteurs contextuels et l'évolution rapide des menaces qui pèsent sur la santé mondiale, comme le changement climatique, remettent en question la capacité des praticiens et des systèmes de santé mondiaux à prévenir, traiter et atténuer les maladies infectieuses [61]. Par conséquent, nous recommandons en outre d'adopter l'approche d'apprentissage par les pairs de la TGLF utilisant les technologies numériques [17-20] pour relever d'autres défis en matière de santé. Cette approche apportera de nouvelles perspectives, connaissances et expériences vécues dans le dialogue mondial et accélérera les processus de résolution des problèmes [44,64]. Ces processus participatifs encouragent également la mutualité et la responsabilité nécessaires pour

rétablir les relations et améliorer la portée et les résultats [65-67]. Pour les maladies négligées comme la BGF, nos recherches indiquent que cette approche favorise des partenariats plus inclusifs et équitables, amplifie l'action au niveau communautaire et renforce la capacité de nos systèmes mondiaux à réagir.

Informations complémentaires

Texte S1
Données S1
Texte S2
Figures et tableaux S1
Texte S3
Texte S4

Remerciements

Nous tenons à remercier les participants au programme de formation virtuelle sur la BGF de 2023 pour leur dévouement au partage des connaissances au sein de leurs communautés, entre eux et avec la communauté mondiale dans son ensemble. Nous tenons également à remercier les experts en la matière pour leurs contributions tout au long des événements et leur désir d'apprendre des participants et avec eux.

Références

1. Madden FC, Eng CS. A case of bilharzia of the vagina. Lancet. 1899;3596: 1716.
2. World Health Organization [WHO], Joint United Nations Programme on HIV/AIDS [UNAIDS]. No more neglect–female genital schistosomiasis and HIV: integrating sexual and reproductive health interventions to improve women's lives. [Internet]. Geneva: UNAIDS; 2019. Available from: https://www.who.int/publications/i/item/UNAIDS-JC2979
3. Shukla JD, Kleppa E, Holmen S, Ndhlovu PD, Mtshali A, Sebitloane M, et al. The association between female genital schistosomiasis and other infections of the lower genital tract in adolescent girls and young women: a cross-sectional study in South Africa. J Low Genit Tract Dis. 2023;27: 291–296. doi:10.1097/LGT.0000000000000756
4. Umbelino-Walker I, Wong F, Cassolato M, Pantelias A, Jacobson J, Kalume C. Integration of female genital schistosomiasis into HIV/sexual and reproductive health and rights and neglected tropical diseases programmes and services: a scoping review. Sex Reprod Health Matters. 2023;31: 2262882. doi:10.1080/26410397.2023.2262882
5. Mazigo HD, Samson A, Lambert VJ, Kosia AL, Ngoma DD, Murphy R, et al. "We know about schistosomiasis but we know nothing about FGS": a qualitative assessment of knowledge gaps about female genital schistosomiasis among communities living in schistosoma haematobium endemic districts of Zanzibar and Northwestern Tanzania. PLoS Negl Trop Dis. 2021;15: e0009789. doi:10.1371/journal.pntd.0009789
6. Christinet V, Lazdins-Helds JK, Stothard JR, Reinhard-Rupp J. Female genital schistosomiasis (FGS): from case reports to a call for concerted action against this neglected gynaecological disease. Int J Parasitol. 2016;46: 395–404. doi:10.1016/j.ijpara.2016.02.006
7. Theobald S, MacPherson EE, Dean L, Jacobson J, Ducker C, Gyapong M, et al. 20 years of gender mainstreaming in health: lessons and reflections for the neglected tropical diseases community. BMJ Glob Health. 2017;2: e000512. doi:10.1136/bmjgh-2017-000512
8. Iqbal U, Rabrenovic M, Li YC. Health care quality challenges in low- and middle-income countries. Int J Qual Health Care. 2019;31: 165–165. doi:10.1093/intqhc/mzz031

9. Martinez SG, Mbabazi PS, Sebitloane MH, Vwalika B, Mocumbi S, Galaphaththi-Arachchige HN, et al. The WHO atlas for female-genital schistosomiasis: co-design of a practicable diagnostic guide, digital support and training. PLOS Glob Public Health. 2024;4: e0002249. doi:10.1371/journal.pgph.0002249
10. Merry L, Castiglione SA, Rouleau G, Létourneau D, Larue C, Deschênes MF, et al. Continuing professional development (CPD) system development, implementation, evaluation and sustainability for healthcare professionals in low- and lower-middle-income countries: a rapid scoping review. BMC Med Educ. 2023;23:498. doi:10.1186/s12909-023-04427-6
11. Mazigo HD, Samson A, Lambert VJ, Kosia AL, Ngoma DD, Murphy R, et al. Healthcare workers' low knowledge of female genital schistosomiasis and proposed interventions to prevent, control, and manage the disease in Zanzibar. Int J Public Health. 2022;67: 1604767. doi:10.3389/ijph.2022.1604767
12. Hussain M, Sadigh M, Sadigh M, Rastegar A, Sewankambo N. Colonization and decolonization of global health: which way forward? Glob Health Action. 2023;16: 2186575. doi:10.1080/16549716.2023.2186575
13. Eichbaum QG, Adams L V., Evert J, Ho MJ, Semali IA, Van Schalkwyk SC. Decolonizing global health education: rethinking institutional partnerships and approaches. Academic Medicine. 2021;96: 329–335. doi:10.1097/ACM.0000000000003473
14. Mehjabeen D, Patel K, Jindal RM. Decolonizing global health: a scoping review. BMC Health Serv Res. 2025;25: 828. doi:10.1186/s12913-025-12890-8
15. De Wit S, Luseka E, Bradley D, Brown J, Bhagwan J, Evans B, et al. Water, sanitation and hygiene (WASH): the evolution of a global health and development sector. BMJ Glob Health. 2024;9. doi:10.1136/bmjgh-2024-015367
16. Lue JM, Bah S, Grant K, Lee J, Nzekele L, Tidwell JB. Principles for increasing equity in WASH research: understanding barriers faced by LMIC WASH researchers. BMJ Glob Health. 2023;8. doi:10.1136/bmjgh-2022-010990
17. Umbelino-Walker I, Szylovec AP, Dakam BA, Monglo A, Jones I, Mbuh C, et al. Towards a sustainable model for a digital learning network in support of the Immunization Agenda 2030 –a mixed methods study with a transdisciplinary component. PLOS Global Public Health. 2024;4. doi:10.1371/journal.pgph.0003855
18. Watkins KE, Sandmann LR, Dailey CA, Li B, Yang SE, Galen RS, et al. Accelerating problem-solving capacities of sub-national public health professionals: an evaluation of a digital immunization training intervention. BMC Health Serv Res. 2022;22. doi:10.1186/s12913-022-08138-4
19. Sadki R. What learning science underpins peer learning for Global Health? [Internet]. RedaSadki.Me; 2023. doi:10.59350/wmnja-epd48
20. Sadki R. How can we reliably spread evidence-based practices at the speed and scale modern health challenges demand? [Internet]. RedaSadki.Me; 2024. doi:10.59350/cqxmj-3bd96
21. Watkins KE, Marsick VJ, Wofford MG, Ellinger AD. The evolving Marsick and Watkins (1990) theory of informal and incidental learning. New Dir Adult Contin Educ. 2018;2018: 21–36. doi:10.1002/ace.20285
22. The Geneva Learning Foundation. The Geneva Learning Foundation [TGLF]. [Internet]. 2024. Available from: https://www.learning.foundation/
23. Jacobson J, Pantelias A, Williamson M, Kjetland EF, Krentel A, Gyapong M, et al. Addressing a silent and neglected scourge in sexual and reproductive health in Sub-Saharan Africa by development of training competencies to improve prevention, diagnosis, and treatment of female

genital schistosomiasis (FGS) for health workers. Reprod Health. 2022;19. doi:10.1186/s12978-021-01252-2
24. Corbett F, Spinello E. Connectivism and leadership: harnessing a learning theory for the digital age to redefine leadership in the twenty-first century. Heliyon. 2020;6: e03250. doi:10.1016/j.heliyon.2020.e03250
25. Dunaway MK. Connectivism: learning theory and pedagogical practice for networked information landscapes. Reference Serv Rev. 2011;39: 675–685. doi:10.1108/00907321111186686
26. Siemens G. Connectivism: a learning theory for the digital age. [Internet]. Elearnspace; 2004. Available from: https://elearnspace.org/Articles/connectivism.htm
27. UGA Mary Frances Early College of Education. Learning through complexity: George Siemens. [Internet]. YouTube; 2021. Available from: https://youtu.be/KZjqtU_Fgc8?feature=shared
28. Creswell JW, Plano Clark VL. Designing and conducting mixed methods research. 3rd ed. Thousand Oaks: Sage; 2018.
29. Creswell JW, Klassen AC, Clark VLP, Smith KC. Best practices for mixed methods research in the health sciences. Office of Behavioral and Social Sciences Research. Bethesda: National Institutes of Health; 2011. Available from: https://obssr.od.nih.gov/research-resources/mixed-methods-research
30. Guetterman TC, Fetters MD, Creswell JW. Integrating quantitative and qualitative results in health science mixed methods research through joint displays. Ann Fam Med. 2015;13: 554–561. doi:10.1370/afm.1865
31. Typeform. [Internet]. 2023. Available from: https://www.typeform.com
32. Braun V, Clarke V. Using thematic analysis in psychology. Qual Res Psychol. 2006;3: 77–101. doi:10.1191/1478088706qp063oa
33. DeepL. [Internet]. 2023. Available from: https://www.deepl.com/en/translator
34. Fader N, Legg E, Ross A. Finding a sense of community in youth soccer: a composite vignette of the refugee experience. J Park Recreat Admi. 2020;38: 2–20. doi:10.18666/JPRA-2020-9981
35. Jasinski L, Nokkala T, Juusola H. Reflecting on the value of vignettes in higher education research: toward a preliminary typology to guide future usage. Eur J High Educ. 2021;11: 522–536. doi:10.1080/21568235.2021.1999841
36. Guetterman TC, Fàbregues S, Sakakibara R. Visuals in joint displays to represent integration in mixed methods research: a methodological review. Methods Psychol. 2021;5: 100080. doi:10.1016/j.metip.2021.100080
37. Schoonenboom J. Developing the meta-inference in mixed methods research through successive integration of claims. In: Hitchcock JH, Onwuegbuzie AJ, editors. The Routledge Handbook for Advancing Integration in Mixed Methods Research. London: Routledge; 2022. pp. 55–70. doi:10.4324/9780429432828-6
38. Skamagki G, King A, Carpenter C, Wåhlin C. The concept of integration in mixed methods research: a step-by-step guide using an example study in physiotherapy. Physiother Theory Pract. 2024;40: 197–204. doi:10.1080/09593985.2022.2120375
39. Teddlie C, Tashakkori A, Johnson RB. Foundations of mixed methods research: integrating quantitative and qualitative approaches in the social and behavioral sciences. Thousand Oaks: Sage; 2009.
40. Khan SA. Decolonising global health by decolonising academic publishing. BMJ Glob Health. 2022;7. doi:10.1136/bmjgh-2021-007811

41. Guraya SY, Abdalla ME. Determining the effectiveness of peer-assisted learning in medical education: a systematic review and meta-analysis. J Taibah Univ Med Sci. 2020;15: 177–184. doi:10.1016/j.jtumed.2020.05.002
42. Ross MT, Cameron HS. Peer assisted learning: a planning and implementation framework: AMEE Guide no. 30. Med Teach. 2007;29: 527–545. doi:10.1080/01421590701665886
43. McGill I, Brockbank A. The Action Learning Handbook: Powerful Techniques for Education, Professional Development and Training. 1st ed. London: Routledge; 2003. doi:10.4324/9780203416334
44. Eller K. Brackish connections: (digital) learning networks, (virtual) communities of practice, and the rich learning-to-action pathways of their combined and intersecting existence. Adv Dev Hum Resour. 2024;27: 27–36. doi:10.1177/15234223241299704
45. Sadki R. Rethinking the “webinar”: sage on screen, guide on side, or both. [Internet]. RedaSadki.Me; 2019. doi:10.59350/j0qmx-stg25
46. Stone R, Cooper S, Cant R. The value of peer learning in undergraduate nursing education: a systematic review. ISRN Nurs. 2013;2013: 1–10. doi:10.1155/2013/930901
47. Freire P. Pedagogy of the oppressed. New York: Continuum; 2005.
48. Mezirow J. Learning as transformation: critical perspectives on a theory in progress. San Francisco: Jossey-Bass; 2000.
49. Cope B, Kalantzis M. Towards a new learning: the scholar social knowledge workspace, in theory and practice. E-Learn Digit Media. 2013;10: 332–356. doi:10.2304/elea.2013.10.4.332
50. Makau-Barasa LK, Kamara K, Karutu C, Aderogba M, Leaning E, Bockarie M. Systems thinking in the prevention, control, and elimination of neglected tropical diseases (NTDs). Int J Infect Dis. 2025;152: 107810. doi:10.1016/j.ijid.2025.107810
51. Kwete X, Tang K, Chen L, Ren R, Chen Q, Wu Z, et al. Decolonizing global health: what should be the target of this movement and where does it lead us? Glob Health Res Policy. 2022;7. doi:10.1186/s41256-022-00237-3
52. Afzal MM, Pariyo GW, Lassi ZS, Perry HB. Community health workers at the dawn of a new era: 2. planning, coordination, and partnerships. Health Res Policy Syst. 2021;19: 103. doi:10.1186/s12961-021-00753-7
53. Ahmed S, Chase LE, Wagnild J, Akhter N, Sturridge S, Clarke A, et al. Community health workers and health equity in low- and middle-income countries: systematic review and recommendations for policy and practice. Int J Equity Health. 2022;21: 49. doi:10.1186/s12939-021-01615-y
54. Bain LE, Adeagbo OA, Avoka CK, Amu H, Memiah P, Ebuenyi ID. Decolonising global (public) health: from western universalism to global pluriversalities. BMJ Glob Health. 2020;5: e002947. doi:10.1136/bmjgh-2020-002947
55. Bua E, Sahi SL. Decolonizing the decolonization movement in global health: a perspective from global surgery. Front Educ (Lausanne). 2022;7: 1033797. doi:10.3389/feduc.2022.1033797
56. Kumar R, Khosla R, McCoy D. Decolonising global health research: Shifting power for transformative change. PLOS Global Public Health. 2024;4. doi:10.1371/journal.pgph.0003141
57. Lamberti O, Bozzani F, Kiyoshi K, Bustinduy AL. Time to bring female genital schistosomiasis out of neglect. Br Med Bull. 2024;149: 45–59. doi:10.1093/bmb/ldad034
58. Preston A, Vitolas CT, Kouamin AC, Nadri J, Lavry SL, Dhanani N, et al. Improved prevention of female genital schistosomiasis: piloting integration of services into the national health system in Côte d’Ivoire. Front Trop Dis. 2023;4: 1308660. doi:10.3389/fitd.2023.1308660

59. Hudu SA, Jimoh AO, Adeshina KA, Otalike EG, Tahir A, Hegazy AA. An insight into the success, challenges, and future perspectives of eliminating neglected tropical disease. Sci Afr. 2024;24. doi:10.1016/j.sciaf.2024.e02165
60. World Health Organization [WHO]. Ending the neglect to attain the sustainable development goals: a road map for neglected tropical diseases 2021-2030. [Internet]. Geneva: World Health Organization; 2020. Available from: https://www.who.int/publications/i/item/9789240010352
61. El-Sayed A, Kamel M. Climatic changes and their role in emergence and re-emergence of diseases. Environ Sci Pollut Res. 2020;27: 22336–22352. doi:10.1007/s11356-020-08896-w
62. O'Donovan J, O'Donovan C, Kuhn I, Sachs SE, Winters N. Ongoing training of community health workers in low-income and middle-income countries: a systematic scoping review of the literature. BMJ Open. 2018;8. doi:10.1136/bmjopen-2017-021467
63. Raukar NP, Mishkin HM. An exploration of the influence of gender and domestic responsibilities on the trajectory of one's career. In: Stonnington CM, Files JA, editors. Burnout in Women Physicians: Prevention, Treatment, and Management. Cham: Springer; 2020. pp. 69–76. doi:10.1007/978-3-030-44459-4_4
64. Eller K. Learning to (co)evolve: a conceptual review and typology of network design in global health virtual communities of practice. Interdiscip J Inf Knowl Manag. 2024;19. doi:10.28945/5353
65. Robertson P, Jorgensen M, Garrow C. Indigenizing evaluation research: How Lakota methodologies are helping "raise the tipi" in the Oglala Sioux Nation. Am Indian Q. 2004;28: 499–526. Available from: https://link.gale.com/apps/doc/A132163756/AONE?u=uga&sid=bookmark-AONE&xid=993a65e6
66. Call-Cummings M, Dazzo GP, Hauber-Özer M. Critical participatory inquiry: an interdisciplinary guide. Thousand Oaks: SAGE Publications; 2023. Available from: https://collegepublishing.sagepub.com/products/critical-participatory-inquiry-1-275385
67. Dazzo GP. On restorative validity: reorienting inquiry toward peace, justice, and healing. The Qualitative Report. 2024;29: 403–421. doi:10.46743/2160-3715/2024.5690
