# Supplementary material for: Decolonizing infectious disease programs: A mixed methods analysis of a novel multi-country virtual training for Female Genital Schistosomiasis
Source: PLOS Glob Public Health. 2025 Dec 8;5(12):e0004235. doi: 10.1371/journal.pgph.0004235 (PMC12685162; doi:10.1371/journal.pgph.0004235)
Supplement: S5 Text — (PDF) [file pgph.0004235.s006.pdf]

Texte S1

Informations complémentaires sur la formation
2023 Événements de formation virtuelle d'apprentissage par les pairs sur la BGF pour l'Afrique francophone.

En 2021, la première phase d'une formation virtuelle destinée aux professionnels de santé et portant sur l'intégration de la BGF dans leur pratique a été testée en Afrique anglophone et francophone[1]. Sur 1527 candidatures, 484 professionnels de santé ont été sélectionnés et plus de 300 participants ont suivi la formation jusqu'au bout. Le processus de sélection visait à garantir une répartition égale des genres et à assurer un équilibre entre les participants issus des établissements de santé (par exemple, les cliniciens) et ceux issus d'autres secteurs (par exemple, les agents de santé communautaires). Les participants répondant à ces critères ont été sélectionnés selon le principe du premier arrivé, premier servi. Les personnes indisponibles aux dates et heures prévues ou dans l'incapacité de se connecter n'ont pas été retenues. Celles qui n'ont pas été sélectionnées ont été encouragées à poursuivre leur apprentissage en visionnant les séances enregistrées ou en participant aux sessions ultérieures. La deuxième phase, appelée « Accélérateur d'Impact », a été mise en place pour répondre au besoin de retours d'information et de soutien en temps réel, permettant ainsi à l'équipe de mieux comprendre les difficultés rencontrées par les participants et de leur apporter une aide opportune. Les participants à la formation de 2021 ont déclaré avoir formé 2052 professionnels de santé supplémentaires, pris en charge 3892 filles et femmes et diffusé des informations essentielles sur la bilharziose génitale féminine (BGF) (prévention, symptômes, traitements) auprès de 120666 membres et responsables communautaires peu ou pas informés de la maladie. Ces succès ont démontré le potentiel de cette approche de formation pour lutter contre cette maladie négligée. En 2023, l'END Fund a financé une nouvelle session de formation (phases 1 et 2) pour les participants d'Afrique francophone.

Toutes les sessions de formation virtuelle entre pairs sur la BGF (phases 1 et 2) de 2023 ont été animées par la TGLF avec le soutien de l'équipe de Bridges to Development. Des experts ont présenté les concepts clés de la BGF et ont servi de guides. Ces sessions ont permis aux participants d'apprendre les uns des autres. Le travail hors ligne des participants sur leurs plans d'action et les échanges directs entre pairs ont complété les formations. Les phases 1 et 2 sont décrites ci-dessous, suivies de liens vers des informations complémentaires sur la formation et les ressources pour les apprenants.

Phase 1 : Cours de formation virtuel

Au cours de la phase 1, les participants ont acquis les compétences fondamentales sur la BGF et élaboré un plan d'action visant à améliorer les résultats de la BGF dans leurs communautés. Cette phase a débuté par deux assemblées générales, au cours desquelles les participants ont pu : (1) découvrir la BGF, (2) discuter de scénarios et de cas concrets liés à la BGF, et (3) obtenir des informations et des ressources pour l'élaboration de leurs plans d'action. Un café à distance (virtuel) facultatif de 30 minutes a été proposé à tous les participants via un lien unique afin de leur permettre d'échanger, de poser des questions et de développer leur réseau avant et après les

[1] Les pays francophones comprenaient : le Bénin, le Burkina Faso, le Cameroun, le Congo, la Côte d'Ivoire, le Gabon, la Guinée, Madagascar, le Mali, le Maroc, le Niger, la République démocratique du Congo, le Sénégal, le Tchad et le Togo. Les pays anglophones comprenaient : le Cameroun, le Ghana, le Kenya, le Libéria, Madagascar, le Malawi, le Nigéria, les Émirats arabes unis et la Zambie.

assemblées. Par la suite, cinq séances de soutien par les pairs ont été organisées pour consolider l'élaboration des plans d'action et permettre leur revues par les pairs. Les plans d'action ont été élaborés et revues par les participants et des experts sur une plateforme en ligne, à l'aide d'une grille d'évaluation fournie. Les conseils pour l'élaboration et la revue des plans d'action portaient sur la faisabilité, le respect des directives nationales, l'intégration aux autres programmes de santé et la capacité d'engagement communautaire. La formation s'est conclue par une troisième assemblée générale permettant : (1) de faire le point sur les progrès du groupe et l'élaboration des plans d'action, (2) de discuter des enseignements tirés du processus de revue par les pairs, (3) de recueillir les commentaires des experts, et (4) de découvrir des moyens de rester en contact. Le détail de chaque événement est présenté ci-après.

Assemblée générale 1 (obligatoire) | 4 mai 2023 (~3 heures)
(*Café à distance*)→Accueil et introduction→Café à distance [1:1]→Présentation de la BGF et questions-réponses→Exercice d'apprentissage en équipe : Discussions de groupe basées sur des scénarios→Réflexions et annonces→(*Café à distance)*

La première assemblée générale a commencé par un accueil d'environ 10 minutes et une introduction par les facilitateurs de l'événement, au cours de laquelle les experts ont raconté comment elles ont découvert la BGF et pourquoi elles s'en préoccupent. L'assemblée s'est ensuite poursuivie par un "café à distance" de ~5 minutes permettant aux participants de rencontrer un autre participant dans des salles de réunion, après quoi les experts ont présenté la BGF et ont répondu aux questions des participants posées à voix haute ou dans le dialogue en directe pendant ~30 minutes. Un bref aperçu des discussions de groupe basées sur des scénarios a ensuite été fourni à tous les participants par les facilitateurs de la séance, et au cours des deux heures suivantes, des discussions en petits groupes ont été organisées pour examiner deux scénarios. Pour chaque discussion en petit groupe, les participants ont commencé par examiner ensemble les détails du scénario et la meilleure façon de les aborder en petits groupes pendant environ 30 minutes, avant de revenir discuter de leurs réflexions par l'ensemble du groupe pendant environ 30 minutes supplémentaires. Pendant les discussions dans l'ensemble du groupe, les experts ont contribué en établissant des liens entre les compétences de base sur la BGF et les rapports de travail des petits groupes et en posant des questions ou des idées supplémentaires à prendre en considération. La première assemblée s'est terminée par ~5 minutes de réflexion et d'annonces par les facilitateurs de la séance et les experts.

Assemblée générale 2 (obligatoire) | 9 mai 2023 (~3 heures)
(*Café à distance*)→Bienvenue et révision→Information sur le plan d'action BGF→Exercice d'apprentissage en équipe : Discussions de groupe sur les défis réels des BGF→Réflexions et annonces→(*Café à distance)*

La deuxième assemblée générale a commencé par un accueil d'environ 15 minutes et un examen du programme par les facilitateurs de séance et les experts, après quoi ils ont consacré environ 20 minutes à l'échange sur le processus et les critères d'élaboration du plan d'action BGF. Après le premier format d'assemblée générale pour le brainstorming collaboratif, les participants ont ensuite tenu des discussions en petits groupes et en assemblée pendant deux heures sur les défis BGF de la vie réelle soumis par les participants qui ont accepté que leurs cas soient examinés

pour un soutien supplémentaire. L'assemblée s'est terminée par environ 15 minutes de réflexion et d'annonces par les facilitateurs de séance et les experts.

Séances de soutien par les pairs <u>(facultatif)</u> | 5 séances, 10-16 mai 2023 (~1 heure)
Bienvenue→Revue d'information→Discussion et Q&R

Les séances de soutien par les pairs ont commencé par un bref accueil et un examen des informations sur le sujet choisi par les facilitateurs de la formation. Les thèmes choisis pour les trois premières séances portaient sur le développement des plans d'action (comment élaborer, compléter, soumettre et démarrer), et les deux dernières sessions portaient sur le processus de revue par les pairs (comment réaliser et soumettre des revues). Après avoir examiné les informations présentées sur le sujet choisi, une discussion de groupe ouverte est organisée, au cours de laquelle les participants ont pu discuter de leurs liens avec le sujet et poser des questions. Les sessions se sont ensuite concentrées sur l'écoute d'un participant partageant son défi BGF et les actions qu'il allait entreprendre pour le résoudre, avec le feedback et les commentaires des pairs et des experts. Au cours de certaines séances, les participants ont également discuté de ce qu'ils apprenaient du processus de revue par les pairs et de la manière dont ils allaient utiliser ces connaissances pour améliorer leurs plans d'action. Au cours de toutes les séances, une assistance technique a été fournie aux participants qui avaient des difficultés à utiliser la plateforme de soumission des plan d'action et de revue par les pairs.

Troisième assemblée générale <u>(obligatoire)</u> | 17 mai 2023 (~2 heures)
(*Café à distance*)→Bienvenue, examen des progrès du groupe et récapitulation →Présentations des participants et retour d'information des PME→Cérémonie de clôture et annonces→(*Café à distance)*

La troisième assemblée générale a commencé par un mot de bienvenue d'environ 20 minutes, un examen de l'élaboration du plan d'action de l'ensemble du groupe et une récapitulation des points importants de la deuxième assemblée générale par les experts et les facilitateurs. Ensuite, au cours des 80 minutes suivantes, les participants ont présenté leurs plans d'action et ont discuté avec l'ensemble du groupe des enseignements tirés du processus de revue par les pairs. Les experts ont fourni un retour d'information aux participants afin de renforcer les plans d'action. L'assemblée s'est terminée par une cérémonie de clôture d'environ 20 minutes et des annonces finales. Au cours de la cérémonie de clôture, les participants, les experts et les facilitateurs de l'événement ont célébré les progrès réalisés par le groupe au cours de la phase 1. Les annonces finales comprenaient une invitation à rejoindre la phase 2, l'accélérateur d'impact, et d'autres groupes et programmes de BGF, Bridges to Development et TGLF tels que la communauté de pratique sur le schisto génital, 24 communautés des Scholars TGLF basées dans les pays, et les événements Teach to Reach: Connect.

**Une plateforme en ligne, Peergrade, a été utilisée pour soumettre et réviser les plans d'action. Le 11 mai 2023, les participants ont soumis leur projet de plan d'action pour la revue par leurs pairs. Le 16 mai 2023, les participants ont soumis leurs évaluations des plans d'action de trois pairs. Le 19 mai 2023, les participants ont soumis leurs propres plans d'action révisés et améliorés.*

Phase 2 : Accélérateur d'impact

Durant la phase 2, les participants ont bénéficié d'un soutien accru pour la mise en œuvre de leurs plans d'action, notamment par le biais d'assemblées générales, d'un café à distance et de séances éclair. Ils ont également travaillé individuellement à la mise en œuvre de leurs plans et ont communiqué avec leurs pairs en dehors des événements. Les deux premières semaines de la phase 2 ont constitué la rampe de lancement de l'Accélérateur d'Impact. Lors des deux assemblées générales de cette rampe, les participants ont (1) présenté l'avancement de la mise en œuvre de leur plan d'action, (2) fixé un objectif précis à atteindre à la fin des deux semaines, (3) partagé des expériences réussies et des difficultés rencontrées, et (4) tiré des enseignements de leurs apprentissages. Ils ont reçu et partagé des recommandations et des ressources de leurs pairs et d'experts. Après cette rampe, les participants ont été invités à participer à un café à distance à leur convenance. Deux séances éclair ont ensuite permis à certains participants de partager l'expérience de mise en œuvre de leur plan d'action. Leurs réussites et leurs difficultés ont favorisé les échanges et la planification des événements futurs pour tous les participants. Vous trouverez ci-dessous le détail de chaque événement.

Rampe de lancement : Assemblée générale 1 (obligatoire) | 6 novembre 2023 (~1 heure)
Bienvenue et vue d'ensemble→Partage entre pairs et fixation d'objectifs→Retour des experts→Annonces et clôture

La première assemblée générale a commencé par un accueil d'environ 15 minutes des participants et une présentation de la phase 2 par les facilitateurs de la séance. L'aperçu comprenait des informations sur le calendrier de l'Accélérateur d'impact, les données démographiques du groupe, et s'est terminé en demandant aux participants de s'engager. Les participants se sont engagés à participer activement au réseau de partage des connaissances humaines, à travailler avec d'autres, à partager des informations et à rendre compte régulièrement des progrès accomplis pour transformer les connaissances en actions et améliorer les résultats en matière de santé mondiale, en particulier ceux qui sont liés à la BGF. Ensuite, les facilitateurs ont brièvement partagé des statistiques sur les progrès réalisés par le groupe à ce jour. Pendant les 15 minutes suivantes, les participants ont partagé leurs histoires de mise en œuvre réussie, les leçons tirées des défis présentés et les obstacles existants à la mise en œuvre. Au cours des 30 minutes suivantes, les participants ont fixé des objectifs qu'ils pourraient atteindre dans les deux semaines à venir et en ont discuté, après quoi les facilitateurs de séance ont donné des instructions/conseils sur la manière de communiquer leurs objectifs en ligne. Les expert ont par la suite donné leur avis sur ce qui avait été partagé pendant environ 15 minutes et des conseils sur la définition des objectifs. L'assemblée s'est terminée par un rappel aux participants sur les prochaines étapes.

Rampe de lancement : Assemblée générale 2 (obligatoire) | 13 novembre 2023 (~1 heure)
Accueil et bilan→Partage entre pairs→Retour des experts→Fixation d'objectifs→Retour des experts→Annonces et clôture

La deuxième assemblée générale a commencé par un mot de bienvenue d'environ 10 minutes, une réintroduction de l'engagement pris par tous les participants, un bref aperçu du programme de la phase 2 et un examen des progrès de la première semaine par les facilitateurs de séance.

Texte S1

Pendant les 30 minutes suivantes, les participants ont parlé de leur première semaine - comment elle s'est déroulée pour eux, ce qu'ils ont appris, ce qui a entravé leurs progrès, ce qui les a surpris et quelles seront leurs prochaines étapes. Au cours des 10 minutes suivantes, les experts ont été invitées à répondre directement aux questions des participants, puis à fournir un retour d'information plus général à l'ensemble du groupe sur la manière de progresser en dépit des situations difficiles évoquées. Ensuite, les facilitateurs ont invité les participants à réfléchir à ce qu'ils aimeraient réaliser d'ici la fin de la semaine et d'ici le 15 décembre. Les participants ont partagé pendant environ 10 minutes leurs objectifs à venir et les actions qu'ils entreprendraient pour les atteindre. L'assemblée générale s'est achevée par un retour d'information final d'environ 10 minutes de la part les experts et par des rappels des événements à venir.

Café indépendant à distance (encouragé) | Deux conversations (~15 à 30 minutes chacune)
Pour ce café à distance, les participants ont été jumelés au hasard avec un autre participant qu'ils devaient contacter dans un délai d'une semaine par le biais de leur méthode de communication préférée (par exemple, Zoom, Whatsapp, Messenger). Cela signifie que les participants avaient la possibilité de parler avec deux Scholars, l'un qui les avait contactés et l'autre qu'ils avaient contacté. Bien que les conversations des participants n'aient pas été limitées pendant le café à distance, ils ont été encouragés à garder les conversations sociales et à les limiter à environ 15 à 30 minutes.

Séances éclair (facultatifs) - 15 novembre 2023 et 22 novembre 2023 (~1 heure chacune)
Bienvenue et revue→Témoignage de l'orateur

Les deux séances éclair ont suivi un format similaire. Elles ont commencé par un mot de bienvenue d'environ 5 minutes, une revue (c'est-à-dire de la réalité des BGF, des informations sur le parrainage et la structure de l'événement de formation) et une présentation de l'orateur participant sélectionné par un facilitateur de l'événement. Ensuite, les intervenants ont parlé pendant ~5 minutes sur la BGF dans leurs communautés et ont décrit comment ils avaient travaillé pendant la Rampe de lancement pour atteindre les objectifs qu'ils s'étaient fixés. Pendant les ~35 minutes suivantes, les orateurs ont répondu à la plupart des questions des participants, avec quelques commentaires et questions posées par les experts. Les séances éclair se sont terminées par une synthèse d'environ 5 minutes, des remerciements à toutes les personnes impliquées dans la lutte contre les BGF et des annonces concernant les événements à venir.

Texte S1

Ressources et enregistrements des activités liée à la formation sur la BGF en 2023	
2023 Page web de l'événement spéciale sur la de BGF	https://www.learning.foundation/BGF-fr
Groupe Telegram BGF	https://t.me/+Y43li8lyAdNlN2Q0
Enregistrements des sessions	https://youtube.com/playlist?list=PLti7k0eaN3gRvrPdYVA1gEoyyX10Xkn6c&feature=shared
Compétences de base des BGF	https://reproductive-health-journal.biomedcentral.com/articles/10.1186/s12978-021-01252-2
Ressources pour les apprenants	https://www.dropbox.com/sh/0oze7fv4u6vifpo/AAAPKr5ihUJaM4Bi_0oo-peea?e=1&dl=0
PeerGrade (plateforme en ligne utilisée pour l'évaluation par les pairs et les experts)	https://www.peergrade.io/
Document FAQ BGF	https://zenodo.org/records/8415126
Paquet minimum de services (PMS) pour l'intégration de la BGF<br><br>*Note* : Au cours de la formation, les participants ont discuté de manière informelle de la stigmatisation dans leur contexte. Il ne s'agissait pas d'un thème officiel du programme, mais il a donné lieu à des travaux supplémentaires. Après la formation, Bridges to Development s'est associé à d'autres organisations pour développer et piloter le PMS pour l'intégration de la BGF dans les services de SSR au Kenya, qui a maintenant été publié. Le PMS comporte des points de prestation de services à tous les niveaux et intègre une formation sur la stigmatisation, en particulier au niveau communautaire, dans le cadre de ses composantes relatives à la connaissance de la santé, à l'inclusion sociale et à l'équité.	https://www.eliminateschisto.org/sites/gsa/files/content/attachments/2024-07-08/2023-09-05%20FINAL%20MSP%20-%20Appendix%20A%20ENG%20-clean%2026.6.pdf

Données S1
La matrice de transparence de la BGF

**RÉFLEXION EN GROUPE**<br>**Note : Cette version de la matrice de transparence (Khan, 2022) a été modifiée pour s'adapter au contexte de notre travail collectif sur la BGF. Les réflexions sur les dimensions de la matrice ont été renforcées en répondant aux questions posées par Morton et al. (2022).*			
**Dimension**		**Vecteur d'identité**	**Réflexion**
**Pose**<br>Positionnement épistémique utilisé pour la justification et les relations causales	X<br>X<br>X<br>X<br>X	Établissement universitaire étranger privilégié (élite)<br>Agence mondiale de santé publique (OMS, ONU, etc.)<br>Institution privilégiée (élite) du même pays<br>Institution locale ou entité de recherche<br>Connaissances locales/indigènes	Notre travail est le fruit d'une collaboration entre deux organisations internationales à but non lucratif, La Fondation Apprendre Genève (TGLF) et Bridges to Development, qui soutiennent les réseaux d'apprentissage numérique reliant les travailleurs de la santé locaux à ceux qui sont positionnés à l'étranger.
**Position dans la structure de pouvoir de la recherche**<br>Position dans la structure de pouvoir de la recherche	X<br>X	Agence de financement/ONG<br>Institution universitaire étrangère privilégiée<br>Agence mondiale de santé publique<br>Institution privilégiée (élite) du même pays<br>Institution locale ou entité de recherche<br>Représentant de la population autochtone	Nous occupons des postes allant de stagiaires/assistants de troisième cycle à coordinateurs de programmes et membres fondateurs des deux organisations internationales à but non lucratif qui collaborent. Cette recherche et ses produits (c'est-à-dire les vignettes composites) seront partagés avec les parties prenantes actuelles et potentielles de la santé qui soutiennent les événements de formation et les besoins locaux pour éliminer la BGF.
**Voix**<br>Primauté dans la conception de la recherche	X<br>X<br>X<br>X<br>X<br>X<br>X	Bailleurs de fonds/donateurs<br>Universitaires étrangers<br>Décideurs politiques mondiaux<br>Universitaires d'institutions locales privilégiées<br>Décideurs politiques locaux<br>Universitaires locaux<br>Participants communautaires	Ce travail est financé par le END Fund, qui collabore étroitement avec les parties prenantes de la communauté mondiale des maladies tropicales négligées. La conception et la mise en œuvre des plans d'action décrits dans cette recherche sont basées sur les besoins, les observations et les priorités de la communauté. Les contributions des agents de santé ont éclairé les questions de recherche et la conception que nous avons développées en tant que co-auteurs. Les participants ont été remerciés pour leur participation, leurs réponses à l'enquête et l'élaboration/la mise en œuvre des plans d'action pendant et après les formations. Bien que les participants ne remplissent pas les conditions requises pour être auteurs de ce manuscrit, leur contribution est mentionnée dans la section des remerciements du manuscrit.

Données S1
La matrice de transparence de la BGF

**Regard**<br>Communication s'adressant principalement à	X<br>X<br>X<br>X<br>X	Universitaires internationaux<br>Les décideurs politiques mondiaux<br>Décideurs politiques locaux<br>Universitaires locaux<br>Communauté	Cette publication s'adresse aux acteurs de la santé (praticiens, chercheurs, décideurs politiques, bailleurs de fonds) qui soutiennent les zones d'endémie de la BGF. Les idées sur les réponses cumulatives ont été partagées avec les participants au cours des phases 1 et 2. L'ensemble des données est anonyme et publié sur la page Zenodo de la TGLF pour toute personne, y compris les membres de la communauté, souhaitant en savoir plus (https://doi.org/10.5281/zenodo.15441097.).
**Objectif**<br>Principal objectif analytique utilisé pour tirer des conclusions	 <br>X<br>X	Statistiques<br>Qualitatif/ethnographique<br>Méthodes mixtes<br>Modes locaux/indigènes de prise de conscience<br>Tous les éléments ci-dessus (presque) également	Cette recherche s'appuie également sur des méthodes mixtes. Les données proviennent des contributions des agents de santé locaux, qui rapportent principalement des observations et des expériences directes par le biais d'une narration à la première personne. Ces expériences ont été codées de manière inductive, d'abord en fonction des termes utilisés par les participants, puis affinées en catégories et en thèmes. L'analyse de ces données sous l'angle du connectivisme s'est appuyée sur des citations in extenso. L'intégration des données quantitatives et qualitatives a permis de juxtaposer des statistiques descriptives à des analyses qualitatives et à des citations associées aux niveaux de connectivisme. Les données ont ensuite été analysées afin d'évaluer comment elles se confirmaient, s'enrichissaient, se complétaient ou divergeaient les unes des autres, et de générer des méta-inférences.
**Goût (vérification de la réalité)**<br>Centrage sur l'utilisateur des résultats de la recherche/expérience de l'utilisateur résultant de la mise en œuvre du projet/de la politique		La contribution de l'utilisateur n'est pas requise ou recherchée pour cette recherche/l'expérience de l'utilisateur n'est pas une considération importante de la politique/du projet<br><br>Prise en compte minimale des priorités de l'utilisateur final/expérience désagréable de l'utilisateur résultant de la mise en œuvre de la politique/du projet projet<br><br>Prise en compte modérée des priorités de l'utilisateur final/expérience utilisateur neutre	En participant aux formations, les professionnels de la santé ont accepté de se soutenir mutuellement et de mener des recherches pour les futurs participants et la communauté mondiale. Les événements de formation et cette recherche sont alimentés par leurs contributions. En outre, dans le cadre des formations, les participants ont perfectionné leurs compétences en matière de recherche et de rédaction en fonction de leur contexte. Au cours des phases 1 et 2, ils se sont exercés à

		Implication significative de l'utilisateur final dans les différentes phases de la recherche/expérience positive de l'utilisateur résultant de la la mise en œuvre d'une politique ou d'un projet	utiliser l'analyse des données pour éclairer l'élaboration et la mise en œuvre de leur plan d'action. Ils ont partagé leurs résultats avec leurs pairs, les PME et les animateurs de l'événement afin de recevoir des commentaires et des idées supplémentaires. Ils ont également acquis de l'expérience en donnant des conseils à haute voix et par écrit à leurs pairs.
	X	Recherche initiée par l'utilisateur final/excellente expérience utilisateur résultant de la mise en œuvre d'une politique ou d'un projet	
**Tranche** Identités intersectionnelles pertinentes pour la recherche	X	Statut socio-économique, âge, couleur de peau, sexe, formation virtuelle, ancienneté dans le domaine de la santé mondiale (pratique ou recherche), maîtrise du français, pays de résidence actuel, éducation formelle (obtenue ou activement poursuivie).	Nous sommes un groupe interdisciplinaire de coauteurs ayant des identités diverses et intersectionnelles pertinentes pour cette recherche (voir le deuxième onglet, la position individuelle, et le troisième onglet, la paternité). Collectivement, nous sommes unies dans notre désir d'habiliter et d'équiper les travailleurs de la santé dans les communautés qu'ils servent. Nos expériences en matière de formation virtuelle utilisant la méthodologie d'apprentissage numérique par les pairs de la TGLF ont été positives, et nous apprécions le type d'engagement et les résultats rapportés par les participants. Il s'agit d'un point de vue dont nous avons reconnu qu'il pouvait fausser notre analyse des données. Toutes les étapes de notre processus de recherche ont été participatives afin de tenir compte de cette réalité et de garantir l'analyse et l'interprétation des données à partir de différents points de vue culturels, contextuels, personnels et professionnels. Au cours de nos réunions régulières, nous avons partagé et écouté réciproquement. Au cours de ce processus, nous avons pris conscience des nombreuses similitudes et différences qui existent entre nous et les participants. En collaborant les uns avec les autres au fil du temps, nous avons développé un sentiment de confiance qui nous a permis de remettre en question les perspectives de chacun, et nous avons demandé des examens externes pour obtenir des informations supplémentaires sur d'autres biais potentiels (par exemple, le sexe, l'âge).

Données S1
La matrice de transparence de la BGF

RÉFLEXION INDIVIDUELLE

**Note : Cette matrice a été adaptée de Khan, S. A. (2022).*

Dimension	Vecteur d'identité		KE	CM	RS	JJ	NV	KAN	CP
**Tranche** Identités intersectionnelles pertinentes pour la recherche	Statut socio-économique	Faible revenu		X				X	
		Revenu moyen	X		X	X	X		X
		Revenu élevé							
		Autre/Préfère ne pas répondre							
	Age	<35 ans						X	
		36 à 64 ans	X	X	X	X	X		X
		>65 ans							
		Autre/Préfère ne pas répondre							
	Couleur de la peau	Claire	X			X	X		X
		Moyen			X				
		Sombre		X				X	
		Autre/Préfère ne pas répondre							
	Genre	Homme			X				
		Femme	X	X		X	X	X	X
		Non-binaire / Genre fluide							
		Autre/Préfère ne pas répondre							
	Formation virtuelle	Peu d'expérience (1-3 événements)					X		
		Une certaine expérience (4-6 événements)							
		Grande expérience (7+ événements)	X	X	X	X		X	X
		Autre/Préfère ne pas répondre							
	Durée d'activité dans le domaine de la santé mondiale (pratique ou recherche)	0-5 ans	X					X	
		6-10 ans		X			X		X
		11 ans et plus			X	X			
		Autre/Préfère ne pas répondre							

Données S1
La matrice de transparence de la BGF

Maîtrise du français	Non-locuteur	X			X		X	
	Débutant à intermédiaire					X		
	Avancé à courant		X	X				X
	Autre/Préfère ne pas répondre							
Pays de résidence actuel	Faible revenu		X					
	Revenu moyen							
	Revenu élevé	X		X	X	X	X	X
	Autre/Préfère ne pas répondre							
Éducation formelle (obtenue ou activement poursuivie)	Licence			X		X		
	Maîtrise		X				X	X
	Doctorat	X			X			
	Autre/Préfère ne pas répondre							

Texte S2
Comptes médias des partenaires

**Bridges to Development**	**Comptes médias des partenaires**	**La Fondation Apprendre Genève**
https://bridgestodevelopment.org/	Site web	https://www.learning.foundation/
https://x.com/bridges2develop	Twitter / X	https://x.com/DigitalScholarX
https://www.instagram.com/bridgestodevelopment/	Instagram	https://www.instagram.com/thegenevalearningfoundation/
https://www.linkedin.com/company/bridgestodevelopment/	LinkedIn	https://www.linkedin.com/company/geneva-learning-foundation/mycompany/
	Facebook	https://www.facebook.com/DigitalScholar
	Télégramme	https://t.me/GenevaLearning
	Podcast	https://www.learning.foundation/podcast
	YouTube	https://www.youtube.com/@TheGenevaLearningFoundation
https://zenodo.org/communities/bridgestodevelopment/	Zénodo	https://zenodo.org/communities/tglf/records?q=&l=list&p=1&s=10&sort=newest

Texte S3

Fig A : Intégration des programmes du plan d'action

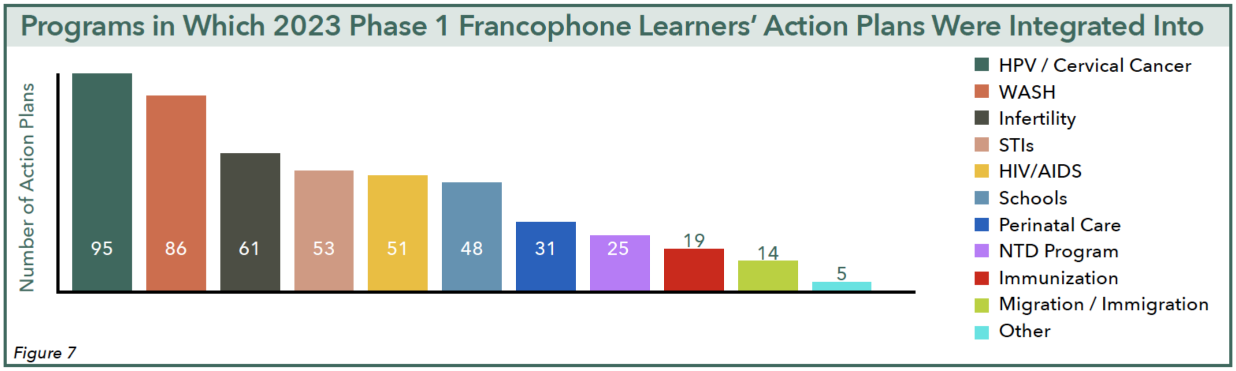

Remarque : Le terme « autres » englobe les programmes qui ne sont pas spécifiquement répertoriés dans la figure 3, mais dans lesquels les participants ont intégré leurs plans d'action (par exemple, le programme de lutte contre le choléra).

Fig B : Objectifs du plan d'action

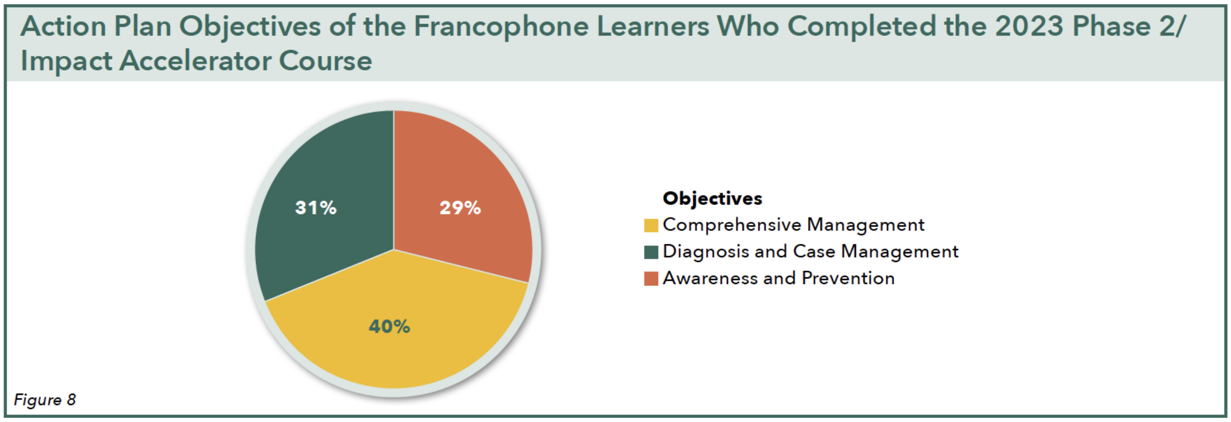

Fig C : Répartition des objectifs du plan d'action (phase 2)

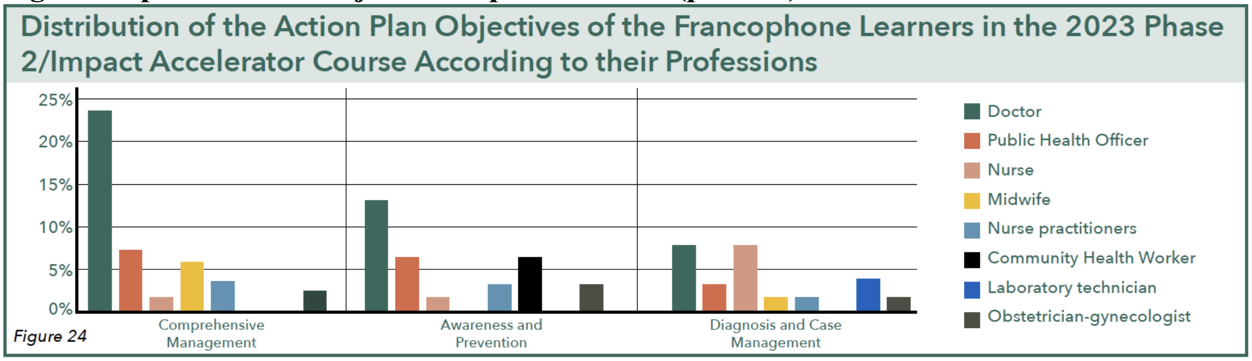

Texte S3

Fig D : Interactions de phase 2 entre les candidats au cours

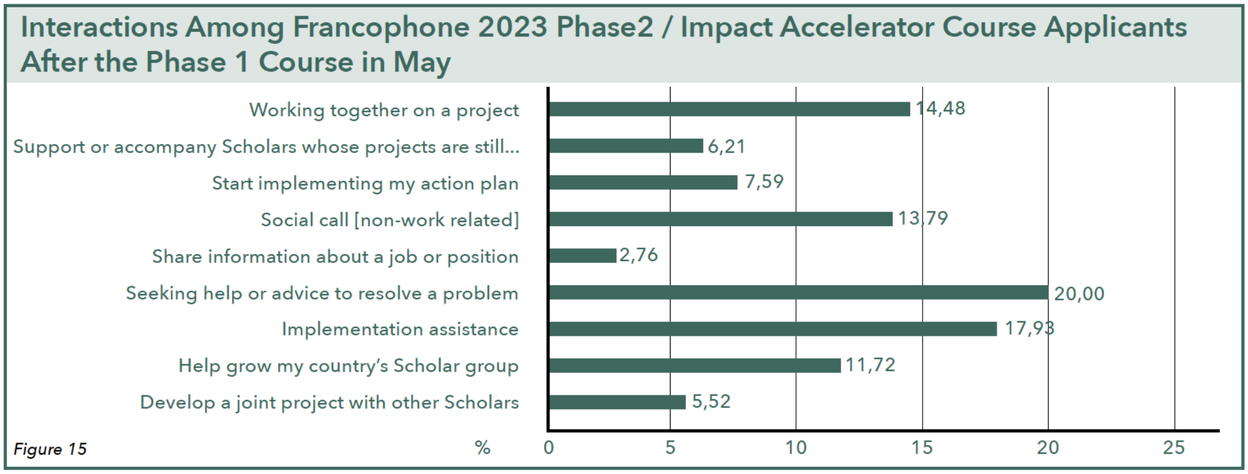

Tableau A : Codes et catégories qualitatifs

Codes	Catégories
Indisponibilité du personnel	Défis de mise en œuvre
Manque de temps	
Matériel, médicaments et données manquants	
Attente d'une réponse ou coordination de celle-ci	
Obtention de financements	
Sécurité et stabilité du site	
Gestion des réticences	
Difficultés liées à la relocalisation, perturbations des déplacements et éloignement du lieu de travail	
Intégration du plan d'action aux activités locales et de district	Stratégies de mise en œuvre
Intégration du plan d'action aux activités nationales	
Plaidoyer auprès des principaux responsables de la santé	
Collaboration avec les agents de santé et les leaders communautaires	
Demande de soutien financier, matériel, de données et médical	
Utilisation de stratégies de communication audio et numérique	
Acquisition de nouvelles informations et techniques	
Utilisation des directives internationales	
Partage de conseils et d'idées avec les pairs	
Conseils et soutien d'experts et de mentors	
Échelonnement du financement et des activités	
Résultats	Résultats de la mise en œuvre
Impacts	

Texte S3

Tableau B : Caractéristiques sociodémographiques du personnel de santé

Catégorie	Démographie	Phase 1 (N=786)	Phase 2 (N= 145)
**Genre**	Les femmes	386 (49.3%)	51 (35.4%)
	Les hommes	397 (50.7%)	93 (64.6%)
**Tranche d'âge (en années)**	18-35	286 (36.4%)	41 (51.9%)
	35 ans et plus	500 (63.6%)	103 (48.1%)
**Région**	Afrique centrale/moyenne<br>Cameroun, République centrafricaine, Congo, République démocratique du Congo, Guinée équatoriale, Gabon	383 (48.7%)	68 (46.9%)
	Afrique du Nord<br>Maroc, Mauritanie, Soudan, Tchad, Tunisie	32 (4.1%)	0 (0.0%)
	Afrique de l'Ouest<br>Bénin, Burkina Faso, Guinée, Côte d'Ivoire, Mali, Niger, Nigeria, Sénégal, Togo	316 (40.2%)	46.20%
	Afrique du Sud<br>Afrique du Sud, Angola, Namibie, Swaziland (Eswatini)	5 (0.6%)	1 (0.7%)
	Afrique de l'Est<br>Burundi, Comores, Djibouti, Éthiopie, Madagascar	43 (5.5%)	9 (6.2%)
	Autres	8 (1.0%)	0 (0.0%)
**Profession**	Médecin/gynécologue- obstétricien	340 (43.4%)	69 (47.9%)
	Agent de santé publique*	171 (21.8%)	23 (16.0%)
	Infirmière/sage-femme/infirmière praticienne	167 (21.2%)	28 (19.4%)
	Agent de santé communautaire	63 (8.0%)	14 (9.7%)
	Technicien de laboratoire	44 (5.6%)	10 (6.9%)
**Organisation**	Hôpital public/établissement de santé	181 (23.1%)	30 (21.4%)
	Personnel du ministère de la santé au niveau national	159 (20.3%)	32 (22.9%)
	Organisation privée**	162 (20.8%)	25 (17.9%)
	Personnel du ministère de la santé au niveau infranational	122 (15.5%)	20 (14.3%)
	Université/institution académique	94 (12.0%)	20 (14.3%)
	Hôpital privé/établissement de santé	66 (8.4%)	13 (9.3%)

Tableau C : Analyse de régression des facteurs influençant l'achèvement de la formation

	**Phase 1**		**Phase 2**	
**Paramètres**	**Rapport de cotes (Intervalle de confiance)**	**Valeur P**	**Coefficient**	**Intervalle crédible (2,5%, 97,5%)**
		**Genre**		
Homme	1		1	
Femme	0.42 (0.22, 0.79)	0.008*	-0.11	-0.99, 0.75
		**Profession**		
Médecin	1		1	
Infirmière	2.86 (0.69, 11.91)	0.149	0.80	-0.27, 1.92
Technicien de laboratoire	1.84 (0.35, 9.77)	0.472	-0.94	-2.76, 0.62

Texte S3

	Phase 1		Phase 2	
**Paramètres**	**Rapport de cotes (Intervalle de confiance)**	**Valeur P**	**Coefficient**	**Intervalle crédible (2,5%, 97,5%)**
Agent de santé publique	1.32 (0.35, 4.96)	0.679	-0.29	-1.52, 0.97
Agent de santé communautaire	0.57 (0.16, 2.06)	0.390	-1.75	-3.58, -0.10*
**Organisation**				
Hôpital public/établissement de santé	1		1	
Hôpital privé/établissement de santé	1.58 (0.52, 4.79)	0.422	0.93	-0.65, 2.62
Université/institution académique	2.03 (0.62, 6.62)	0.243	-1.02	-2.59, 0.42
Personnel du ministère de la santé au niveau national	1.03 (0.43, 2.47)	0.955	-0.48	-1.71, 0.74
Personnel du ministère de la santé au niveau infranational	0.96 (0.34, 2.69)	0.938	-0.23	-1.69, 1.24
Organisation privée	1.37 (0.49, 3.84)	0.552	0.17	-1.31, 1.72
**Effectuer des examens pelviens dans le cadre des responsabilités professionnelles habituelles**				
Non	1		1	
Oui	0.63 (0.3, 1.33)	0.226	-0.63	-1.63, 0.37
**Couverture des frais encourus**				
Ni moi ni mon employeur n'avons pris en charge les frais encourus.	1		-	-
J'ai payé mes dépenses de ma poche.	2.45 (1.26, 4.78)	0.008*	-	-
Mon employeur a pris en charge tous mes frais.	1.08 (0.07, 16.3)	0.956	-	-
Mon employeur et moi-même avons pris en charge une partie des coûts.	3.99 (0.44, 36.51)	0.220	-	-
**Avoir une expérience préalable de la prise en charge de la bilharziose génitale féminine.**				
Non	1		-	-
Oui	0.78 (0.42, 1.46)	0.439	-	-
**Difficultés rencontrées lors de l'atelier**				
A eu des difficultés avec les concepts ou le matériel de formation.	1		-	-
Je n'ai rien trouvé de difficile pour moi.	3.45 (1.15, 10.34)	0.027*	-	-
La formation à la technologie a été difficile.	2.35 (0.72, 7.72)	0.158	-	-
**Le certificat d'atelier sera reconnu par mon employeur**				
Non	1		-	-
Oui	1.61 (0.58, 4.43)	0.359	-	-
**Expérience préalable de l'apprentissage en ligne**				
Faux	1		-	-
Vrai	1.96 (0.69, 5.54)	0.204	-	-
**Première expérience d'apprentissage par les pairs**				
Faux	1		-	-
Vrai	1.56 (0.8, 3.05)	0.196	-	-
**État de la mise en œuvre du plan d'action avant l'accélérateur d'impact**				
Je n'ai pas commencé à mettre en œuvre mon plan d'action.	-	-	1	-
La mise en œuvre de mon plan d'action est en cours.	-	-	1.19	0.13, 2.35*
La mise en œuvre de mon plan d'action est déjà achevée.	-	-	0.64	-1.29, 2.66

	Phase 1		Phase 2	
**Paramètres**	**Rapport de cotes (Intervalle de confiance)**	**Valeur P**	**Coefficient**	**Intervalle crédible (2,5%, 97,5%)**
**Les objectifs de mon plan d'action font partie de mes responsabilités professionnelles**				
Faux	-	-	-	-
Vrai	-	-	-0.15	-1.17, 0.85
**Les objectifs de mon plan d'action ont été intégrés dans un programme ou une initiative existante.**				
Faux	-	-	-	-
Vrai	-	-	0.03	-0.96, 1.03
**Niveau de confiance dans la mise en œuvre du plan d'action avant l'accélérateur d'impact**				
Confiant	-	-	-	-
Pas confiant	-	-	0.65	-0.34, 1.71
Très confiant	-	-	0.80	-0.33, 1.97

Tableau D : Résultats des phases 1 et 2 sur les connaissances, la confiance et la mise en œuvre du plan d'action (N=294*)

**A. Connaissance et confiance**		
	**Préformation**	**Après la formation**
**Connaissance de la BGF**		
Pas de sensibilisation/connaissance	13 (4.4%)	3 (1.0%)
Sensibilisation minimale	46 (15.6%)	8 (2.7%)
Connaissances de base	69 (23.5%)	13 (4.4%)
Connaissance modérée	75 (25.5%)	25 (8.5%)
Connaissances substantielles	38 (12.9%)	106 (36.2%)
Une connaissance approfondie	53 (18.0%)	138 (47.2%)
**Connaissances pour diagnostiquer la BGF**		
Non	176 (59.9%)	28 (9.5%)
Oui	118 (40.1%)	266 (90.5%)
**Connaissances pour traiter la BGF**		
Non	185 (62.9%)	40 (13.61%)
Oui	109 (37.1%)	254 (86.39%)
**Connaissances pour prévenir la BGF**		
Non	125 (42.52%)	10 (3.40%)
Oui	169 (57.48%)	284 (96.60%)
**Confiance en soi pour parler de la BGF avec les patients**		
Pas de confiance	13 (4.4%)	4 (1.4%)
Confiance très faible	51 (17.3%)	5 (1.7%)

Faible confiance	50 (17.0%)	14 (4.8%)
Confiance modérée	67 (22.8%)	21 (7.2%)
Confiance élevée	55 (18.7%)	85 (29.0%)
Confiance totale	58 (19.8%)	164 (55.9%)
**Augmentation individuelle de ≥2 niveaux de connaissance et de confiance**		
Connaissance/diagnostic	-	196 (66%)
Confiance pour parler de la BGF	-	197 (66%)
**B.Plan d'action**		
**Début de la mise en œuvre**		
Après la phase 1	89 (39.0%)	
Avant la phase 2	89 (62.0%)	
Après la phase 2	52 (71.0%)	
**Mise en œuvre achevée**		
Après la phase 1	4 (1.0%)	
Avant la phase 2	9 (6%)	
Après la phase 2	16 (23%)	

Remarque : Nombre de personnes ayant fourni des réponses complètes au questionnaire post-formation.

Tableau E : Portée atteinte après l'activité de la phase 2

Activité	Nombre de personnes touchées	% de participants impliqués
Personnel de santé formé par les participants	2,675	91%
Patients diagnostiqués/pris en charge	638	39%
Les membres de la communauté sont informés sur la BGF	49,088	82%

Tableau F : Régression logistique des facteurs influençant les gains d'apprentissage

Paramètres	Rapport de cotes (Intervalle de confiance)	Valeur P
**Genre**		
Homme	1	
Femme	1.18 (0.6, 2.34)	0.628
**Profession**		
Médecin	1	
Infirmière	1.11 (0.45, 2.77)	0.819
Technicien de laboratoire	0.64 (0.19, 2.22)	0.486
Agent de santé publique	0.54 (0.22, 1.36)	0.193
Agent de santé communautaire	0.17 (0.04, 0.67)	0.012*
**Effectuer des examens pelviens dans le cadre des responsabilités professionnelles habituelles**		

Paramètres	Rapport de cotes (Intervalle de confiance)	Valeur P
Faux	1	
Vrai	0.81 (0.37, 1.77)	0.598
**Organisation**		
Hôpital public/établissement de santé	1	
Hôpital/établissement de santé privé	1.18 (0.29, 4.8)	0.816
Université/institution académique	0.32 (0.1, 1.06)	0.061
Personnel du ministère de la santé au niveau national	0.35 (0.12, 1.02)	0.055
Personnel du ministère de la santé au niveau infranational	0.39 (0.12, 1.25)	0.114
Organisation privée	0.67 (0.2, 2.21)	0.506
**Avoir une expérience préalable de la prise en charge de la bilharziose génitale féminine.**		
Faux	1	
Vrai	0.17 (0.04, 0.71)	0.015*
**Difficultés rencontrées lors de l'atelier**		
A eu des difficultés avec les concepts ou le matériel de formation.	1	
Je n'ai rien trouvé de difficile pour moi.	0.6 (0.14, 2.6)	0.494
La formation à la technologie a été difficile.	0.77 (0.15, 3.83)	0.748
**Le certificat d'atelier sera reconnu par mon employeur**		
Faux	1	
Vrai	0.16 (0.02, 1.38)	0.097
**Environnement de travail**		
Ruralité	1	
Urbain	1.2 (0.63, 2.29)	0.576

Tableau G : Régression du modèle d'apprentissage entre pairs (Influence du modèle sur l'acquisition des connaissances/l'évolution des connaissances/les niveaux de diagnostic)

**Paramètres**	**Coefficient**	**Intervalle crédible (2,5%, 97,5%)**
**Première expérience d'apprentissage entre pairs**		
Faux		
Vrai	-0.1	-0.65, 0.43
**Impact de la revue par les pairs sur l'acquisition des connaissances**		
Je n'ai pas appris.		
J'ai appris plus que je ne l'espérais	-0.45	-1.83, 0.83
J'ai appris moins que ce que j'attendais	-0.48	-2.09, 0.98
J'ai appris ce que j'attendais	-0.77	-2.20, 0.61
**Le soutien des pairs a été utile**		

Faux		
Vrai	1.97	0.80, 3.30*
**Impact de la revue par les pairs du travail des collègues**		
Je ne l'ai pas fait		
Inutile	1.65	-1.12, 5.32
Utile	0.03	-1.04, 1.08
Très utile	0.16	-0.86, 1.14

Tableau H : Acquisition de connaissances et élargissement de la portée

**Résultats quantitatifs**	**Résultats qualitatifs**	**Méthodes mixtes de méta-inférences**
Amélioration des connaissances et de la confiance : 66% des participants à la phase 1 ont signalé une amélioration de leurs connaissances sur la BGF et de leurs capacités de diagnostic, ainsi qu'une plus grande confiance en eux pour discuter de la BGF.<br><br>Cependant, les agents de santé communautaires avaient 83% de chances en moins d'atteindre des niveaux de connaissances plus élevés que les médecins/gynécologues-obstétriciens. De même, les participants ayant une expérience préalable de la prise en charge de la bilharziose génitale féminine avaient 83% de chances en moins de faire état de tels progrès, par rapport à ceux qui n'en avaient pas.	Connexions neuronales-BGF Connaissances et compétences techniques acquises : La phase 1 a permis d'acquérir de nouvelles connaissances, de renforcer la compréhension et la confiance existantes et de remédier à la désinformation.<br><br>*« J'ai presque dix ans d'expérience professionnelle dans la lutte contre les MTN, et ce n'est qu'au cours de cette formation que j'ai appris le concept de la BGF. »*<br>-Responsable de la santé publique au sein d'un ministère de la santé<br><br>Connexions sociales/externes - Diverses connexions et ressources pour la croissance et l'impact : Les participants ont obtenu des responsabilités plus importantes, plus de patients et une plus grande confiance de la part de la communauté.<br><br>*«Je suis confiant dans ce que je fais et mes collègues ont confiance en moi lorsqu'il s'agit de la bilharziose, par rapport à avant. »* - Agent de santé publique travaillant dans un hôpital/établissement de santé privé	◊Complémentarité : L'augmentation des connaissances et de la confiance, parallèlement aux disparités en fonction du rôle et de l'expérience, est complétée par des récits montrant comment l'apprentissage a corrigé les informations erronées, renforcé la crédibilité et la confiance professionnelle.
Un large impact et une portée amplifiée : Après la phase 2, 39% des participants ont diagnostiqué ou pris en charge des patients atteints de la BGF, soit environ 638 cas. 91% des participants ont déclaré avoir formé un total de 2675 collègues. En outre, 82% des participants se sont	Connexions neuronales-BGF Connaissances techniques et compétences acquises : Les connaissances et la confiance acquises ont permis aux participants de partager des informations avec leurs collègues, tandis que les	◊Confirmation : Les patients atteints, les collègues formés et les communautés engagées sont confirmés par des récits décrivant le partage des connaissances et la crédibilité professionnelle.

engagés auprès de leurs communautés, atteignant plus de 49000 personnes avec des informations sur la BGF.	compétences acquises ont permis d'identifier et de diagnostiquer le BGF.<br><br>*«J'ai sensibilisé sur les réseaux sociaux. J'ai même commencé à stimuler les (réflexions) de mes collègues ainsi que des sage-femmes et des infirmières obstétriciennes sur les questions de la BGF dans les différents canaux de communication comme WhatsApp, Telegram et sur Facebook. »* - Médecin travaillant dans un hôpital/établissement de santé privé<br><br>Connexions sociales/externes - Diverses connexions et ressources pour la croissance et l'impact : La confiance a été renforcée, ce qui a motivé les participants à effectuer des changements et à partager leurs connaissances par le biais de l'engagement communautaire et de la documentation de cas.<br><br>*«Cette formation m'a permis de comprendre que je suis désormais un citoyen du monde et que le simple conseil que je donne à un collègue peut lui être utile pour résoudre un problème de santé publique ou sauver une vie à l'autre bout du monde. »* - Agent de santé publique travaillant dans une organisation à but non lucratif	▲Expansion : La vaste portée de la phase 2 est élargie par des récits qualitatifs montrant comment la confiance et les compétences ont été transformées en plaidoyer, en partage de connaissances et en influence plus large.

Tableau I : Élaboration et mise en œuvre du plan d'action

**Résultats quantitatifs**	**Résultats qualitatifs**	**Méta-inférences issues de méthodes mixtes**
Diversité des plans d'action adaptés aux responsabilités professionnelles : Trois grandes catégories de plans d'action ont émergé lors de la phase 2 : sensibilisation et prévention, diagnostic et prise en charge, et prise en charge globale. Les médecins étaient responsables de la plupart des plans de prise en charge globale, les agents de santé communautaires se concentraient sur la sensibilisation et les techniciens de laboratoire sur le diagnostic. Les examens pelviens étaient pratiqués	Thème 2 – Connexions connectivistes malgré les complexités : Les raisons pour lesquelles les plans d'action n'ont pas été menés à terme incluent l'attente de données, de financement ou d'autorisation, l'ajustement des échéanciers pour les activités personnelles ou communautaires, la planification de projets multirégionaux qui nécessitaient plus de temps et un manque de médicaments BGF disponibles.	◊ Complémentarité : La répartition des types de plans d'action et leur alignement avec les responsabilités professionnelles sont complétés par des comptes rendus soulignant les obstacles pratiques qui ont façonné la mise en œuvre, notamment les lacunes en matière de ressources, les retards d'autorisation et les pénuries de médicaments.

par 60 % des personnes ayant des objectifs de diagnostic, 68,4 % ayant des objectifs de prise en charge globale et 35,9 % ayant des objectifs de sensibilisation.		
	Thème 1 – Accélérer les connexions et l'intégration : L'intégration a accru la probabilité de mise en œuvre du plan d'action en facilitant le plaidoyer, en favorisant la collaboration et en tirant parti des services et programmes existants.	◊Confirmation : L'intégration était la stratégie prédominante dans l'ensemble des plans d'action et a été systématiquement présentée comme un élément central de leur mise en œuvre.<br><br>▲Complémentarité : L'intégration a été expliquée plus en détail à travers des exemples montrant comment elle a permis de plaider la cause, de favoriser la collaboration et de tirer parti des services et programmes existants.
Renforcement des capacités de mise en œuvre des plans d'action : À la fin de la phase 1, 39% des participants avaient entamé leur plan d'action et 1% l'avaient finalisé. Après la phase 2, ces chiffres étaient respectivement de 71% et 23%.	Liens conceptuels – Apprentissage par les pairs et réseautage pour renforcer la planification des actions : L'élaboration et la mise en œuvre des plans d'action ont été renforcées par la revue par les pairs, le réseautage et un engagement continu après la phase 1. Les obstacles tels que le manque de financement, de médicaments et d'adhésion des autorités sanitaires ont été surmontés grâce au plaidoyer, au soutien des pairs et à la consultation d'experts.<br><br>*« Lorsque je n'ai pas pu déployer mon plan d'action faute de moyens financiers, j'étais désorientée. C'est alors qu'en suivant des témoignages en direct et en lisant des articles sur Telegram, j'ai découvert les méthodes utilisées par d'autres Scholars, confrontés aux mêmes difficultés financières, pour atteindre les populations à risque. Ces connaissances m'ont permis de revoir mon approche. Il est clair que lorsque nous participons à un tel rampe (phase 2), nous sommes influencés par de nouvelles idées et par d'autres Scholars issus de divers horizons. »* – Agent de santé communautaire travaillant au niveau du district	◊Complémentarité : Les résultats quantitatifs ont démontré une capacité accrue de mise en œuvre du plan d'action après la phase 2, tandis que les résultats qualitatifs ont complété cela en expliquant les processus qui ont permis la mise en œuvre, notamment l'apprentissage entre pairs, le réseautage et le plaidoyer pour surmonter les obstacles.

Tableau J : Impact du modèle d'apprentissage par les pairs jusqu'à l'action

**Résultats quantitatifs**	**Résultats qualitatifs**	**Méta-inférences issues de méthodes mixtes**
Le soutien des pairs a influencé l'acquisition de connaissances : Les personnes qui ont déclaré que le soutien par les pairs était utile avaient une probabilité logarithmique plus élevée d'accroître leur niveau de connaissances que celles qui n'ont pas déclaré que le soutien par les pairs était utile.	Connexions sociales/externes - Diverses connexions et ressources pour la croissance et l'impact : Les interactions entre pairs ont favorisé la confiance, approfondi l'intérêt pour la BGF, amélioré les capacités d'écoute et soutenu la réflexion critique sur la collaboration transfrontalière.<br><br>*« Comme je ne suis pas un professionnel de la santé... parler de la profession... avec des collègues d'autres pays a enrichi mes connaissances (et)... j'ai renforcé mes capacités dans le domaine de la santé. »* - Agent de santé communautaire d'une organisation non gouvernementale	◊Confirmation : Le soutien des pairs a été identifié comme un moteur de l'acquisition de connaissances et a été confirmé par les récits décrivant la confiance, l'intérêt plus profond et les capacités renforcées grâce à l'interaction avec les collègues.<br><br>▲Complémentarité : Les résultats concernant le soutien par les pairs ont été complétés par des récits faisant état d'avantages supplémentaires, notamment l'amélioration des capacités d'écoute, de la pensée critique et de la collaboration transfrontalière.
Impact des interactions avec les pairs : Parmi les participants à la phase 1, 61% ont indiqué dans l'enquête post-formation qu'ils avaient appris plus qu'ils ne l'avaient prévu grâce à la revue par les pairs. 85% ont indiqué qu'ils avaient connu des changements significatifs dans leur pratique professionnelle à la suite de leur participation.	Connexions sociales/externes - Diverses connexions et ressources pour la croissance et l'impact : Le programme a rendu les participants plus proactifs, a amélioré leurs performances professionnelles et a élargi les réseaux qui leur ont apporté un soutien, un accès rapide à l'information, une collaboration et un plaidoyer plus fort auprès des autorités sanitaires.<br><br>*« Ma participation m'a changé en tant que professionnel. En effet, j'ai réalisé que plusieurs maladies tropicales négligées, notamment la BGF, sont en réalité des problèmes de santé publique et que le fait de ne pas y penser aggrave l'état de santé. Pourtant, si on les prenait en compte dès le départ, cela éviterait aux patients de dépenser beaucoup d'argent pour leur état de santé. »* - Médecin travaillant dans un hôpital/établissement de santé privé	Confirmation : Les revues par les pairs ont amélioré l'apprentissage et la pratique professionnelle, ce qui a été confirmé par des témoignages faisant état d'une plus grande proactivité, d'une amélioration des performances et d'un renforcement de la défense des intérêts.<br><br>▲Complémentarité : Les changements signalés dans la pratique ont été complétés par des récits montrant comment les interactions entre pairs ont élargi les réseaux, favorisé la collaboration et modifié la perception de la BGF comme un problème de santé publique préoccupant.
Influence du modèle sur l'élaboration et la mise en œuvre des plans d'action : 86% des personnes interrogées ont déclaré que la revue par les pairs avaient considérablement amélioré leurs propres plans d'action, tandis que 85% ont estimé que la revue des plans d'action de leurs collègues avait été bénéfique.	Liens conceptuels – Apprentissage entre pairs et réseautage pour renforcer la planification des actions : Le soutien par les pairs a été largement apprécié, beaucoup le jugeant plus bénéfique que les documents/ressources. Les interactions formelles et informelles entre pairs ont amélioré l'élaboration des plans d'action, clarifié les attentes et soutenu leur mise en œuvre grâce à un engagement continu et au partage d'expériences.	◊Confirmation : Il a été démontré que les évaluations par les pairs améliorent les plans d'action, comme en témoignent les retours d'information qui ont directement renforcé le contenu et la clarté des plans.<br><br>▲Complémentarité : La valeur du soutien par les pairs a été complétée par des témoignages illustrant comment les interactions,

	« L'évaluation par les pairs m'a permis d'améliorer mon plan d'action. Par exemple, un pair m'a rappelé que le titre de mon plan devait commencer par un verbe d'action. Un autre m'a suggéré de trouver une meilleure carte des districts sanitaires… car celle que j'avais incluse dans le plan d'action initial manquait de clarté. Ces deux exemples, parmi tant d'autres, illustrent à quel point l'évaluation par les pairs demeure essentielle. » – Responsable de la santé publique dans un organisme sans but lucratif	formelles et informelles, ont fourni des conseils pratiques, favorisé l'apprentissage et assuré la pérennité de la mise en œuvre.
Maintien de l'engagement après la formation : Une proportion importante de participants (77,2%) a contacté ses pairs après la phase 1 à des fins de collaboration. Parmi eux, 61% ont également attribué le maintien du contact avec leurs pairs après le programme d'apprentissage par les pairs.	Liens sociaux et externes – Diversité des liens et des ressources pour la croissance et l'impact : Les réseaux de pairs, tant au niveau national qu'international, ont apporté un soutien, un accès rapide à l'information, une collaboration et un plaidoyer collectif qui ont permis de surmonter les difficultés de mise en œuvre et d'atteindre les objectifs.<br><br>*« Je suis entouré d'un vaste réseau de praticiens du monde entier, certes virtuel, mais je sais que je peux obtenir de l'aide à tout moment. »* – Médecin au ministère de la Santé	◊Confirmation : L'engagement post-formation a été confirmé par des témoignages décrivant des réseaux de pairs actifs favorisant la collaboration et le soutien mutuel.<br><br>▲Complémentarité : Ces résultats ont été complétés par des témoignages illustrant comment les réseaux ont permis un accès rapide à l'information, des échanges internationaux et un plaidoyer collectif.
